# AmeriFlux BASE data pipeline to support network growth and data sharing

**DOI:** 10.1038/s41597-023-02531-2

**Published:** 2023-09-11

**Authors:** Housen Chu, Danielle S. Christianson, You-Wei Cheah, Gilberto Pastorello, Fianna O’Brien, Joshua Geden, Sy-Toan Ngo, Rachel Hollowgrass, Karla Leibowitz, Norman F. Beekwilder, Megha Sandesh, Sigrid Dengel, Stephen W. Chan, André Santos, Kyle Delwiche, Koong Yi, Christin Buechner, Dennis Baldocchi, Dario Papale, Trevor F. Keenan, Sébastien C. Biraud, Deborah A. Agarwal, Margaret S. Torn

**Affiliations:** 1 Climate & Ecosystem Sciences Division, Lawrence Berkeley National Laboratory, Berkeley, CA 94720 USA; 2 Scientific Data Division, Lawrence Berkeley National Laboratory, Berkeley, CA 94720 USA; 3 Department of Environmental Science, Policy, and Management, University of California Berkeley, Berkeley, CA 94720 USA; 4 HyperArts, Inc, Oakland, CA 94607 USA; 5 Department of Computer Science, University of Virginia, Charlottesville, VA 22903 USA; 6 DIBAF, University of Tuscia, Viterbo, 01100 Italy; 7 Euro-Mediterranean Center on Climate Change CMCC IAFES, Viterbo, 01100 Italy; 8 Energy and Resources Group, University of California Berkeley, Berkeley, CA 94720 USA

**Keywords:** Carbon cycle, Atmospheric dynamics, Projection and prediction

## Abstract

AmeriFlux is a network of research sites that measure carbon, water, and energy fluxes between ecosystems and the atmosphere using the eddy covariance technique to study a variety of Earth science questions. AmeriFlux’s diversity of ecosystems, instruments, and data-processing routines create challenges for data standardization, quality assurance, and sharing across the network. To address these challenges, the AmeriFlux Management Project (AMP) designed and implemented the BASE data-processing pipeline. The pipeline begins with data uploaded by the site teams, followed by the AMP team’s quality assurance and quality control (QA/QC), ingestion of site metadata, and publication of the BASE data product. The semi-automated pipeline enables us to keep pace with the rapid growth of the network. As of 2022, the AmeriFlux BASE data product contains 3,130 site years of data from 444 sites, with standardized units and variable names of more than 60 common variables, representing the largest long-term data repository for flux-met data in the world. The standardized, quality-ensured data product facilitates multisite comparisons, model evaluations, and data syntheses.

## Introduction

AmeriFlux is a network of research sites and scientists that use the eddy-covariance technique to measure ecosystem carbon, water, energy, and momentum fluxes in ecosystems across the Americas^1^. It was established in 1996 to connect independently-managed research in these diverse ecosystems, thus jointly representing major climatic and ecological contexts. Over the last few decades, AmeriFlux has been at the forefront of land-atmosphere interaction research, committed to collecting and sharing high-quality flux and meteorological (flux-met) data among the community of flux researchers. This broader AmeriFlux community of both site teams and data users contributes to science in many ways, including fundamental research, Earth system model development, data science, technical innovation, and science education. For example, AmeriFlux data are widely used to benchmark, validate, and develop new algorithms in the land models of Earth system models^2,3^. Remote-sensing scientists use AmeriFlux data to parameterize and validate models to upscale carbon and water fluxes in space and time^4–6^. The biogeochemistry and ecology communities use AmeriFlux data to construct budgets of elements with high precision and sampling frequency^7–9^ and identify new and emerging processes, such as the divergence/convergence of ecosystem functions (e.g., carbon uptake, water use, carbon use, energy partition) across space and time^10–13^. Long-term AmeriFlux data are valuable in assessing ecosystem carbon sequestration, water and energy budget, and response to climate change, disturbances, management practices, and climatic extremes^14–17^. The impact of research based on AmeriFlux data goes beyond these examples and continues to grow, integrating processes across disciplines and spatiotemporal scales.

**Fig. 1:**
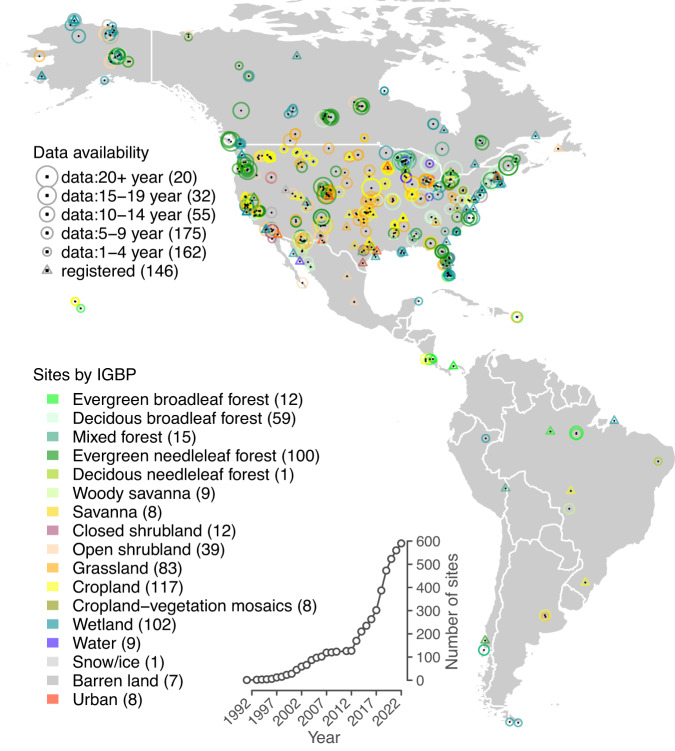
Map of AmeriFlux sites. Triangles represent registered sites (146) with no available data, and circles represent sites (444) with data available through AmeriFlux. The circles’ size indicates the length of the data record. The color of the circles represents the ecosystem type based on the International Geosphere-Biosphere Programme (IGBP) definition. Parentheses indicate the number of sites in each data availability and IGBP group. The inset shows the cumulative number of registered sites over the years. All numbers are updated as of the end of 2022.

Since its launch in 1996, AmeriFlux has grown from 15 sites to >110 in 2012 when the AmeriFlux Management Project (AMP, see below) was established, and to 590 sites at the end of 2022 (Fig. 1). These sites represent a broad spectrum of ecosystems across climatic and ecological gradients and diverse regimes of natural disturbance and human management (Fig. 1, Supplementary Figure S1). AmeriFlux is distinguished among all flux networks by having more than 100 sites with times series longer than a decade, including several of the longest-running sites in the world (e.g., Harvard Forest (US-Ha1, 1991-current), Borden Forest (CA-Cbo, 1994-current), Park Falls (US-PFa, 1995-current), Howland Forest (US-Ho1, 1995-current)). These long flux records allow scientists to address questions requiring decades of observations^18^, such as understanding ecosystem response to climate variability and atmospheric change^14,15,19,20^. AmeriFlux also contains many clusters of neighboring sites established by individual research groups^21^. Driven by research questions, many site clusters were established across gradients of land cover and land use, chronosequence stages, microclimate, management, disturbance, and restoration^22–25^. The site clusters enable the research communities to understand how different ecosystems respond to similar climatic and, in some cases, edaphic conditions. Moreover, measurements across wide environmental gradients can be constructed from the network’s sites at a regional or continental scale. This distinctive cluster/gradient design makes AmeriFlux data a powerful testbed for model benchmarking, assessing the effects of climate and land cover and land use changes^26,27^.

AmeriFlux’s wide diversity of ecosystems, instruments, data-processing routines, and science activities are both its strength and challenge. AmeriFlux sites are established by individual site teams driven by diverse research needs and questions^1^. As a result, research designs and measurements vary among sites, being tailored to each ecosystem and project. This individuality distinguishes AmeriFlux from other flux networks, such as the National Ecological Observatory Network (NEON) and Integrated Carbon Observation System (ICOS^28^), which have standardized instrument packages and data-processing protocols^29–33^. AmeriFlux’s diverse and innovative nature has enabled the network to evolve and adapt to new technology when available (and promote that evolution)^34–37^. However, the diversity in approaches also challenges data standardization, quality assurance, and data sharing across the network.

In 2012, the United States Department of Energy (DOE) established AMP at the Lawrence Berkeley National Lab (LBNL) to support the broader AmeriFlux community, composed of the AmeriFlux site teams that produce flux-met data and the researchers who use these data. AMP collaborates with AmeriFlux researchers to ensure the quality and availability of the continuous, long-term measurements necessary to understand ecosystems and to build effective models and multisite syntheses. To achieve these goals, AMP has established technical, data, and outreach services, held annual meetings and workshops, and provided operational support to 13–14 flux site clusters (Core sites) to ensure public access to high-quality and long-term flux-met datasets. AMP further supports the community by creating new opportunities (e.g., AmeriFlux Annual Meetings, theme years, working groups, synthesis workshops, webinars) for AmeriFlux researchers to contribute to high-impact research.

AMP’s data support centers on developing standards, data QA/QC, data-processing, and data repositories. AMP worked collaboratively with international partners, particularly ICOS, to design and develop standard formats and processing routines. In 2017, the AMP team at LBNL took full responsibility for the AmeriFlux data repository, previously maintained by the Carbon Dioxide Information Analysis Center (CDIAC) at the Oak Ridge National Lab (ORNL). With that, AMP redesigned, implemented, and launched the new BASE data-processing pipeline (details below), with the objectives of (1) standardizing the flux-met data formats, (2) ensuring and improving the data quality, (3) facilitating regular and frequent data submissions and publications, and (4) tracking the data and communications with site teams through the pipeline. The following sections summarize the outcome of the data-processing pipeline. The methodology behind its design and implementation are detailed in the Methods.

## Results

**Fig. 2:**
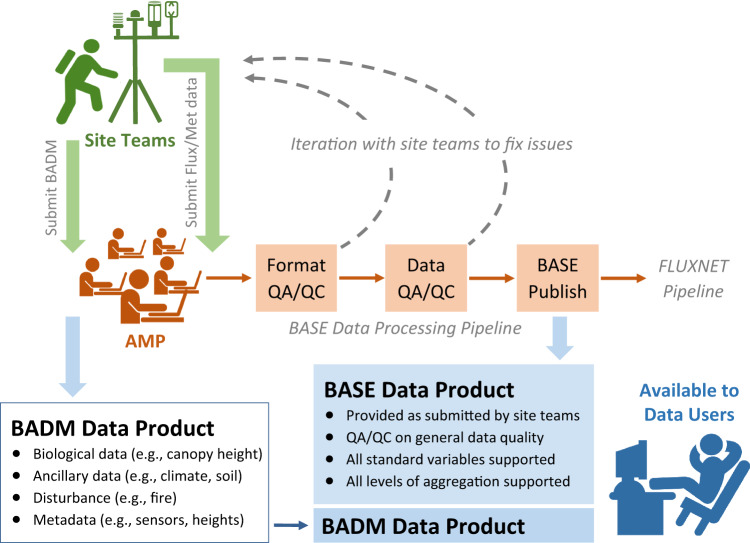
AmeriFlux BASE data-processing pipeline. The pipeline begins with data uploads from the site teams, followed by steps of Format QA/QC, Data QA/QC, and BASE Publish performed by AmeriFlux Management Project (AMP). The end products are the flux/met data (BASE) and Biological, Ancillary, Disturbance, and Metadata (BADM) products. Green, brown, and blue colors in the figure represent actions taken by site teams, AMP, and data users, respectively. While not detailed in this article, the BASE and BADM products can be used as input to the FLUXNET processing pipeline^42^.

The BASE data-processing pipeline begins with site teams submitting their flux-met data in a standardized format, followed by a series of quality assurance and quality control (QA/QC) checks performed by AMP, e.g., Format QA/QC for format compliance and Data QA/QC for data quality (Fig. 2). AMP then communicates the check results and, if any, needed corrections with site teams through Format and Data QA/QC reports. Once passing QA/QC checks, the flux-met data are published as the BASE data product for each site, i.e., made publicly available on the AmeriFlux website. The BASE data format follows an international standard compatible with other flux networks like ICOS and European Database.

### Data upload and release

**Fig. 3:**
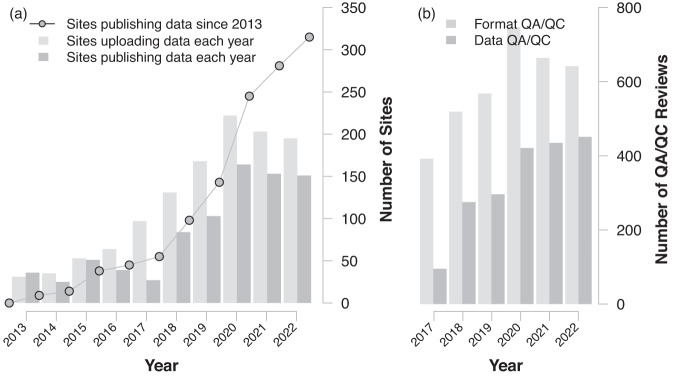
Records of data uploads, QA/QC reviews, and data published. Light- and dark-gray bars in Figure (**a**) show the number of sites uploading and publishing data each year since 2013. Gray circles show the cumulative number of sites publishing data since 2013. Light- and dark-gray bars in Figure (**b**) show the number of Format and Data QA/QC reports since implementing the BASE data-processing pipeline in 2017. All numbers are updated as of the end of 2022.

Between implementing the pipeline in May 2017 and December 31, 2022, we have received 3,468 data uploads containing 6,195 files of flux-met data from 385 sites (Fig. 3a). AMP generated 3,538 Format QA/QC and 1,980 Data QA/QC reports that were emailed to site teams (Fig. 3b). Notably, in 2020–2022, we received data uploads from ~200 sites each year and sent more than 600 and 400 Format and Data QA/QC reports yearly. As a reference, the BASE data repository contained 1,256 site-years of data from 174 sites in April 2017. The 2017–2022 period coincided with the rapid growth of the network (Fig. 1). The implemented pipeline enables us to keep up with the growth, publishing on average ~48 new sites and ~330 new site years each year. As of 2022, there are 3,130 site years of AmeriFlux BASE data from 444 sites, representing the world’s largest data repository for flux-met data. Moreover, 344 sites (~77%) are under the CC-BY-4.0 license.

During 2017–2022, 288 sites submitted data for the first time and were checked by the Format and Data QA/QC. Around 94% of these new sites’ data was published in the BASE data product as of 2022. For each site’s first complete publishing cycle, these new sites took a median of 127 days from the first-time data upload to BASE publication. The durations varied depending on the number of iterations required to resolve the identified issues, particularly in the Data QA/QC. While varied among sites, common data issues include shifts in timestamps, sensor degradation, excessive outlier, incorrect units, and flipped sign conventions. About 29%, 33%, and 28% of sites went through 1–3 (re)submission cycles, with median durations of 60, 116, and 154 days, respectively. This latency time, especially for the new sites, is reflected in the difference between the number of sites uploading and publishing data within each year (Fig. 3a).

Around 217 sites updated their BASE data product (e.g., adding additional years of data to previously published BASE) in 2017–2022, including 150 new sites discussed above. The median turnaround duration was around 42 days from upload to BASE publication, much shorter than the first-time submissions from the new sites. Most (80%) of these returning sites took less than 90 days from the upload to BASE publication. Seventy-five sites updated their BASE data product more than five times in 2017–2022.

In sum, the BASE pipeline facilitates more frequent data uploads and releases and allows data users to access recent-year data. While traversing the pipeline entailed a few iterations and months for new sites to address the identified issues, it significantly decreased the overall latency time between data collection and release for many returning sites. For example, the number of sites with data available for the prior year increased from 0 sites in 2017 to 90 sites in 2022 (Supplementary Figure S2). Over 2017–2022, the BASE data products were downloaded more than 27,000 times by ~4,800 users globally. Many of these downloads included multiple sites, resulting in total site downloads of 318,553 for the period. Notably, the total site downloads increased from 18,644 in 2017 to 86,371 in 2022. The data-download interface logs the downloader’s intended data use, and these covered a wide range^38^, such as multisite synthesis, benchmarking remote-sensing and land surface models, and education.

### Data summary

**Fig. 4:**
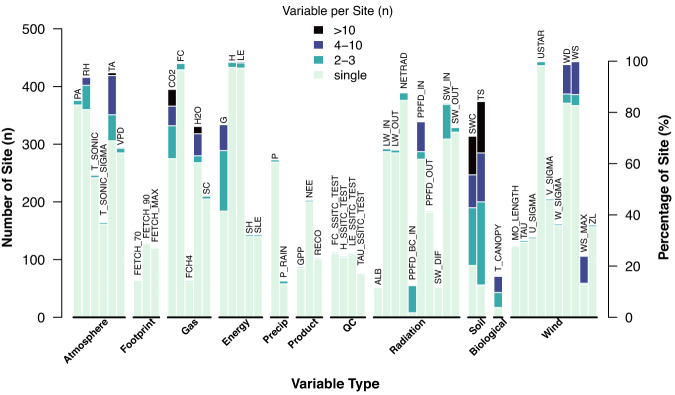
BASE data variable availability by variable types. Bars indicate the numbers (left y-axis) and percentages (right y-axis) of sites. Annotated texts denote the variable names. The colors of the bars indicate the number of unique variables (e.g., locations, sensors) at a site. The figure only contains the most commonly available variables (i.e., >50 sites). Variable definitions and units refer to Supplementary Table S1. All numbers are updated as of the end of 2022.

The BASE data pipeline generates the BASE data product: time series flux-met data at a half-hourly or hourly resolution. The BASE data product follows the global FP (Flux Processing) Standard^39^, ensuring that variable names, units, and file formats are defined and consistent. Around 52 out of 143 variables supported by the FP Standard are commonly submitted (>50 sites, Fig. 4, Supplementary Table S1). These variables can be categorized into flux-related groups, such as the trace gasses (e.g., CO_2_ and CH_4_ fluxes and concentrations), energy (e.g., latent and sensible heat fluxes), derived products (e.g., gross primary production, ecosystem respiration), quality flags (e.g., steady-state and integral turbulence characteristics), and footprints (e.g., distance with maximum footprint contribution). The BASE data product also consists of data on meteorology and soil, such as the groups of radiation (e.g., net radiation, incoming shortwave radiation), atmosphere (e.g., air temperature, relative humidity), wind (e.g., friction velocity, wind speed), precipitation, and soil (e.g., soil temperature, soil water content). It is worth mentioning that some sites have data measured at multiple locations (dark colors in Fig. 4) for replication or spatial variation. In particular, soil temperature and water content are measured extensively in vertical or horizontal locations at most sites. Air temperature, wind speed, direction, CO_2_ and H_2_O concentrations, and soil heat fluxes are also measured at multiple locations at around 80–120 sites.

**Fig. 5:**
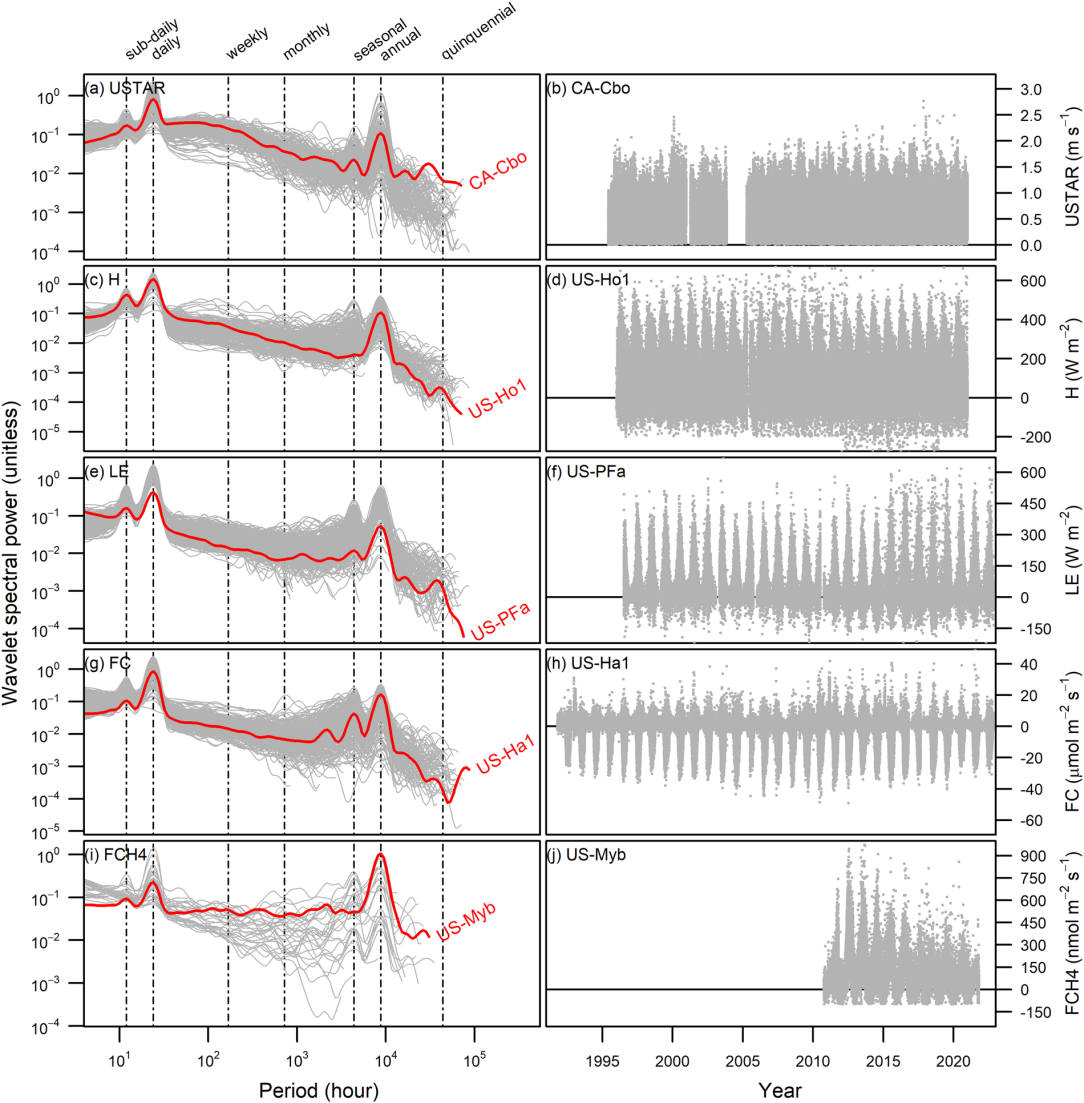
Wavelet power spectra (left panel) and time series (right panel) of flux variables. From top to bottom, the variables are (**a,b**) friction velocity (USTAR), (**c,d**) sensible heat flux (H), (**e,f**) latent heat flux (LE), (**g,h**) CO_2_ flux (FC), and (**i,j**) CH_4_ flux (FCH4). Each gray line in the left panel represents a power spectrum from an AmeriFlux site, including all sites under the CC-BY-4.0 data license and with >25% of data coverage. The red lines highlight the power spectra from the selected long-term sites, annotated with their site ID. The right panel shows the time series of flux variables from the selected long-term sites. Wavelet power was presented in logarithmic scales (left y-axis) and rectified to eliminate bias to allow comparisons among the periods^69^. All numbers are updated as of the end of 2022. See Table 1 for a complete list of sites and Supplementary Table S2 for their data years and citations.

BASE flux-met data are rich time series, typically with half-hour resolutions and data records that span from years to decades. While a portion of sites (<50) started in the 1990s, most sites’ data records were concentrated in 2004–2020, with around 140 sites operating concurrently (Supplementary Figure S2). Figure 5 illustrates the temporal characteristics of selected flux data across AmeriFlux sites, highlighting a few long-running sites (red lines in left panels and time series in right panels). Most flux data show evident temporal variation at the sub-daily to daily and seasonal to annual scales, reflecting biological (e.g., phenology) and climatic regulation (Fig. 5a,c,e,g,i). Yet, distinct temporal variations were observed across sites depending on the temporal scales. For example, CH_4_ fluxes (FCH4) show weak to negligible seasonality at some but not all sites (Fig. 5i). And no consistent temporal variation was observed for all flux variables on weekly to monthly scales. With more than 100 sites now having decade-long records, it becomes feasible to explore the temporal characteristics at a longer scale. While some sites reveal weak variability near the quinquennial scale, we did not find a general pattern across sites.

## Discussion

### Network growth and data sharing

Since its onset, AMP has engaged with the AmeriFlux community, both the site teams and data users, through services centered on data, technique, and outreach. During this period, AMP supported and facilitated the growth of the AmeriFlux network, reflected in the rapid increase in registered sites, available data, and data usage (Figs. 1, 3, Supplementary Figure S2).

Since the network’s conception, data sharing has been a core tenet of AmeriFlux. AMP strives to maintain this practice, focusing on the dual goals of increasing the number of site teams contributing data and improving the quality and quantity of the data available. Key to this approach is semi-automation in the BASE data-processing pipeline, which has led to dramatic improvements in the breadth of QA/QC checks performed and the consistency of a high-quality BASE data product. Additionally, the BASE data-processing pipeline reduces the turnaround time that site teams receive feedback from 6–12 to 1–2 months, enabling more rapid data correction. While the QA/QC checks may present a hurdle for new site teams submitting their data for the first time, the independent data quality assessment by AMP is a key benefit of joining the network. And once the site teams became familiar with the QA/QC processes, the time from submission to publication was significantly reduced. Overall, the pipeline decreased the latency time from data collection to release. The addition of a CC-BY-4.0 data policy adopted by a majority of the network has significantly improved the findability, accessibility, interoperability, and reusability of the data.

### Synthesis and extended products

The AmeriFlux BASE data product’s life cycle continues after its release, further enabling and facilitating numerous data products and syntheses. For example, the FLUXNET data products—a gap-filled data product with value-added variables (i.e., partitioned gross primary productivity)—are part of global datasets used for model validation and benchmarking for decades^40,41^. In this regard, AMP collaborates with international partners like ICOS to develop the ONEFlux (Open Network-Enabled Flux) codes, fostering the creation of the FLUXNET2015 data product^42^. Furthermore, AMP is leveraging the high-quality standardized BASE data product as input to the ONEFlux codes to produce the next-generation FLUXNET data product for AmeriFlux sites. Additionally, the infrastructure and workflows developed for the BASE data-processing pipeline are being extended to produce the FLUXNET product (Fig. 2). As of 2022, AMP released the new AmeriFlux FLUXNET data product for 79 AmeriFlux sites. AMP anticipates continuing to release and update the AmeriFlux FLUXNET data products in coordination with other flux network partners^43^. The FLUXNET-CH4 community data product demonstrates another example of an extended product based on the BASE data product^44,45^. Among 81 sites included in the FLUXNET-CH4 data product, 45 are AmeriFlux sites that make their data available through the BASE data product.

AmeriFlux BASE data also facilitate syntheses that utilize data from multiple sites, a unique tool for scientific discovery. Recent examples include fundamental research^13,46–48^, model evaluation and benchmarking^49,50^, remote-sensing validation^51–54^, machine learning^55,56^, and science education^57^.

### Future direction of the data pipeline

The AmeriFlux BASE data-processing pipeline design considers the network’s unique aspects, such as distributed site teams, diverse instrumentation and processing routines, which distinguishes it from those implemented by other flux networks^30,31,58,59^. The data-processing pipeline incorporates many features (e.g., visualization, QA/QC report summaries, central communication tracking) to facilitate interactions with individual site teams. While the Format QA/QC assessment was fully automated earlier in the pipeline development, the Data QA/QC assessment remains a semi-automated process. The Data QA/QC module automatically generates statistics and figures, and AMP team members evaluate results and synthesize identified issues into a concise, readable, and actionable report. Full automation is challenging to achieve. For example, a single data issue can trigger warnings in multiple QA/QC checks. Thus, identifying and interpreting the root cause can be non-trivial. Without a concise report, the figures and statistics alone are difficult for data providers (particularly new site teams) to interpret and take appropriate action. At the same time, manual review by AMP is unsustainable, given the expected network and data-submission growth. Further development on fully automatic and self-interpretable Data QA/QC reports and training for site teams is in progress to further reduce the turnaround time and keep pace with the network growth and continuous data updates.

While most AmeriFlux sites’ data concentrated on about 60 common variables (e.g., fluxes, radiation, meteorology, soil, Fig. 4), research innovation has promoted the discussion of new variables and/or metadata. We partner with the AmeriFlux community members and other networks to develop new variables and their corresponding metadata and data check and processing routines. For example, to support the activities in the Year of Methane in 2018–2019, we worked with the Global Carbon Project, FLUXNET, and ICOS to add new aquatic variables (e.g., water temperature, dissolved oxygen) to the FP Standard. Most recently, the Year of Remote Sensing also facilitated the addition of new tower-based spectral variables, e.g., Near Infrared Vegetation Index^60^. The pipeline is designed to seamlessly support these new types of continuous measurements as they are added to the FP Standard. If new variables require additional quality assessments, the Data QA/QC module can be easily extended due to its modular design.

## Methods

### Data collection and processing at individual sites

AmeriFlux flux-met data’s life cycle begins with data collection at each field site using a suite of automated instruments. The instruments may vary from site to site but include eddy-covariance instruments (i.e., sonic anemometer, gas analyzer) and a selected set of meteorological, soil, and biological sensors. The data streams are recorded continuously (e.g., 10–20 Hz for flux measurements, 1-0.1 Hz or slower for others) by the data acquisition systems (e.g., logger, computer) and retrieved via physical visits or remote connection (e.g., cellular modem, radio transfer, Ethernet, satellite). Next, the site teams apply quality control and process the high-frequency data using selected software or in-house codes to produce flux-met data at a half-hourly or hourly resolution. Previous comparison studies showed that software selection generally led to marginal differences^29,61–63^ although the differences in the corrections implemented could also lead to systematic biases (e.g., spectral corrections function of air humidity^64^). Yet, the selection of corrections applied in the flux calculation (e.g., coordinate rotation, despiking, time lag optimization, spectral corrections), judged and augmented by individual researchers, can vary among sites based on sites’ characteristics (e.g., climate, canopy heights, tower structures, instrument types, and setup). Last, the data are checked and filtered by the site teams before uploading to the AmeriFlux website. Gap-filling is not required, but gap-filled variables can be provided in addition to non-filled ones.

### AmeriFlux BASE data-processing pipeline

The goal of the AmeriFlux BASE data-processing pipeline is to provide high-quality flux-met data in a standardized format that enables a broad range of Earth science research and educational activities. Our approach requires site teams to process high-frequency observations into half-hour or hourly fluxes (described above), prepare them in a standardized format (details below), and then submit these data to the AmeriFlux website. Upon submission, our semi-automated BASE processing pipeline is initiated and performs QA/QC checks (Fig. 2). If the submitted data pass the QA/QC checks, the resulting BASE data product is published, i.e., made publicly available on the AmeriFlux website. All data uploads are logged, all communications are tracked, and the data provenance is maintained.

The BASE processing pipeline consists of 3 modular components: Format QA/QC, Data QA/QC, and BASE Publish (Fig. 2). The automated portions of the pipeline are primarily written in Python (see Code Availability for the code repository). The pipeline logs the processing status of all data submissions and published BASE data products in a SQL database. All detected data issues and communication between the site team and AMP are recorded in information technology JIRA Service Management.

**Fig. 6:**
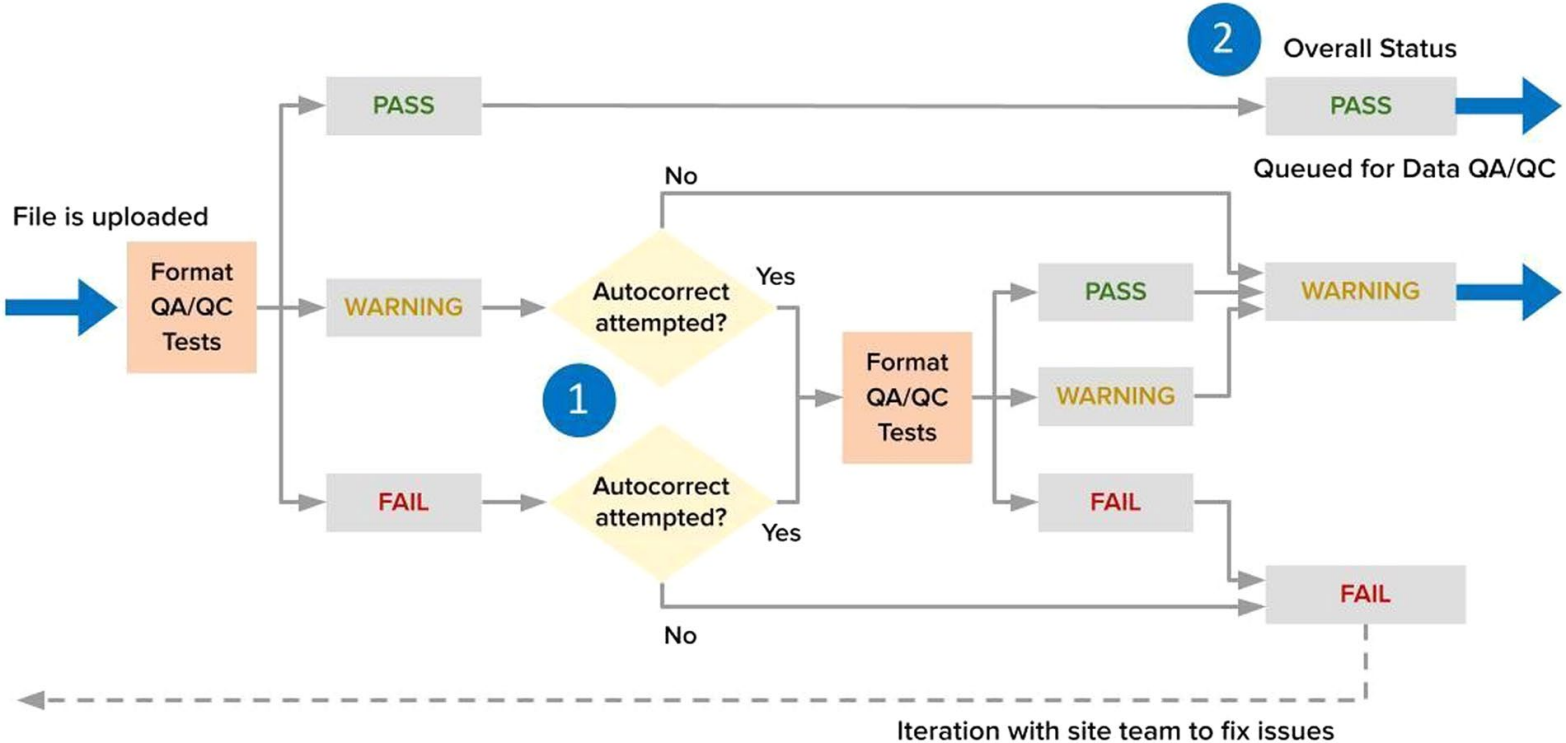
Format QA/QC workflow. Once a flux-met file is uploaded, the Format QA/QC module is automatically executed to assess format compliance with the required AmeriFlux FP-In format. A single autocorrection attempt is made if format issues are found (1 in the figure). An automated email is sent to the uploader that indicates the overall status (2), site team action, and links to Format QA/QC Reports detailing any format issues. The figure is adapted from Faybishenko *et al*.^70^.

The **Format QA/QC** module assesses compliance of submitted data files with the AmeriFlux FP-In (Flux Processing In) standardized format^65^. It makes one attempt to automatically correct minor issues if discovered (Fig. 6). The site teams receive a Format QA/QC report within a few hours after submission (Supplementary Figure S3). The FP-In format follows the timestamp, variable name, units, and data formatting conventions of the global FP (Flux Processing) format, namely a comma-delimited file with variables in columns at a timestep of half-hour or an hour in rows. The minimum variables required are the start and end timestamps and one carbon flux observation (FC or FCH4). However, most site teams also submit gas concentrations, gas and energy fluxes, basic meteorological observations (e.g., air temperature, wind speed and direction), and radiation observations. In requiring the FP-In format, the automated pipeline code can attempt fully automatic correction of various minor errors, including filling the skipped time intervals with the missing value designator −9999, fixing incorrect variable names, changing the file format to CSV, etc. Site teams can submit a site’s full data record, replacement data for previously submitted data, or new data that extend the site’s record.

The **Data QA/QC** module assesses the quality of flux-met data uploaded to AmeriFlux. It is a secondary data quality assessment that is independent of and complementary to the data quality checks performed by site teams prior to upload. The Data QA/QC follows a similar methodology to the FLUXNET2015 dataset^42,66^ but includes additional checks based on data user feedback (e.g., emails, workshops). Also, its design considers the long history of AmeriFlux data repositories and the diverse ecosystems and climates of AmeriFlux sites. For example, specific checks were developed to detect spurious trends and shifts in long-term records. Site-specific plausible ranges were constructed for each site to accommodate the wide range of climatic and ecosystem conditions. Last, the Data QA/QC uses data visualization and a ticket-tracking system (i.e., JIRA Service Management) to facilitate communication with site teams. Six Data QA/QC check modules are implemented currently: timestamp alignment, physical range, multivariate comparison, diurnal-seasonal pattern, USTAR filtering, and variable coverage (Table 2). Details and example figures of each module are provided in Supplementary Materials (Supplementary Text S1, Supplementary Figures S4-S17). AMP also hosts workshops and webinars for site teams to learn about the QA/QC (recordings available at https://ameriflux.lbl.gov/community/amp-webinar-series/).

**Table 1:** A list of the AmeriFlux site ID (316) used in the wavelet power spectra analyses.

AR-TF1	CR-SoC	US-CdM	US-GBT	US-Kon	US-Ne1	US-PHM	US-SRG	US-Whs	US-xKA
AR-TF2	MX-Aog	US-Ced	US-GLE	US-KPL	US-Ne2	US-Pnp	US-SRM	US-Wi0	US-xKZ
BR-CST	MX-PMm	US-CF1	US-Ha1	US-KS1	US-Ne3	US-Prr	US-Srr	US-Wi1	US-xLE
BR-Npw	MX-Tes	US-CF2	US-Ha2	US-KS2	US-NGB	US-PSH	US-SRS	US-Wi3	US-xMB
CA-ARB	PE-QFR	US-CF3	US-HB1	US-KS3	US-NGC	US-PSL	US-SSH	US-Wi4	US-xML
CA-ARF	PR-xGU	US-CF4	US-HB2	US-KUT	US-NMj	US-RGA	US-StJ	US-Wi5	US-xNG
CA-Ca1	PR-xLA	US-CMW	US-HB3	US-Lin	US-NR1	US-RGB	US-SuM	US-Wi6	US-xNQ
CA-Ca2	US-A03	US-Cop	US-HBK	US-LL1	US-NR3	US-RGo	US-SuS	US-Wi7	US-xNW
CA-Ca3	US-A10	US-CPk	US-Hn2	US-LL2	US-NR4	US-RGW	US-SuW	US-Wi8	US-xPU
CA-Cbo	US-A32	US-CRT	US-Hn3	US-LL3	US-Oho	US-Rls	US-Syv	US-Wi9	US-xRM
CA-Cha	US-A74	US-CS1	US-Ho1	US-Los	US-ONA	US-Rms	US-Ton	US-Wjs	US-xRN
CA-DB2	US-Act	US-CS2	US-Ho2	US-LS1	US-ORv	US-Ro1	US-TrB	US-Wkg	US-xSB
CA-DBB	US-Akn	US-CS3	US-Ho3	US-LS2	US-OWC	US-Ro2	US-Tw1	US-Wlr	US-xSC
CA-ER1	US-ALQ	US-CS4	US-HRA	US-Me2	US-PAS	US-Ro3	US-Tw2	US-WPT	US-xSE
CA-LP1	US-AR1	US-CS5	US-HRC	US-Me6	US-PFa	US-Ro4	US-Tw3	US-Wrc	US-xSJ
CA-MA1	US-AR2	US-DFC	US-Hsm	US-Men	US-PFb	US-Ro5	US-Tw4	US-xAB	US-xSL
CA-MA2	US-ARM	US-DFK	US-HWB	US-MH1	US-PFc	US-Ro6	US-Tw5	US-xAE	US-xSP
CA-MA3	US-ASH	US-Dia	US-ICh	US-MH2	US-PFd	US-Rpf	US-Twt	US-xBA	US-xSR
CA-Man	US-ASM	US-Dix	US-ICs	US-Mi1	US-PFe	US-Rwe	US-Uaf	US-xBL	US-xST
CA-Na1	US-Bar	US-Dk1	US-ICt	US-Mi2	US-PFg	US-Rwf	US-UC1	US-xBN	US-xTA
CA-Oas	US-Bi1	US-Dk2	US-Jo1	US-Mi3	US-PFh	US-Rws	US-UC2	US-xBR	US-xTE
CA-Obs	US-Bi2	US-Dk3	US-Jo2	US-Mj2	US-PFj	US-SdH	US-UiA	US-xCL	US-xTL
CA-SF1	US-Blo	US-DPW	US-JRn	US-MMS	US-PFk	US-Seg	US-UiB	US-xCP	US-xTR
CA-SF2	US-BMM	US-DS3	US-KFS	US-MOz	US-PFL	US-Ses	US-UiC	US-xDC	US-xUK
CA-SF3	US-Bo1	US-EDN	US-KL1	US-Mpj	US-PFm	US-Slt	US-UM3	US-xDJ	US-xUN
CA-TP1	US-Bo2	US-Elm	US-KL2	US-MtB	US-PFn	US-Snd	US-UMB	US-xDL	US-xWD
CA-TP2	US-BRG	US-EML	US-KL3	US-MVW	US-PFo	US-Sne	US-UMd	US-xDS	US-xWR
CA-TP3	US-Bsg	US-Esm	US-KLS	US-Myb	US-PFp	US-Snf	US-Var	US-xGR	US-xYE
CA-TP4	US-BZB	US-Fcr	US-KM1	US-NC1	US-PFq	US-SP1	US-Vcm	US-xHA	
CA-TPD	US-BZF	US-Fmf	US-KM2	US-NC2	US-PFr	US-SP2	US-Vcp	US-xHE	
CL-SDF	US-BZo	US-Fuf	US-KM3	US-NC3	US-PFs	US-SP3	US-Vcs	US-xJE	
CL-SDP	US-BZS	US-Fwf	US-KM4	US-NC4	US-PFt	US-SRC	US-WCr	US-xJR	

See Supplementary Table S2 for each site’s data years and citations.

**Table 2:** Summary of the target issues for each Data QA/QC module.

Module	Issue category	Issue
Timestamp Alignment	Wrong timestamp specification	• Misspecified beginning or ending timestamps • Timestamps not matched with time zone specification • Use of daylight saving time • Data streams not synchronized
	Radiation measurement issue	• Tilted radiation sensor • Shaded radiation measurements • Higher than expected radiation readings
Physical Range	Plausibility check	• Excessive outlier (i.e., out-of-range) points • Percentage-ratio check (i.e., percentages provided as ratios)
	Variability check	• Trend • Step change • Repeating patterns or filled constants • Measurement or processing cut-off • Other unrecognized patterns
Multivariate Comparison	Short-term mismatch	• Outlier (sporadically erroneous data) • Short-term mismatch (erroneous data for a specific period) • Shaded radiation (periodically erroneous data)
	Unexpected relationship	• Variables not synchronized in time • Derived one from another (perfectly fit)
	Change of slope	• Trend (systematic change in the regression slope) • Step change in full range (change in the regression slope)
Diurnal-Seasonal Pattern	Misalignment between median diurnal composite	• Change of the sign convention • Shift in timestamps
	Unexpected data ranges	• Physically unlikely values • Excessive outlier • Step change in the full range
USTAR Filtering	FC-USTAR filtering	• Filtered FC by USTAR threshold • Filtered USTAR
Variable Coverage	Unexpected variable coverage	• Long data gaps • All empty columns • Missing mandatory variables • Mismatched or inconsistent variable naming

Details of each module are explained in Supplementary Text S1. Example figures are provided in Supplementary Figures S4-S17.

**Fig. 7:**
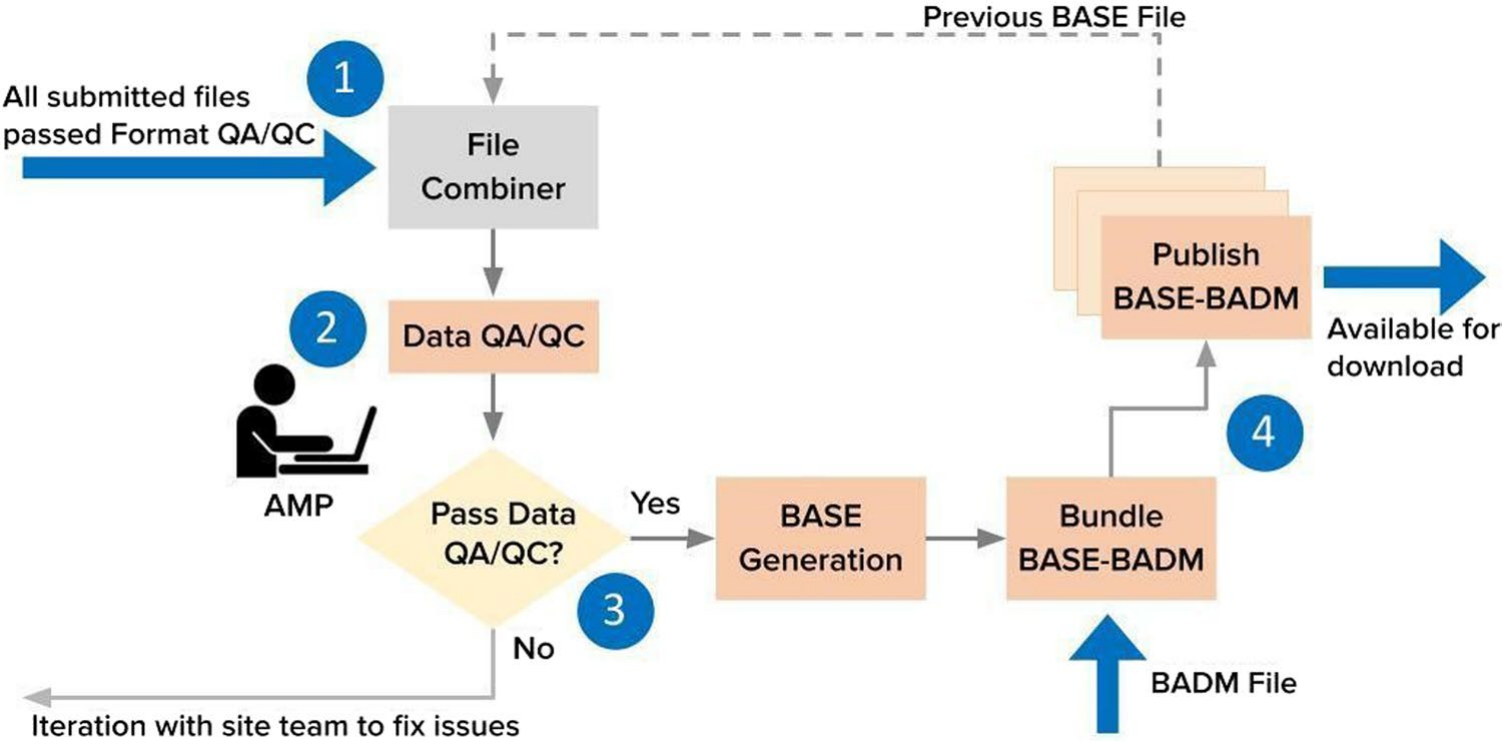
Data QA/QC workflow. After passing Format QA/QC, uploaded files are combined with, if any, previously published BASE files (1). The automated Data QA/QC codes generate statistics and figures that AMP reviews (2). If the data trigger any potential issues, AMP notifies the site team of corrections needed (3). Otherwise, the data are queued for BASE generation and bundled with BADM for publication (4).

Once passing Format QA/QC, the uploaded files are combined with a site’s previously published BASE data product to form a complete data record (Fig. 7). Data QA/QC modules are executed and automatically generate figures and summary statistics (e.g., Supplementary Figure S18). The module execution time is typically within a few hours for a site’s data. Then, AMP conducts Data QA/QC reviews of sites in batches ranging from weekly to monthly and synthesizes the identified issues into a concise, actionable report (e.g., Supplementary Figure S19). While varying among cases, the average time for Data QA/QC review is typically less than an hour for each site. The report also explains the background of Data QA/QC and provides links to all summary statistics and figures generated. If there are identified issues, AMP notifies the site team of corrections needed. Otherwise, the data are queued for BASE data publication.

The **BASE Publish** module occurs after data pass the Data QA/QC, typically in batches once every 1–2 months for both new sites publishing for the first time and returning sites updating data. AMP formats the flux-met data in the FP Standard format, bundles them with Biological, Ancillary, Disturbance, and Metadata (BADM, details below), and versions the bundled data. In addition, the module obtains Digital Object Identifier (DOI) for new data and updates metadata for existing DOIs before making the BASE data product available on the AmeriFlux website. The BASE data product is organized by sites, with one zipped file containing both BASE and BADM data of an AmeriFlux site. Details of the file format and structure are provided in Supplementary Text S2.

In addition to data search and download access, the AmeriFlux website also supports a suite of web-based features for showing each site’s general information, data citation, download logs, images, publications, and related data (e.g., prevailing wind visualizations). Each site with published BASE data that has been assigned a DOI can edit its contributor lists. Last, external links to the sites’ cut-outs of remote-sensing and gridded products, such as MODIS, VIIRS, ECOSTRESS, and Daymet, are also provided through collaborative agreements with Distributed Active Archive Center (DAAC) at ORNL. See Supplementary Text S3 for a quick guide for BASE data use.

### BASE data policy

Starting in Fall 2021, AMP worked with AmeriFlux site teams to adopt the new AmeriFlux CC-BY-4.0 Data Use License, which allows data to be shared under the widely-used Creative Commons BY 4.0 license (CC-BY-4.0). As of the end of 2022, 406 AmeriFlux sites (~69% of registered sites) have adopted the CC-BY-4.0 Data Use License. Among 444 sites with BASE data, 344 sites (~77%) are under the CC-BY-4.0 license. The CC-BY-4.0 license makes AmeriFlux data more compatible with other flux networks (e.g., ICOS, OzFlux, and NEON) and more consistent with the FAIR (Findable, Accessible, Interoperable, and Reusable) principle of accessibility, which is now widely encouraged or required by many journal publishers and funding agencies.

### Relevant metadata supporting base data

Biological, Ancillary, Disturbance, and Metadata (BADM) are non-continuous information that characterizes a site and complements the BASE flux-met data. BADM includes general site descriptions, metadata about the instruments, maintenance and disturbance events, and biological and ecological data^67^. See the AmeriFlux website for a complete and updated list of all BADM groups and variables^68^.

To support AmeriFlux BASE data use, AMP developed and released multiple new BADM sets, including the Measurement Height data, which provides information on BASE data measurement heights/depths and instrument models. The Measurement Height information is provided directly by the site teams or pulled by AMP from historical records and is updated in conjunction with the BASE Publish schedule.

### Supplementary information

Supplementary Information

## Data Availability

All data discussed in this paper are publicly available at AmeriFlux (https://ameriflux.lbl.gov/) as the BASE and BADM data products. The published data are licensed under the AmeriFlux CC-BY-4.0 or the AmeriFlux Legacy Use Data License based on the site team’s selection. Additional data will be published as they are submitted and pass the QA/QC process described in this paper.

## Code availability

The core Python-based BASE data-processing pipeline code is available under a modified BSD license at https://github.com/AMF-FLX/AMF-BASE-QAQC. The R-based code for generating the article’s figures is available at 10.5281/zenodo.8250754.
