# Supplementary material for: AmeriFlux BASE data pipeline to support network growth and data sharing
Source: Sci Data. 2023 Sep 11;10:614. doi: 10.1038/s41597-023-02531-2 (PMC10495345; doi:10.1038/s41597-023-02531-2)
Supplement: Supplementary file 1 — Supplementary Information [file 41597_2023_2531_MOESM1_ESM.pdf]

Title
AmeriFlux BASE data pipeline to support network growth and data sharing

Authors
Housen Chu[1], Danielle S. Christianson[2], You-Wei Cheah[2], Gilberto Pastorello[2], Fianna O'Brien[2], Joshua Geden[2], Sy-Toan Ngo[2], Rachel Hollowgrass[3], Karla Leibowitz[4], Norman F. Beekwilder[5], Megha Sandesh[2], Sigrid Dengel[1], Stephen W. Chan[1], André Santos[1], Kyle Delwiche[3], Koong Yi[1], Christin Buechner[1], Dennis Baldocchi[3], Dario Papale[6,7], Trevor F. Keenan[1,3], Sébastien C. Biraud[1], Deborah A. Agarwal[2], and Margaret S. Torn[1,8]

Affiliations

1. Climate & Ecosystem Sciences Division, Lawrence Berkeley National Laboratory, Berkeley, CA, 94720, USA.
2. Scientific Data Division, Lawrence Berkeley National Laboratory, Berkeley, CA, 94720, USA.
3. Department of Environmental Science, Policy, and Management, University of California Berkeley, Berkeley, CA, 94720, USA.
4. HyperArts, Inc, Oakland, CA, 94607, USA.
5. Department of Computer Science, University of Virginia, Charlottesville, VA 22903, USA
6. DIBAF, University of Tuscia, Viterbo, 01100, Italy.
7. Euro-Mediterranean Center on Climate Change CMCC IAFES, Viterbo, 01100, Italy
8. Energy and Resources Group, University of California Berkeley, Berkeley, CA, 94720, USA.

Corresponding author(s): Housen Chu (hchu@lbl.gov)

Table of Content
Supplementary Text S1. Technical Details on Data QA/QC Modules 2
Supplementary Text S2. Data File and Content 5
Supplementary Text S3. A Quick Guide for BASE Data Use 7
References 10
Supplementary Table S1. A list of selected variable names in the BASE data product 11
Supplementary Table S2. The list of sites and data citations used in wavelet analyses 14
Supplementary Figure S1. Distribution of AmeriFlux sites by temperature and precipitation 38
Supplementary Figure S2. AmeriFlux BASE data availability by the time periods published 39
Supplementary Figure S3. An example of the Format QA/QC report 40
Supplementary Figure S4-S17. Example figures of the Data QA/QC modules 41-54
Supplementary Figure S18. An example of the summary statistics 55
Supplementary Figure S19. An example of the Data QA/QC report 56

Supplementary Text S1. Technical Details on Data QA/QC Modules

The Data QA/QC process assesses the data's timestamp alignments, outliers, ranges, variabilities, units, and sign conventions. It is a secondary data quality assessment that is independent of and complementary to the data quality checks performed by site teams prior to submission. The Data QA/QC follows a similar methodology for quality-checking and processing as the FLUXNET2015 dataset[1,2] but includes additional checks based on data user feedback. Currently, six QA/QC modules are implemented. Details for individual modules are summarized in the following sections.

1.1. Timestamp Alignment Module

The timestamp alignment module examines the alignment between the measured incoming radiation, e.g., photosynthetically active radiation (PPFD_IN), shortwave radiation (SW_IN), and the calculated potential incoming radiation at the top of the atmosphere (SW_IN_POT). The module is used to identify the following issues:

- Wrong timestamp specification
  - Misspecified beginning or ending timestamps
  - Timestamps not matched with time zone specification
  - Use of daylight saving time
  - Data streams not synchronized
- Radiation measurement issue
  - Tilted radiation sensor
  - Shaded radiation measurements
  - Higher than expected radiation readings

For each site-year, (half-)hourly SW_IN_POT is calculated based on a site's geolocation and time zone provided in the BADM site general information. Then, the SW_IN_POT, SW_IN, and PPFD_IN data are aggregated into a "maximum diurnal composite" for each of the 15-day non-overlapping windows (Supplementary Figure S4). PPFD_IN data are converted into an energy unit (W m^{-2}) by an approximate coefficient of 0.5 J μmol^{-1}. The calculation of maximum diurnal composite eliminates periods with cloudy conditions and allows the alignment analysis on mostly clear-sky conditions. The module expects that the diurnal composites align between SW_IN_POT, SW_IN, and PPFD_IN indicated by the cross-correlation assessment and that SW_IN and PPFD_IN do not exceed SW_IN_POT in the morning or afternoon hours. Misalignment between measured and calculated radiation indicates possible issues of timestamp alignment or radiation measurements (Supplementary Figure S5).

1.2. Physical Range Module

The physical range module examines the full range of the target variable. The module assesses all variables. The module can be used to identify the following issues:

- Plausibility check
  - Outlier (i.e., out-of-range) points
  - Percentage-ratio check (i.e., percentages provided as ratios)

- Variability check
    - Trend
    - Step change
    - Repeating patterns or Filled constants
    - Measurement or Processing cut-off
    - Other unrecognized patterns

For each variable, the accepted range is defined based on its physically plausible range, for example, 0-100 for percentage variables, and the distribution of the published data across the AmeriFlux sites. A ±5% buffer is applied to account for possible edge values near the lower and upper bounds, commonly observed for radiation variables, relative humidity (RH), and snow depth. A data point is soft-flagged outside the expected physical range but within the buffer range (±5% of the physical range) and hard-flagged outside the buffer range. The percentage of flagged points each year and in the entire record determines if a variable has excessive out-of-range data points (Supplementary Figures S6-S7). The module also assesses if a variable with units in percent (e.g., RH, soil water content (SWC), 0-100) is provided in ratios (i.e., 0-1).

While the module is mainly designed to detect out-of-range data points, additional issues, like trends and step-changes, may be identified through manual inspection of the multi-year figures. For sites with previously published BASE datasets, the Diurnal-Seasonal Pattern Module may provide additional quantitative information on the potential changes of full ranges (e.g., trends, step change, cut-off) (Supplementary Text S1.4). In addition, the Multivariate Comparison Module also assesses the potential changes of full ranges over years if a pair of associated variables (e.g., SW_IN, PPFD_IN) are both measured (see Supplementary Text S1.3).

1.3. Multivariate Comparison Module

The multivariate comparison module examines the relationship between a pair of associated variables that measure different but physically related quantities, e.g., SW_IN vs. PPFD_IN, friction velocity (USTAR) vs. wind speed (WS), air temperature (TA) vs. sonic temperature (T_SONIC). In addition, the module also compares variables that measure the same quantity at different locations or using different sensors, e.g., vertical profile and co-located sensors. The module assumes a consistent or predictable relationship between associated variables over time and uses that to identify the following potential issues:

- Short-term mismatch
    - Outlier (sporadically erroneous data)
    - Short-term mismatch (erroneous data for a specific period)
    - Shaded radiation (periodically erroneous data)
- Unexpected relationship
    - Variables not synchronized in time (excessive scattering)
    - Derived one from another (perfectly fit)
- Change of slope
    - Trend (systematic change in the regression slope)
    - Step change in full range (change in the regression slope)

The module first fits a linear regression (model II) between the two targeted variables over each year and the full data record (Supplementary Figure S8). The module then calculates each point's orthogonal distance to the regression line. Data points with relatively large deviations from the regression line are flagged as possible outliers. The percentages of flagged points in each year and the entire record determine if a variable has excessive out-of-range data points (Supplementary Figure S9).

For variables provided for more than one year, the module also examines the year-to-year changes in the annual regression slopes. Potentially, any change in the regression slopes over the years could indicate a trend or a step-change in the full range of a variable (Supplementary Figures S10-S11).

1.4. Diurnal-Seasonal Pattern Module

The module examines the diurnal-seasonal pattern of a target variable against the historical records at a site and determines if the (newly submitted) data are within the expected range. In particular, the module relies on the pronounced temporal variations at the diurnal and seasonal scales of most micrometeorological variables. This module is considered a companion to the Physical Range Module and uses more constrained expected ranges. The check only performs at sites that have previously published BASE data versions and have data records for at least three years. The check is used to identify the following issues:

- Misalignment between median diurnal composite
    - Change of the sign convention
    - Shift in timestamps
- Unexpected data ranges
    - Physically unlikely values
    - Outlier
    - Step change in the full range

The module compares the diurnal-seasonal pattern of the newly submitted data against the historical records at the site (Supplementary Figure S12). The historical ranges (i.e., 2.5%, 25%, 50%, 75%, and 97.5%) were generated for each variable based on the last version of published BASE data at the site. Both the newly submitted data and historical ranges are organized by the month of the year (i.e., 12 windows per year) and (half-)hour of the day in each month (i.e., 48 steps for a half-hourly resolution). The newly submitted data are also aggregated into a "median diurnal composite" for each monthly window. The module expects the median diurnal composites to align in time between the newly submitted data and the historical records (cross-correlation). For example, a negative cross-correlation suggests that the submitted data may have an opposite sign convention.

The module also checks the percentages of newly submitted data within the 25%-75% and 2.5%-97.5% ranges of the historical ranges. If the new data have fewer than the expected data percentages within the corresponding ranges, then the module returns a WARNING or FAIL result. Supplementary Figure S13 shows an example year with relatively higher LW_OUT readings (more gray points beyond the historical ranges), indicating a possible shift of the full range.

1.5. USTAR Filtering Module

The USTAR (friction velocity) filtering module examines whether the CO_2 flux (FC) is filtered using USTAR thresholds. Flux data submitted to AmeriFlux should not be USTAR-filtered, as the subsequent ONEFlux processing step implements a standard procedure in determining the USTAR thresholds and filtering FC data. The module is used to identify the following issues:

- Filtered FC by USTAR threshold
- Filtered USTAR

For each pair of USTAR and FC (i.e., values for the same year), the module finds the lower bound of USTAR when the concurrent FC is not missing and from all USTAR data in a year (Supplementary Figures S14-S15), respectively. The module expects these two lower bounds to match and be close to the expected lower range of USTAR.

1.6. Variable Coverage Module

The variable coverage module examines the presence and coverage of all variables submitted by the site. The generated figure provides a quick overview of the submitted variables and their data coverage for each year of the entire record (Supplementary Figures S16-S17). The figure can be used to examine whether the data in certain years are entirely missing (e.g., inactive years), certain variables are missing for specific periods (e.g., not measured, unsubmitted), or certain variables are entirely missing (e.g., all empty columns). Additionally, mistakes in variable names can be easily identified, especially if data are submitted in separate periods. For long-running and heavily-instrumented sites, the figure can be used to verify the presence and continuity of variables across the entire record.

Supplementary Text S2. Data File and Content

The AmeriFlux BASE data product is organized by sites, with one zipped file containing the BASE data and BADM for an AmeriFlux site. The BASE data file is provided in plain ASCII text using a comma-separated values (CSV) format, using the following file naming convention:

AMF_<SITE_ID>_BASE_<RESOLUTION>_<VERSION>.csv

where <SITE_ID> is a unique site identification code in the format of CC-Sss (e.g., US-Ha1), <RESOLUTION> indicates the temporal resolutions of the data (i.e., HR for hourly and HH for half-hourly). <VERSION> contains the version numbers of data and processing codes separated by a dash (e.g., 2-5, 1-4). The data version corresponds to the site team's data submission. It increases incrementally when the BASE data are updated due to changes in data submitted from the site teams, e.g., additional years, variables, or replacement. The processing version corresponds to the version of the data processing pipeline used to generate the BASE data product. Changes to the data processing pipeline are documented in the AmeriFlux Data Change Log (https://ameriflux.lbl.gov/data/data-change-log/).

2.1. Data File Header and Variable Name

The header of the BASE data file is composed of two lines starting with a hash character (#). The first line specifies the <SITE_ID> while the second line specifies the <VERSION> as described above.

The rest of the file follows the global FP (Flux Processing) Standard[3]. The first row after the headers provides the variable labels. Variable labels consist of the variable root names (Supplementary Table S1) and suffix qualifiers that provide additional information about the variable. Most common suffixes include the gap-filled suffix (_PI_F), positional suffix (e.g., _1_1_1), and layer-aggregated suffix (e.g., _1). Timestamps are specified in truncated ISO format with both the start and end timestamps of the averaging period.

The number and order of columns in these files are not guaranteed to be uniform, except that timestamps (i.e., TIMESTAMP_START, TIMESTAMP_END) are always located in the first two columns. TIMESTAMP_START and TIMESTAMP_END refer to the start and end of the reporting interval. Time is reported in local standard time (i.e., without Daylight Saving Time). Data for all days in a leap year are reported. The -9999 value indicates missing data records.

2.2. Variable Qualifier

Qualifiers are suffixes appended to variable root names that provide additional information about the variable. A visual guide to variable qualifiers is available on the AmeriFlux website[4].

- _PI (Provided by PI/tower team): _PI indicates a variable that has been QA/QC filtered, spatially aggregated, or calculated by the tower team.
- _PI_F (Gap-filled variable): _PI_F indicates that the variable has been gap-filled by the tower team.
- _<H>_<V>_<R> (Position qualifier): The three components of the qualifier are indices that indicate an observation's spatial position. In other words, the indices describe the position of a sensor relative to other sensors that measure the same variable at a site, i.e., Horizontal position (_<H>), Vertical position (_<V>), Replicate (_<R>). The letters H, V, and R are to be replaced with integer values to represent, e.g., TS_1_1_1, TS_1_1_2, TS_1_2_1
- _PI_<H>_<V>_A (Aggregation of replicates): If replicates can be aggregated, they are averaged, and the result is reported with the Replicate index of the _<H>_<V>_<R> position qualifier replaced with the letter A. _PI means the variable is aggregated by the tower team. Continuing the example above, if TS_1_1_1 and TS_1_1_2 can be averaged, the result will be named TS_PI_1_1_A.
- _PI_<#> (Aggregation layer index): Variables with the same root name and the same height/depth but different horizontal positions can be aggregated. This aggregation across a horizontal plane represents the footprint at a given layer. _PI means the variable is aggregated by the tower team. The <#> qualifier is replaced by a numerical index indicating the layer's relative height/depth position, e.g., TS_PI_1

Supplementary Text S3. A Quick Guide for BASE Data Use

The AmeriFlux BASE data product contains format-standardized and quality-checked data for continuous flux, meteorological, and soil variables collected at AmeriFlux sites. The product supports > 140 variables and can accommodate observations from replications and multiple vertical and horizontal locations. While gap-filling is not required, gap-filled variables can be provided along with non-filled variables. The

BASE data product accommodates various variable types, aggregations (e.g., single sensor, replicate-averaged, layer-aggregated), and processing levels (e.g., gap-filled, non-filled, originally-measured, derived). Thus, the BASE data product differs from other flux datasets like the FLUXNET products[2], which only include site-representative variables and fewer variable types. AmeriFlux data policy has evolved over the years, including the Legacy Data Policy and the latest CC-BY-4.0 Data License, which is now adopted by ~75% of AmeriFlux sites. Data users must understand the policies and follow the guidelines when using AmeriFlux data. Last, the AmeriFlux website supports many features to facilitate the use and interpretation of BASE data, e.g., site search, site sets, customized map, bibliography tool, and quick plot. The 'amerifluxr' package also provides an R programmatic interface for querying, downloading, and handling AmeriFlux data and metadata[5]. We provide a quick but non-exclusive guide for BASE data use below.

- The BASE data product is organized by sites, i.e., a data package per site. Typically, data query begins with site search and down selection, using
    - The Site Search webpage (https://ameriflux.lbl.gov/sites/site-search/), where users can filter sites by site characteristics and/or data variables and years.
    - The Data Availability webpage (https://ameriflux.lbl.gov/data/data-availability/), where sites and years of available BASE data are visualized and can be downloaded as a CSV.
    - The 'amerifluxr' workflow in the R language (https://chuhousen.github.io/amerifluxr/articles/site_selection.html).
- Once a site list is decided, download the data through the website data portal (https://ameriflux.lbl.gov/data/download-data/) or via the 'amerifluxr' interface (https://chuhousen.github.io/amerifluxr/articles/data_import.html). Users will need an AmeriFlux account.
- The Site Sets (https://ameriflux.lbl.gov/sites/site-sets/) feature on the AmeriFlux website allows users to save a list of target sites and download their data, site general information, team members' contact, and data DOI. An AmeriFlux account is required.
- Once downloaded, the files are zipped files organized by individual sites. Each zip file contains a site's BASE data file (CSV format, Supplementary Text S2) and a BADM data file (Excel XLSX format, BADM Data Product webpage (https://ameriflux.lbl.gov/data/aboutdata/badm-data-product/)).
- The BASE product is time series data reported at regular intervals, half-hourly or hourly, for a certain period. TIMESTAMP_START and TIMESTAMP_END columns denote each interval's starting and ending time (i.e., YYYYMMDDHHMM). All timestamps are local standard time, and a site's time zone is specified in the BADM product (i.e., UTC_OFFSET).
- All other variables follow the format of <root name>_<qualifier>, e.g., FC_1, CO2_1_1_1. Root names indicate the fundamental quantities measured or calculated. See Supplementary Table S1 or the Data Variables webpage (https://ameriflux.lbl.gov/data/aboutdata/data-variables/) for a list of root names and their units and descriptions. Qualifiers are suffixes that provide additional information (e.g., gap-filling, position) and can be omitted if only one variable is provided for a site (see Supplementary Text S2.2 for qualifier details).

- While the BASE data product is quality-checked, the data may not be outlier-free. Users are advised to filter the data as needed (e.g., plausible range, quality flags (e.g., FC_SSITC_TEST)). In particular, CO_2 flux (FC) and all other flux variables are not filtered using friction velocity (USTAR) thresholds. Users are advised to perform USTAR-filtering before using the FC data.
- All flux variables are reported as turbulent fluxes, i.e., not corrected for storage changes. For CO_2, it is recommended to use net ecosystem exchange (NEE), which accounts for FC and the storage change of CO_2 within the canopy (SC), particularly in tall-vegetation sites. If not provided, users should calculate NEE from FC and SC (or CO_2 measurements if SC is not provided).
- The Measurement Height data product contains the essential height/depth and instrument model information. It could be critical to understanding the BASE data, particularly when a variable is measured at multiple locations (e.g., TS_1_1_1, TS_1_2_1).
- BADM describes and complements the BASE data product. BADM include general site description, metadata about the sensors and their setup, maintenance and disturbance events, and biological and ecological data that characterize a site's ecosystem. Read the BADM webpage for more information (https://ameriflux.lbl.gov/data/aboutdata/badm/). A BADM parser is available as a Jupyter notebook at https://github.com/AMF-FLX/AMF-UserTools[6]. Alternatively, the 'amerifluxr' package supports parsing the BADM product.
- Data users should review the requirements of the AmeriFlux data use policies before using AmeriFlux data. The data use policy is available for download as a pdf with the requested data on the AmeriFlux webpage portal at the time of download or via the Data Policy website (https://ameriflux.lbl.gov/data/data-policy/)[7]. Below are tips for implementing the policies. Please review the full data policy before using AmeriFlux data:
    - Using AmeriFlux data requires citation to the site's data product. The data DOI can be obtained from a CSV file included in the download links on the AmeriFlux webpage data portal, the sites BADM (i.e., DOI group) bundled in the zip file, or downloaded via the Site Sets website feature.
    - If using data shared under Legacy Data Policy for publication, data users are required to contact data contributors directly so that they have the opportunity to contribute substantively and become co-authors. The site teams' contact information can be obtained from a CSV file included in the download links on the AmeriFlux webpage data portal, the sites BADM (i.e., TEAM_MEMBER group) bundled in the zip file, or downloaded via the Site Sets website feature.
- Over the years, the flux research communities have developed many software and packages for post-processing flux and meteorological data. Those include but are limited to gap-filling, partitioning (e.g., gross primary production/ecosystem respiration, transpiration/evaporation), USTAR-filtering, and footprint analyses. Data users can utilize those for further processing the BASE data for research needs. See a list of software and packages at the Resources webpage (https://ameriflux.lbl.gov/resources/resource-list/tools-and-software-for-flux-scientists/flux-data-post-processing-and-qa-qc/)

References

1. Pastorello, G. *et al.* Observational Data Patterns for Time Series Data Quality Assessment. 2*014 IEEE 10th International Conference on e-Science.* Sao Paulo, Brazil, 2014, pp 271-278.
2. Pastorello, G. *et al.* The FLUXNET2015 dataset and the ONEFlux processing pipeline for eddy covariance data. *Scientific Data* **7**, 225 (2020).
3. AmeriFlux Management Project. *Data Variable*. https://ameriflux.lbl.gov/data/aboutdata/data-variables/ (2015).
4. AmeriFlux Management Project. *Data variable qualifier examples*. https://ameriflux.lbl.gov/data/data-variable-qualifier-examples/ (2018).
5. Chu, H. & Hufkens, K. amerifluxr v1.0.0. *R-CRAN* (2021). doi:10.11578/DC.20210730.2. https://CRAN.R-project.org/package=amerifluxr
6. Agarwal, D. A. AMF-UserTools: Tools created to help users of AmeriFlux data to use the data. *GitHub* (2022). https://github.com/AMF-FLX/AMF-UserTools
7. AmeriFlux Management Project. *Data policy*. https://ameriflux.lbl.gov/data/data-policy/ (2014).
8. Harris, I., Osborn, T. J., Jones, P. & Lister, D. Version 4 of the CRU TS monthly high-resolution gridded multivariate climate dataset. *Scientific Data* **7**, 109 (2020).

Supplementary Tables

Supplementary Table S1 A list of selected variable names (i.e., root name, Supplementary Text S2) in the BASE data product. The list contains variables that are most commonly (> 50 sites) submitted and it is a subset of variables that are supported by the Flux Processing (FP) Standard[3]. See the web page (https://ameriflux.lbl.gov/data/aboutdata/data-variables/) for a complete list of variables.

Variable	Description	Units
TIMESTAMP_START	ISO timestamp start of averaging period (up to a 12-digit integer as specified by the data's temporal resolution)	YYYYMMDDHHMM
TIMESTAMP_END	ISO timestamp end of averaging period (up to a 12-digit integer as specified by the data's temporal resolution)	YYYYMMDDHHMM
T_CANOPY	Temperature of the canopy and/or surface underneath the sensor	deg C
FETCH_90	Distance at which cross-wind integrated footprint cumulative probability is 90%	m
FETCH_70	Distance at which cross-wind integrated footprint cumulative probability is 70%	m
FETCH_MAX	Distance at which footprint contribution is maximum	m
CH4	Methane (CH4) mole fraction in wet air	nmolCH4 mol-1
CO2	Carbon Dioxide (CO2) mole fraction in wet air	µmolCO2 mol-1
FC	Carbon Dioxide (CO2) turbulent flux (no storage correction)	µmolCO2 m-2 s-1
FCH4	Methane (CH4) turbulent flux (no storage correction)	nmolCH4 m-2 s-1
H2O	Water (H2O) vapor in mole fraction of wet air	mmolH2O mol-1
SC	Carbon Dioxide (CO2) storage flux	µmolCO2 m-2 s-1
G	Soil heat flux	W m-2
H	Sensible heat turbulent flux (no storage correction)	W m-2
LE	Latent heat turbulent flux (no storage correction)	W m-2
SH	Sensible heat (H) storage flux	W m-2
SLE	Latent heat (LE) storage flux	W m-2
PA	Atmospheric pressure	kPa
RH	Relative humidity, range 0-100	%
TA	Air temperature	deg C

VPD	Vapor Pressure Deficit	hPa
T_SONIC	Sonic temperature	deg C
T_SONIC_SIGMA	Standard deviation of sonic temperature	deg C
P	Precipitation	mm
P_RAIN	Rainfall	mm
ALB	Albedo, range 0-100	%
LW_IN	Longwave radiation, incoming	W m-2
LW_OUT	Longwave radiation, outgoing	W m-2
NETRAD	Net radiation	W m-2
PPFD_IN	Photosynthetic photon flux density, incoming	µmolPhoton m-2 s-1
PPFD_OUT	Photosynthetic photon flux density, outgoing	µmolPhoton m-2 s-1
PPFD_BC_IN	Photosynthetic photon flux density, below canopy incoming	µmolPhoton m-2 s-1
SW_DIF	Shortwave radiation, diffuse incoming	W m-2
SW_IN	Shortwave radiation, incoming	W m-2
SW_OUT	Shortwave radiation, outgoing	W m-2
SWC	Soil water content (volumetric), range 0-100	%
TS	Soil temperature	deg C
MO_LENGTH	Monin-Obukhov length	m
TAU	Momentum flux	kg m-1 s-2
U_SIGMA	Standard deviation of velocity fluctuations (towards main-wind direction after coordinates rotation)	m s-1
USTAR	Friction velocity	m s-1
V_SIGMA	Standard deviation of lateral velocity fluctuations (cross main-wind direction after coordinates rotation)	m s-1
W_SIGMA	Standard deviation of vertical velocity fluctuations	m s-1
WD	Wind direction	Decimal degrees
WS	Wind speed	m s-1
WS_MAX	Maximum WS in the averaging period	m s-1
ZL	Monin-Obukhov Stability parameter	nondimensional
GPP	Gross Primary Productivity	µmolCO2 m-2 s-1

NEE	Net Ecosystem Exchange	µmolCO2 m-2 s-1
RECO	Ecosystem Respiration	µmolCO2 m-2 s-1
FC_SSITC_TEST	Results of the quality flagging for FC according to Foken et al 2004, based on a combination of Steady State and Integral Turbulence Characteristics tests by Foken and Wichura (1996) (i.e., 0, 1, 2)	nondimensional
H_SSITC_TEST	Quality flagging for H similar to FC_SSITC_TEST	nondimensional
LE_SSITC_TEST	Quality flagging for LE similar to FC_SSITC_TEST	nondimensional
TAU_SSITC_TEST	Quality flagging for TAU similar to FC_SSITC_TEST	nondimensional

**Supplementary Table S2** The list of sites and data citations used in wavelet analyses (Figure 5).		
Site ID.	Year	Data Citation
AR-TF1	2016-2018	Lars Kutzbach (2021), AmeriFlux BASE AR-TF1 Rio Moat bog, Ver. 2-5, AmeriFlux AMP, (Dataset). https://doi.org/10.17190/AMF/1543389
AR-TF2	2016-2018	Lars Kutzbach (2019), AmeriFlux BASE AR-TF2 Rio Pipo bog, Ver. 1-5, AmeriFlux AMP, (Dataset). https://doi.org/10.17190/AMF/1543388
BR-CST	2014-2015	Antonio Antonino (2019), AmeriFlux BASE BR-CST Caatinga Serra Talhada, Ver. 1-5, AmeriFlux AMP, (Dataset). https://doi.org/10.17190/AMF/1562386
BR-Npw	2013-2017	George Vourlitis, Higo Dalmagro, Joseì de S. Nogueira, Mark Johnson, Paulo Arruda (2019), AmeriFlux BASE BR-Npw Northern Pantanal Wetland, Ver. 1-5, AmeriFlux AMP, (Dataset). https://doi.org/10.17190/AMF/1579716
CA-ARB	2011-2015	Aaron Todd, Elyn Humphreys (2018), AmeriFlux BASE CA-ARB Attawapiskat River Bog, Ver. 1-5, AmeriFlux AMP, (Dataset). https://doi.org/10.17190/AMF/1480319
CA-ARF	2011-2015	Aaron Todd, Elyn Humphreys (2018), AmeriFlux BASE CA-ARF Attawapiskat River Fen, Ver. 1-5, AmeriFlux AMP, (Dataset). https://doi.org/10.17190/AMF/1480318
CA-Ca1	1996-2010	T. Andrew Black (2018), AmeriFlux BASE CA-Ca1 British Columbia - 1949 Douglas-fir stand, Ver. 1-5, AmeriFlux AMP, (Dataset). https://doi.org/10.17190/AMF/1480300
CA-Ca2	1999-2010	T. Andrew Black (2018), AmeriFlux BASE CA-Ca2 British Columbia - Clearcut Douglas-fir stand (harvested winter 1999/2000), Ver. 1-5, AmeriFlux AMP, (Dataset). https://doi.org/10.17190/AMF/1480301
CA-Ca3	2001-2021	T. Andrew Black (2023), AmeriFlux BASE CA-Ca3 British Columbia - Pole sapling Douglas-fir stand, Ver. 5-5, AmeriFlux AMP, (Dataset). https://doi.org/10.17190/AMF/1480302
CA-Cbo	1994-2020	Ralf Staebler (2022), AmeriFlux BASE CA-Cbo Ontario - Mixed Deciduous, Borden Forest Site, Ver. 6-5, AmeriFlux AMP, (Dataset). https://doi.org/10.17190/AMF/1498755
CA-Cha	2004-2005	Charles Bourque (2018), AmeriFlux BASE CA-Cha New Brunswick - Charlie Lake site 01 (immature balsam fir forest to be thinned in year 3), Ver. 1-5, AmeriFlux AMP, (Dataset). https://doi.org/10.17190/AMF/1436317
CA-DB2	2019-2020	Sara Knox (2021), AmeriFlux BASE CA-DB2 Delta Burns Bog 2, Ver. 1-5, AmeriFlux AMP, (Dataset). https://doi.org/10.17190/AMF/1811362
CA-DBB	2014-2020	Andreas Christen, Sara Knox (2021), AmeriFlux BASE CA-DBB Delta Burns Bog, Ver. 2-5, AmeriFlux AMP, (Dataset). https://doi.org/10.17190/AMF/1543378

CA-ER1	2015-2021	Claudia Wagner-Riddle (2021), AmeriFlux BASE CA-ER1 Elora Research Station, Ver. 3-5, AmeriFlux AMP, (Dataset). https://doi.org/10.17190/AMF/1579541
CA-LP1	2007-2021	Thomas Andrew Black (2022), AmeriFlux BASE CA-LP1 British Columbia - Mountain pine beetle-attacked lodgepole pine stand, Ver. 3-5, AmeriFlux AMP, (Dataset). https://doi.org/10.17190/AMF/1660337
CA-MA1	2009-2011	Brian Amiro (2020), AmeriFlux BASE CA-MA1 Manitoba Agricultural Site 1, Ver. 1-5, AmeriFlux AMP, (Dataset). https://doi.org/10.17190/AMF/1617701
CA-MA2	2009-2011	Brian Amiro (2020), AmeriFlux BASE CA-MA2 Manitoba Agricultural Site 2, Ver. 1-5, AmeriFlux AMP, (Dataset). https://doi.org/10.17190/AMF/1617702
CA-MA3	2009-2011	Brian Amiro (2020), AmeriFlux BASE CA-MA3 Manitoba Agricultural Site 3, Ver. 1-5, AmeriFlux AMP, (Dataset). https://doi.org/10.17190/AMF/1617703
CA-Man	1994-2008	Brian Amiro (2016), AmeriFlux BASE CA-Man Manitoba - Northern Old Black Spruce (former BOREAS Northern Study Area), Ver. 2-1, AmeriFlux AMP, (Dataset). https://doi.org/10.17190/AMF/1245997
CA-Na1	2003-2005	Charles P.-A. Bourque (2018), AmeriFlux BASE CA-Na1 New Brunswick - 1967 Balsam Fir - Nashwaak Lake Site 01 (Mature balsam fir forest), Ver. 1-5, AmeriFlux AMP, (Dataset). https://doi.org/10.17190/AMF/1436319
CA-Oas	1996-2010	T. Andrew Black (2016), AmeriFlux BASE CA-Oas Saskatchewan - Western Boreal, Mature Aspen, Ver. 1-1, AmeriFlux AMP, (Dataset). https://doi.org/10.17190/AMF/1375197
CA-Obs	1997-2010	T. Andrew Black (2016), AmeriFlux BASE CA-Obs Saskatchewan - Western Boreal, Mature Black Spruce, Ver. 1-1, AmeriFlux AMP, (Dataset). https://doi.org/10.17190/AMF/1375198
CA-SF1	2003-2006	Brian Amiro (2020), AmeriFlux BASE CA-SF1 Saskatchewan - Western Boreal, forest burned in 1977, Ver. 2-5, AmeriFlux AMP, (Dataset). https://doi.org/10.17190/AMF/1246006
CA-SF2	2001-2006	Brian Amiro (2019), AmeriFlux BASE CA-SF2 Saskatchewan - Western Boreal, forest burned in 1989, Ver. 3-5, AmeriFlux AMP, (Dataset). https://doi.org/10.17190/AMF/1246007
CA-SF3	2001-2006	Brian Amiro (2020), AmeriFlux BASE CA-SF3 Saskatchewan - Western Boreal, forest burned in 1998, Ver. 2-5, AmeriFlux AMP, (Dataset). https://doi.org/10.17190/AMF/1246008
CA-TP1	2002-2017	M. Altaf Arain (2018), AmeriFlux BASE CA-TP1 Ontario - Turkey Point 2002 Plantation White Pine, Ver. 3-5, AmeriFlux AMP, (Dataset). https://doi.org/10.17190/AMF/1246009

CA-TP2	2002-2008	M. Altaf Arain (2018), AmeriFlux BASE CA-TP2 Ontario - Turkey Point 1989 Plantation White Pine, Ver. 2-5, AmeriFlux AMP, (Dataset). https://doi.org/10.17190/AMF/1246010
CA-TP3	2002-2017	M. Altaf Arain (2018), AmeriFlux BASE CA-TP3 Ontario - Turkey Point 1974 Plantation White Pine, Ver. 3-5, AmeriFlux AMP, (Dataset). https://doi.org/10.17190/AMF/1246011
CA-TP4	2002-2017	M. Altaf Arain (2018), AmeriFlux BASE CA-TP4 Ontario - Turkey Point 1939 Plantation White Pine, Ver. 4-5, AmeriFlux AMP, (Dataset). https://doi.org/10.17190/AMF/1246012
CA-TPD	2012-2017	M. Altaf Arain (2018), AmeriFlux BASE CA-TPD Ontario - Turkey Point Mature Deciduous, Ver. 2-5, AmeriFlux AMP, (Dataset). https://doi.org/10.17190/AMF/1246152
CL-SDF	2014-2022	Jorge Perez-Quezada, Juan J. Armesto (2022), AmeriFlux BASE CL-SDF Senda Darwin Forest, Ver. 1-5, AmeriFlux AMP, (Dataset). https://doi.org/10.17190/AMF/1902273
CL-SDP	2014-2022	Jorge Perez-Quezada, Juan J. Armesto (2022), AmeriFlux BASE CL-SDP Senda Darwin Peatland, Ver. 1-5, AmeriFlux AMP, (Dataset). https://doi.org/10.17190/AMF/1902274
CR-SoC	2014-2018	Anthony T. Cahill, Georgianne W. Moore, Gretchen R. Miller, Jaeyoung Song (2022), AmeriFlux BASE CR-SoC Soltis Center, Ver. 1-5, AmeriFlux AMP, (Dataset). https://doi.org/10.17190/AMF/1880911
MX-Aog	2015-2018	Enrico A. Yepez (2020), AmeriFlux BASE MX-Aog Alamos Old-Growth tropical dry forest, Ver. 1-5, AmeriFlux AMP, (Dataset). https://doi.org/10.17190/AMF/1756414
MX-PMm	2017-2018	Ma. Susana Alvarado-Barrientos (2021), AmeriFlux BASE MX-PMm Puerto Morelos mangrove, Ver. 2-5, AmeriFlux AMP, (Dataset). https://doi.org/10.17190/AMF/1756415
MX-Tes	2004-2008	Enrico A. Yepez, Jaime Garatuza (2021), AmeriFlux BASE MX-Tes Tesopaco, secondary tropical dry forest, Ver. 2-5, AmeriFlux AMP, (Dataset). https://doi.org/10.17190/AMF/1767832
PE-QFR	2018-2019	Tyler Roman, Timothy Griffis, Randy Kolka, Craig Wayson, Erik Lilleskov, Dennis del Castillo Torres, Lizardo Fachin Malaverri, Jhon Ever Rengifo Marin (2021), AmeriFlux BASE PE-QFR Quistococha Forest Reserve, Ver. 2-5, AmeriFlux AMP, (Dataset). https://doi.org/10.17190/AMF/1671889
PR-xGU	2018-2022	NEON (National Ecological Observatory Network) (2022), AmeriFlux BASE PR-xGU NEON Guanica Forest (GUAN), Ver. 4-5, AmeriFlux AMP, (Dataset).

		https://doi.org/10.17190/AMF/1773393
PR-xLA	2018-2022	NEON (National Ecological Observatory Network) (2022), AmeriFlux BASE PR-xLA NEON Lajas Experimental Station (LAJA), Ver. 4-5, AmeriFlux AMP, (Dataset). https://doi.org/10.17190/AMF/1773394
US-A03	2014-2021	Dave Billesbach, Ryan Sullivan (2022), AmeriFlux BASE US-A03 ARM-AMF3-Oliktok, Ver. 5-5, AmeriFlux AMP, (Dataset). https://doi.org/10.17190/AMF/1498752
US-A10	2011-2020	Dave Billesbach, Ryan Sullivan (2021), AmeriFlux BASE US-A10 ARM-NSA-Barrow, Ver. 4-5, AmeriFlux AMP, (Dataset). https://doi.org/10.17190/AMF/1498753
US-A32	2015-2017	Dave Billesbach, Lara Kueppers, Margaret Torn, Sebastien Biraud (2018), AmeriFlux BASE US-A32 ARM-SGP Medford hay pasture, Ver. 1-5, AmeriFlux AMP, (Dataset). https://doi.org/10.17190/AMF/1436327
US-A74	2015-2017	Dave Billesbach, Lara Kueppers, Margaret Torn, Sebastien Biraud (2018), AmeriFlux BASE US-A74 ARM SGP milo field, Ver. 1-5, AmeriFlux AMP, (Dataset). https://doi.org/10.17190/AMF/1436328
US-Act	2017-2018	Sarah Waldo (2022), AmeriFlux BASE US-Act Acton Lake Flux Tower Site, Ver. 1-5, AmeriFlux AMP, (Dataset). https://doi.org/10.17190/AMF/1846660
US-Akn	2011-2022	Monique Leclerc (2023), AmeriFlux BASE US-Akn Savannah River Site, Ver. 6-5, AmeriFlux AMP, (Dataset). https://doi.org/10.17190/AMF/1246141
US-ALQ	2015-2022	Brent Olson (2023), AmeriFlux BASE US-ALQ Allequash Creek Site, Ver. 12-5, AmeriFlux AMP, (Dataset). https://doi.org/10.17190/AMF/1480323
US-AR1	2009-2012	Dave Billesbach, James Bradford, Margaret Torn (2019), AmeriFlux BASE US-AR1 ARM USDA UNL OSU Woodward Switchgrass 1, Ver. 3-5, AmeriFlux AMP, (Dataset). https://doi.org/10.17190/AMF/1246137
US-AR2	2009-2012	Dave Billesbach, James Bradford, Margaret Torn (2019), AmeriFlux BASE US-AR2 ARM USDA UNL OSU Woodward Switchgrass 2, Ver. 3-5, AmeriFlux AMP, (Dataset). https://doi.org/10.17190/AMF/1246138
US-ARM	2003-2023	Sebastien Biraud, Marc Fischer, Stephen Chan, Margaret Torn (2023), AmeriFlux BASE US-ARM ARM Southern Great Plains site- Lamont, Ver. 12-5, AmeriFlux AMP, (Dataset). https://doi.org/10.17190/AMF/1246027
US-ASH	2016-2017	Ray G. Anderson (2020), AmeriFlux BASE US-ASH USSL San Joaquin Valley Almond High Salinity, Ver. 1-5, AmeriFlux AMP, (Dataset). https://doi.org/10.17190/AMF/1634880
US-ASM	2016-2017	Ray G. Anderson (2020), AmeriFlux BASE US-ASM USSL San Joaquin Valley Almond Medium Salinity, Ver. 1-5, AmeriFlux AMP, (Dataset).

		https://doi.org/10.17190/AMF/1617709
US-Bar	2004-2017	Andrew Richardson, David Hollinger (2019), AmeriFlux BASE US-Bar Bartlett Experimental Forest, Ver. 5-5, AmeriFlux AMP, (Dataset). https://doi.org/10.17190/AMF/1246030
US-Bi1	2016-2022	Camilo Rey-Sanchez, Carlos Tianxin Wang, Daphne Szutu, Robert Shortt, Samuel D. Chamberlain, Joseph Verfaillie, Dennis Baldocchi (2022), AmeriFlux BASE US-Bi1 Bouldin Island Alfalfa, Ver. 8-5, AmeriFlux AMP, (Dataset). https://doi.org/10.17190/AMF/1480317
US-Bi2	2017-2022	Camilo Rey-Sanchez, Carlos Tianxin Wang, Daphne Szutu, Kyle Hemes, Joseph Verfaillie, Dennis Baldocchi (2022), AmeriFlux BASE US-Bi2 Bouldin Island corn, Ver. 13-5, AmeriFlux AMP, (Dataset). https://doi.org/10.17190/AMF/1419513
US-Blo	1997-2007	Allen Goldstein (2019), AmeriFlux BASE US-Blo Blodgett Forest, Ver. 4-5, AmeriFlux AMP, (Dataset). https://doi.org/10.17190/AMF/1246032
US-BMM	2016-2019	Paul Stoy, E. N. J. Brookshire (2022), AmeriFlux BASE US-BMM Bangtail Mountain Meadow, Ver. 3-5, AmeriFlux AMP, (Dataset). https://doi.org/10.17190/AMF/1660338
US-Bo1	1996-2008	Tilden Meyers (2016), AmeriFlux BASE US-Bo1 Bondville, Ver. 2-1, AmeriFlux AMP, (Dataset). https://doi.org/10.17190/AMF/1246036
US-Bo2	2004-2008	Carl Bernacchi (2016), AmeriFlux BASE US-Bo2 Bondville (companion site), Ver. 2-1, AmeriFlux AMP, (Dataset). https://doi.org/10.17190/AMF/1246037
US-BRG	2016-2020	Kimberly Novick (2020), AmeriFlux BASE US-BRG Bayles Road Grassland Tower, Ver. 1-5, AmeriFlux AMP, (Dataset). https://doi.org/10.17190/AMF/1756416
US-Bsg	2014-2015	Chris Still (2022), AmeriFlux BASE US-Bsg Burns Sagebrush, Ver. 1-5, AmeriFlux AMP, (Dataset). https://doi.org/10.17190/AMF/1846661
US-BZB	2011-2022	Eugenie Euskirchen (2022), AmeriFlux BASE US-BZB Bonanza Creek Thermokarst Bog, Ver. 4-5, AmeriFlux AMP, (Dataset). https://doi.org/10.17190/AMF/1773401
US-BZF	2011-2022	Eugenie Euskirchen (2022), AmeriFlux BASE US-BZF Bonanza Creek Rich Fen, Ver. 4-5, AmeriFlux AMP, (Dataset). https://doi.org/10.17190/AMF/1756433
US-BZo	2018-2022	Eugenie Euskirchen (2022), AmeriFlux BASE US-BZo Bonanza Creek Old Thermokarst Bog, Ver. 3-5, AmeriFlux AMP, (Dataset). https://doi.org/10.17190/AMF/1846662
US-BZS	2010-2021	Eugenie Euskirchen (2022), AmeriFlux BASE US-BZS Bonanza Creek Black Spruce, Ver. 3-5, AmeriFlux AMP, (Dataset). https://doi.org/10.17190/AMF/1756434

US-CdM	2019-2021	David Bowling, Steve Kannenberg, William Anderegg (2022), AmeriFlux BASE US-CdM Cedar Mesa, Ver. 1-5, AmeriFlux AMP, (Dataset). https://doi.org/10.17190/AMF/1865477
US-Ced	2005-2014	Ken Clark (2016), AmeriFlux BASE US-Ced Cedar Bridge, Ver. 7-1, AmeriFlux AMP, (Dataset). https://doi.org/10.17190/AMF/1246043
US-CF1	2017-2021	Claire L. Phillips, Dave Huggins (2022), AmeriFlux BASE US-CF1 CAF-LTAR Cook East, Ver. 3-5, AmeriFlux AMP, (Dataset). https://doi.org/10.17190/AMF/1543382
US-CF2	2017-2021	Dave Huggins (2021), AmeriFlux BASE US-CF2 CAF-LTAR Cook West, Ver. 2-5, AmeriFlux AMP, (Dataset). https://doi.org/10.17190/AMF/1543383
US-CF3	2017-2021	Dave Huggins (2022), AmeriFlux BASE US-CF3 CAF-LTAR Boyd North, Ver. 3-5, AmeriFlux AMP, (Dataset). https://doi.org/10.17190/AMF/1543385
US-CF4	2017-2021	Dave Huggins (2022), AmeriFlux BASE US-CF4 CAF-LTAR Boyd South, Ver. 3-5, AmeriFlux AMP, (Dataset). https://doi.org/10.17190/AMF/1543384
US-CMW	2000-2021	Russell Scott (2022), AmeriFlux BASE US-CMW Charleston Mesquite Woodland, Ver. 2-5, AmeriFlux AMP, (Dataset). https://doi.org/10.17190/AMF/1660339
US-Cop	2001-2007	David Bowling (2019), AmeriFlux BASE US-Cop Corral Pocket, Ver. 2-5, AmeriFlux AMP, (Dataset). https://doi.org/10.17190/AMF/1246129
US-CPk	2009-2013	Brent Ewers, Mario Bretfeld, Elise Pendall (2016), AmeriFlux BASE US-CPk Chimney Park, Ver. 2-1, AmeriFlux AMP, (Dataset). https://doi.org/10.17190/AMF/1246150
US-CRT	2011-2013	Jiquan Chen, Housen Chu (2021), AmeriFlux BASE US-CRT Curtice Walter-Berger cropland, Ver. 5-5, AmeriFlux AMP, (Dataset). https://doi.org/10.17190/AMF/1246156
US-CS1	2018-2019	Ankur Desai (2021), AmeriFlux BASE US-CS1 Central Sands Irrigated Agricultural Field, Ver. 2-5, AmeriFlux AMP, (Dataset). https://doi.org/10.17190/AMF/1617710
US-CS2	2018-2021	Ankur Desai (2022), AmeriFlux BASE US-CS2 Tri county school Pine Forest, Ver. 4-5, AmeriFlux AMP, (Dataset). https://doi.org/10.17190/AMF/1617711
US-CS3	2019-2020	Ankur Desai (2021), AmeriFlux BASE US-CS3 Central Sands Irrigated Agricultural Field, Ver. 3-5, AmeriFlux AMP, (Dataset). https://doi.org/10.17190/AMF/1617713
US-CS4	2020-2021	Ankur Desai (2022), AmeriFlux BASE US-CS4 Central Sands Irrigated Agricultural Field, Ver. 3-5, AmeriFlux AMP, (Dataset). https://doi.org/10.17190/AMF/1756417

US-CS5	2021-2021	Ankur Desai (2022), AmeriFlux BASE US-CS5 Central Sands Irrigated Agricultural Field, Ver. 1-5, AmeriFlux AMP, (Dataset). https://doi.org/10.17190/AMF/1846663
US-DFC	2018-2020	Alison Duff, Ankur Desai (2020), AmeriFlux BASE US-DFC US Dairy Forage Research Center, Prairie du Sac, Ver. 1-5, AmeriFlux AMP, (Dataset). https://doi.org/10.17190/AMF/1660340
US-DFK	2018-2021	Alison Duff, Ankur Desai, Valentin Picasso Risso (2021), AmeriFlux BASE US-DFK Dairy Forage Research Center - Kernza, Ver. 1-5, AmeriFlux AMP, (Dataset). https://doi.org/10.17190/AMF/1825937
US-Dia	2010-2012	Sonia Wharton (2016), AmeriFlux BASE US-Dia Diablo, Ver. 1-1, AmeriFlux AMP, (Dataset). https://doi.org/10.17190/AMF/1246146
US-Dix	2005-2008	Ken Clark (2016), AmeriFlux BASE US-Dix Fort Dix, Ver. 2-1, AmeriFlux AMP, (Dataset). https://doi.org/10.17190/AMF/1246045
US-Dk1	2001-2008	Chris Oishi, Kim Novick, Paul Stoy (2018), AmeriFlux BASE US-Dk1 Duke Forest-open field, Ver. 4-5, AmeriFlux AMP, (Dataset). https://doi.org/10.17190/AMF/1246046
US-Dk2	2001-2008	Chris Oishi, Kim Novick, Paul Stoy (2018), AmeriFlux BASE US-Dk2 Duke Forest-hardwoods, Ver. 4-5, AmeriFlux AMP, (Dataset). https://doi.org/10.17190/AMF/1246047
US-Dk3	2001-2008	Chris Oishi, Kim Novick, Paul Stoy (2018), AmeriFlux BASE US-Dk3 Duke Forest - loblolly pine, Ver. 4-5, AmeriFlux AMP, (Dataset). https://doi.org/10.17190/AMF/1246048
US-DPW	2013-2017	Charlless Ross Hinkle (2019), AmeriFlux BASE US-DPW Disney Wilderness Preserve Wetland, Ver. 1-5, AmeriFlux AMP, (Dataset). https://doi.org/10.17190/AMF/1562387
US-DS3	2021-2022	Michael R. Schuppenhauer, Sebastien C. Biraud, Steve Deverel (2022), AmeriFlux BASE US-DS3 Staten Rice 1, Ver. 1-5, AmeriFlux AMP, (Dataset). https://doi.org/10.17190/AMF/1890490
US-EDN	2018-2019	Patty Oikawa (2020), AmeriFlux BASE US-EDN Eden Landing Ecological Reserve, Ver. 2-5, AmeriFlux AMP, (Dataset). https://doi.org/10.17190/AMF/1543381
US-Elm	2008-2014	Gregory Starr, Steve Oberbauer (2016), AmeriFlux BASE US-Elm Everglades (long hydroperiod marsh), Ver. 4-1, AmeriFlux AMP, (Dataset). https://doi.org/10.17190/AMF/1246118
US-EML	2008-2020	Ted Schuur (2021), AmeriFlux BASE US-EML Eight Mile Lake Permafrost thaw gradient, Healy Alaska., Ver. 4-5, AmeriFlux AMP, (Dataset).

		https://doi.org/10.17190/AMF/1418678
US-Esm	2008-2015	Gregory Starr, Steve Oberbauer (2016), AmeriFlux BASE US-Esm Everglades (short hydroperiod marsh), Ver. 5-1, AmeriFlux AMP, (Dataset). https://doi.org/10.17190/AMF/1246119
US-Fcr	2011-2014	Masahito Ueyama, Hiroki Iwata, Yoshinobu Harazono (2019), AmeriFlux BASE US-Fcr Cascaden Ridge Fire Scar, Ver. 2-5, AmeriFlux AMP, (Dataset). https://doi.org/10.17190/AMF/1562388
US-Fmf	2005-2010	Sabina Dore, Thomas Kolb (2019), AmeriFlux BASE US-Fmf Flagstaff - Managed Forest, Ver. 6-5, AmeriFlux AMP, (Dataset). https://doi.org/10.17190/AMF/1246050
US-Fuf	2005-2010	Sabina Dore, Thomas Kolb (2019), AmeriFlux BASE US-Fuf Flagstaff - Unmanaged Forest, Ver. 6-5, AmeriFlux AMP, (Dataset). https://doi.org/10.17190/AMF/1246051
US-Fwf	2005-2010	Sabina Dore, Thomas Kolb (2019), AmeriFlux BASE US-Fwf Flagstaff - Wildfire, Ver. 8-5, AmeriFlux AMP, (Dataset). https://doi.org/10.17190/AMF/1246052
US-GBT	1999-2006	Bill Massman (2016), AmeriFlux BASE US-GBT GLEES Brooklyn Tower, Ver. 1-1, AmeriFlux AMP, (Dataset). https://doi.org/10.17190/AMF/1375200
US-GLE	1999-2020	John Frank, Bill Massman (2021), AmeriFlux BASE US-GLE GLEES, Ver. 8-5, AmeriFlux AMP, (Dataset). https://doi.org/10.17190/AMF/1246056
US-Ha1	1991-2022	J. William Munger (2022), AmeriFlux BASE US-Ha1 Harvard Forest EMS Tower (HFR1), Ver. 19-5, AmeriFlux AMP, (Dataset). https://doi.org/10.17190/AMF/1246059
US-Ha2	2004-2022	Julian Hadley, J. William Munger (2023), AmeriFlux BASE US-Ha2 Harvard Forest Hemlock Site, Ver. 9-5, AmeriFlux AMP, (Dataset). https://doi.org/10.17190/AMF/1246060
US-HB1	2019-2021	Jeremy D. Forsythe, Michael A. Kline, Thomas L. O’Halloran (2022), AmeriFlux BASE US-HB1 North Inlet Crab Haul Creek, Ver. 2-5, AmeriFlux AMP, (Dataset). https://doi.org/10.17190/AMF/1660341
US-HB2	2019-2019	Jeremy D. Forsythe, Michael A. Kline, Thomas L. O’Halloran (2020), AmeriFlux BASE US-HB2 Hobcaw Barony Mature Longleaf Pine, Ver. 1-5, AmeriFlux AMP, (Dataset). https://doi.org/10.17190/AMF/1660342
US-HB3	2019-2019	Jeremy D. Forsythe, Michael A. Kline, Thomas L. O’Halloran (2020), AmeriFlux BASE US-HB3 Hobcaw Barony Longleaf Pine Restoration, Ver. 1-5, AmeriFlux AMP, (Dataset). https://doi.org/10.17190/AMF/1660343
US-HBK	2016-2020	Eric Kelsey, Mark Green (2020), AmeriFlux BASE US-HBK Hubbard Brook

		Experimental Forest, Ver. 1-5, AmeriFlux AMP, (Dataset). https://doi.org/10.17190/AMF/1634881
US-Hn2	2015-2018	Heping Liu, Maoyi Huang, Xingyuan Chen (2019), AmeriFlux BASE US-Hn2 Hanford 100H grassland, Ver. 1-5, AmeriFlux AMP, (Dataset). https://doi.org/10.17190/AMF/1562389
US-Hn3	2017-2018	Heping Liu, Maoyi Huang, Xingyuan Chen (2019), AmeriFlux BASE US-Hn3 Hanford 100H sagebrush, Ver. 1-5, AmeriFlux AMP, (Dataset). https://doi.org/10.17190/AMF/1543379
US-Ho1	1995-2020	David Hollinger (2021), AmeriFlux BASE US-Ho1 Howland Forest (main tower), Ver. 7-5, AmeriFlux AMP, (Dataset). https://doi.org/10.17190/AMF/1246061
US-Ho2	1999-2020	David Hollinger (2021), AmeriFlux BASE US-Ho2 Howland Forest (west tower), Ver. 4-5, AmeriFlux AMP, (Dataset). https://doi.org/10.17190/AMF/1246062
US-Ho3	2003-2009	David Hollinger (2016), AmeriFlux BASE US-Ho3 Howland Forest (harvest site), Ver. 2-1, AmeriFlux AMP, (Dataset). https://doi.org/10.17190/AMF/1246063
US-HRA	2015-2017	Benjamin R. K. Runkle (2021), AmeriFlux BASE US-HRA Humnoke Farm Rice Field â€“ Field A, Ver. 3-5, AmeriFlux AMP, (Dataset). https://doi.org/10.17190/AMF/1543376
US-HRC	2015-2017	Michele L. Reba (2021), AmeriFlux BASE US-HRC Humnoke Farm Rice Field â€“ Field C, Ver. 3-5, AmeriFlux AMP, (Dataset). https://doi.org/10.17190/AMF/1543375
US-Hsm	2021-2022	Ariane Arias-Ortiz, Daphne Szutu, Joseph Verfaillie, Dennis Baldocchi (2022), AmeriFlux BASE US-Hsm Hill Slough Marsh, Ver. 1-5, AmeriFlux AMP, (Dataset). https://doi.org/10.17190/AMF/1890483
US-HWB	2015-2018	Sarah Goslee (2021), AmeriFlux BASE US-HWB USDA ARS Pasture Sytems and Watershed Management Research Unit- Hawbecker Site, Ver. 1-5, AmeriFlux AMP, (Dataset). https://doi.org/10.17190/AMF/1811363
US-ICh	2007-2021	Eugenie Euskirchen, Gaius Shaver, Syndonia Bret-Harte (2022), AmeriFlux BASE US-ICh Imnavait Creek Watershed Heath Tundra, Ver. 4-5, AmeriFlux AMP, (Dataset). https://doi.org/10.17190/AMF/1246133
US-ICs	2007-2022	Eugenie Euskirchen, Gaius Shaver, Syndonia Bret-Harte (2022), AmeriFlux BASE US-ICs Imnavait Creek Watershed Wet Sedge Tundra, Ver. 7-5, AmeriFlux AMP, (Dataset). https://doi.org/10.17190/AMF/1246130
US-ICt	2007-2022	Eugenie Euskirchen, Gaius Shaver, Syndonia Bret-Harte (2022), AmeriFlux BASE US-ICt Imnavait Creek Watershed Tussock Tundra, Ver. 5-5, AmeriFlux AMP, (Dataset). https://doi.org/10.17190/AMF/1246131

US-Jo1	2010-2020	Craig Tweedie (2023), AmeriFlux BASE US-Jo1 Jornada Experimental Range Bajada Site, Ver. 3-5, AmeriFlux AMP, (Dataset). https://doi.org/10.17190/AMF/1767833
US-Jo2	2010-2020	Enrique R. Vivoni, Eli R. Perez-Ruiz (2022), AmeriFlux BASE US-Jo2 Jornada Experimental Range Mixed Shrubland, Ver. 2-5, AmeriFlux AMP, (Dataset). https://doi.org/10.17190/AMF/1617696
US-JRn	2017-2019	Derek Johnson, Gil Bohrer, Jaclyn Hatala Matthes (2020), AmeriFlux BASE US-JRn WV Jacks Run, Ver. 1-5, AmeriFlux AMP, (Dataset). https://doi.org/10.17190/AMF/1617714
US-KFS	2007-2019	Nathaniel Brunsell (2020), AmeriFlux BASE US-KFS Kansas Field Station, Ver. 7-5, AmeriFlux AMP, (Dataset). https://doi.org/10.17190/AMF/1246132
US-KL1	2009-2021	G. Philip Robertson, Jiquan Chen (2022), AmeriFlux BASE US-KL1 KBS Lux Arbor Reserve Corn, Ver. 3-5, AmeriFlux AMP, (Dataset). https://doi.org/10.17190/AMF/1660344
US-KL2	2009-2021	G. Philip Robertson, Jiquan Chen (2022), AmeriFlux BASE US-KL2 KBS Lux Arbor Reserve Switchgrass, Ver. 4-5, AmeriFlux AMP, (Dataset). https://doi.org/10.17190/AMF/1644212
US-KL3	2009-2021	G. Philip Robertson, Jiquan Chen (2022), AmeriFlux BASE US-KL3 KBS Lux Arbor Reserve Prairie, Ver. 4-5, AmeriFlux AMP, (Dataset). https://doi.org/10.17190/AMF/1647438
US-KLS	2012-2019	Nathaniel Brunsell (2021), AmeriFlux BASE US-KLS Kansas Land Institute, Ver. 2-5, AmeriFlux AMP, (Dataset). https://doi.org/10.17190/AMF/1498745
US-KM1	2009-2021	G. Philip Robertson, Jiquan Chen (2022), AmeriFlux BASE US-KM1 KBS Marshall Farms Corn, Ver. 4-5, AmeriFlux AMP, (Dataset). https://doi.org/10.17190/AMF/1647439
US-KM2	2009-2021	G. Philip Robertson, Jiquan Chen (2022), AmeriFlux BASE US-KM2 KBS Marshall Farms Prairie, Ver. 4-5, AmeriFlux AMP, (Dataset). https://doi.org/10.17190/AMF/1647440
US-KM3	2009-2021	G. Philip Robertson, Jiquan Chen (2022), AmeriFlux BASE US-KM3 KBS Marshall Farms Switchgrass, Ver. 3-5, AmeriFlux AMP, (Dataset). https://doi.org/10.17190/AMF/1660345
US-KM4	2009-2021	G. Philip Robertson, Jiquan Chen (2022), AmeriFlux BASE US-KM4 KBS Marshall Farms Smooth Brome Grass (Ref), Ver. 5-5, AmeriFlux AMP, (Dataset). https://doi.org/10.17190/AMF/1634882
US-Kon	2004-2019	Nathaniel Brunsell (2020), AmeriFlux BASE US-Kon Konza Prairie LTER (KNZ), Ver.

		5-5, AmeriFlux AMP, (Dataset). https://doi.org/10.17190/AMF/1246068
US-KPL	2021-2021	Patrick Sullivan (2022), AmeriFlux BASE US-KPL Lily Lake Fen, Ver. 1-5, AmeriFlux AMP, (Dataset). https://doi.org/10.17190/AMF/1865478
US-KS1	2002-2003	Bert Drake, Ross Hinkle (2019), AmeriFlux BASE US-KS1 Kennedy Space Center (slash pine), Ver. 3-5, AmeriFlux AMP, (Dataset). https://doi.org/10.17190/AMF/1246069
US-KS2	1999-2006	Bert Drake, Ross Hinkle (2019), AmeriFlux BASE US-KS2 Kennedy Space Center (scrub oak), Ver. 3-5, AmeriFlux AMP, (Dataset). https://doi.org/10.17190/AMF/1246070
US-KS3	2018-2019	Ross Hinkle (2019), AmeriFlux BASE US-KS3 Kennedy Space Center (salt marsh), Ver. 1-5, AmeriFlux AMP, (Dataset). https://doi.org/10.17190/AMF/1562390
US-KUT	2005-2009	Joe McFadden (2016), AmeriFlux BASE US-KUT KUOM Turfgrass Field, Ver. 1-1, AmeriFlux AMP, (Dataset). https://doi.org/10.17190/AMF/1246145
US-Lin	2009-2010	Silvano Fares (2019), AmeriFlux BASE US-Lin Lindcove Orange Orchard, Ver. 2-5, AmeriFlux AMP, (Dataset). https://doi.org/10.17190/AMF/1246830
US-LL1	2009-2020	Gregory Starr (2021), AmeriFlux BASE US-LL1 Longleaf Pine - Baker (Mesic site), Ver. 2-5, AmeriFlux AMP, (Dataset). https://doi.org/10.17190/AMF/1773395
US-LL2	2009-2017	Gregory Starr (2021), AmeriFlux BASE US-LL2 Longleaf Pine - Dubignion (Intermediate site), Ver. 1-5, AmeriFlux AMP, (Dataset). https://doi.org/10.17190/AMF/1773396
US-LL3	2009-2017	Gregory Starr (2021), AmeriFlux BASE US-LL3 Longleaf Pine - Red Dirt (Xeric site), Ver. 1-5, AmeriFlux AMP, (Dataset). https://doi.org/10.17190/AMF/1773397
US-Los	2000-2022	Ankur Desai (2023), AmeriFlux BASE US-Los Lost Creek, Ver. 24-5, AmeriFlux AMP, (Dataset). https://doi.org/10.17190/AMF/1246071
US-LS1	2002-2007	Russell Scott (2020), AmeriFlux BASE US-LS1 San Pedro River Lewis Springs Sacaton Grassland, Ver. 1-5, AmeriFlux AMP, (Dataset). https://doi.org/10.17190/AMF/1660346
US-LS2	2002-2007	Russell Scott (2020), AmeriFlux BASE US-LS2 San Pedro River Lewis Springs Savanna, Ver. 1-5, AmeriFlux AMP, (Dataset). https://doi.org/10.17190/AMF/1660347
US-Me2	2002-2022	Bev Law (2022), AmeriFlux BASE US-Me2 Metolius mature ponderosa pine, Ver. 18-5, AmeriFlux AMP, (Dataset). https://doi.org/10.17190/AMF/1246076
US-Me6	2010-2021	Bev Law (2021), AmeriFlux BASE US-Me6 Metolius Young Pine Burn, Ver. 15-5, AmeriFlux AMP, (Dataset). https://doi.org/10.17190/AMF/1246128

US-Men	2012-2018	Ankur Desai (2018), AmeriFlux BASE US-Men Lake Mendota, Center for Limnology Site, Ver. 3-5, AmeriFlux AMP, (Dataset). https://doi.org/10.17190/AMF/1433375
US-MH1	2015-2015	Paul Stoy, Kent McVay (2020), AmeriFlux BASE US-MH1 Huntley, Montana irrigated barley site 1, Ver. 1-5, AmeriFlux AMP, (Dataset). https://doi.org/10.17190/AMF/1660349
US-MH2	2016-2016	Paul Stoy, Kent McVay (2021), AmeriFlux BASE US-MH2 Huntley, Montana irrigated barley site 2, Ver. 1-5, AmeriFlux AMP, (Dataset). https://doi.org/10.17190/AMF/1825938
US-Mi1	2015-2019	Sarah Goslee (2022), AmeriFlux BASE US-Mi1 LTAR UCB (Upper Chesapeake Bay) Miscanthus 1, Ver. 1-5, AmeriFlux AMP, (Dataset). https://doi.org/10.17190/AMF/1865479
US-Mi2	2015-2017	Sarah Goslee (2022), AmeriFlux BASE US-Mi2 LTAR UCB (Upper Chesapeake Bay) Miscanthus 2, Ver. 1-5, AmeriFlux AMP, (Dataset). https://doi.org/10.17190/AMF/1865480
US-Mi3	2015-2019	Sarah Goslee (2022), AmeriFlux BASE US-Mi3 LTAR UCB (Upper Chesapeake Bay) Miscanthus 3, Ver. 1-5, AmeriFlux AMP, (Dataset). https://doi.org/10.17190/AMF/1865481
US-Mj2	2014-2014	Paul C. Stoy, Elizabeth Vick (2020), AmeriFlux BASE US-Mj2 Montana Judith Basin summer fallow field, Ver. 1-5, AmeriFlux AMP, (Dataset). https://doi.org/10.17190/AMF/1617716
US-MMS	1999-2022	Kim Novick, Rich Phillips (2023), AmeriFlux BASE US-MMS Morgan Monroe State Forest, Ver. 22-5, AmeriFlux AMP, (Dataset). https://doi.org/10.17190/AMF/1246080
US-MOz	2004-2021	Jeffrey Wood, Lianhong Gu (2022), AmeriFlux BASE US-MOz Missouri Ozark Site, Ver. 11-5, AmeriFlux AMP, (Dataset). https://doi.org/10.17190/AMF/1246081
US-Mpj	2008-2022	Marcy Litvak (2022), AmeriFlux BASE US-Mpj Mountainair Pinyon-Juniper Woodland, Ver. 20-5, AmeriFlux AMP, (Dataset). https://doi.org/10.17190/AMF/1246123
US-MtB	2009-2021	Greg Barron-Gafford (2022), AmeriFlux BASE US-MtB Mt Bigelow, Ver. 4-5, AmeriFlux AMP, (Dataset). https://doi.org/10.17190/AMF/1579717
US-MVW	2017-2017	Paul Stoy (2021), AmeriFlux BASE US-MVW Montana Vaughn Wheat, Ver. 1-5, AmeriFlux AMP, (Dataset). https://doi.org/10.17190/AMF/1829508
US-Myb	2010-2021	Jaclyn Hatala Matthes, Cove Sturtevant, Patty Oikawa, Samuel D Chamberlain, Daphne Szutu, Ariane Arias-Ortiz, Joseph Verfaillie, Dennis Baldocchi (2022),

		AmeriFlux BASE US-Myb Mayberry Wetland, Ver. 13-5, AmeriFlux AMP, (Dataset). https://doi.org/10.17190/AMF/1246139
US-NC1	2005-2012	Asko Noormets, Ge Sun, Michael Gavazzi, Steve McNulty, Jean-Christophe Domec, John King (2018), AmeriFlux BASE US-NC1 NC_Clearcut, Ver. 3-5, AmeriFlux AMP, (Dataset). https://doi.org/10.17190/AMF/1246082
US-NC2	2005-2021	Asko Noormets, Ge Sun, Michael Gavazzi, Jean-Christophe Domec, Steve McNulty, Guofang Miao, Maricar Aguilos, Bhaskar Mitra, Kevan Minick, John King, Linqing Yang, Prajaya Prajapati (2022), AmeriFlux BASE US-NC2 NC_Loblolly Plantation, Ver. 10-5, AmeriFlux AMP, (Dataset). https://doi.org/10.17190/AMF/1246083
US-NC3	2013-2021	Asko Noormets, Michael Gavazzi, Maricar Aguilos, John King, Bhaskar Mitra, Jean-Christophe Domec (2022), AmeriFlux BASE US-NC3 NC_Clearcut#3, Ver. 4-5, AmeriFlux AMP, (Dataset). https://doi.org/10.17190/AMF/1419506
US-NC4	2009-2021	Miao, G., Noormets, A., Domec, J., Fuentes, M., Trettin, C. C., Sun, G., McNulty, S. G., King, J. S. (2017), Hydrology And Microtopography Control Carbon Dynamics In Wetlands: Implications In Partitioning Ecosystem Respiration In A Coastal Plain Forested Wetland Agricultural And Forest Meteorology, 247, 343-355 DOI: 10.1016/j.agrformet.2017.08.022
US-Ne1	2001-2020	Andy Suyker (2023), AmeriFlux BASE US-Ne1 Mead - irrigated continuous maize site, Ver. 14-5, AmeriFlux AMP, (Dataset). https://doi.org/10.17190/AMF/1246084
US-Ne2	2001-2020	Andy Suyker (2023), AmeriFlux BASE US-Ne2 Mead - irrigated maize-soybean rotation site, Ver. 14-5, AmeriFlux AMP, (Dataset). https://doi.org/10.17190/AMF/1246085
US-Ne3	2001-2020	Andy Suyker (2023), AmeriFlux BASE US-Ne3 Mead - rainfed maize-soybean rotation site, Ver. 14-5, AmeriFlux AMP, (Dataset). https://doi.org/10.17190/AMF/1246086
US-NGB	2012-2021	Margaret Torn, Sigrid Dengel (2022), AmeriFlux BASE US-NGB NGEE Arctic Barrow, Ver. 4-5, AmeriFlux AMP, (Dataset). https://doi.org/10.17190/AMF/1436326
US-NGC	2017-2022	Margaret Torn, Sigrid Dengel (2022), AmeriFlux BASE US-NGC NGEE Arctic Council, Ver. 2-5, AmeriFlux AMP, (Dataset). https://doi.org/10.17190/AMF/1634883
US-NMj	2001-2003	Jiquan Chen (2019), AmeriFlux BASE US-NMj Northern Michigan Jack Pine Stand, Ver. 3-5, AmeriFlux AMP, (Dataset). https://doi.org/10.17190/AMF/1246087
US-NR1	1998-2021	Peter D. Blanken, Russel K. Monson, Sean P. Burns, David R. Bowling, Andrew A.

		Turnipseed (2022), AmeriFlux BASE US-NR1 Niwot Ridge Forest (LTER NWT1), Ver. 19-5, AmeriFlux AMP, (Dataset). https://doi.org/10.17190/AMF/1246088
US-NR3	2007-2021	John Knowles (2022), AmeriFlux BASE US-NR3 Niwot Ridge Alpine (T-Van West), Ver. 3-5, AmeriFlux AMP, (Dataset). https://doi.org/10.17190/AMF/1804491
US-NR4	2007-2021	John Knowles (2022), AmeriFlux BASE US-NR4 Niwot Ridge Alpine (T-Van East), Ver. 3-5, AmeriFlux AMP, (Dataset). https://doi.org/10.17190/AMF/1804492
US-Oho	2004-2013	Jiquan Chen, Housen Chu, Asko Noormets (2021), AmeriFlux BASE US-Oho Oak Openings, Ver. 7-5, AmeriFlux AMP, (Dataset). https://doi.org/10.17190/AMF/1246089
US-ONA	2016-2021	Maria Silveira (2022), AmeriFlux BASE US-ONA Florida pine flatwoods, Ver. 3-5, AmeriFlux AMP, (Dataset). https://doi.org/10.17190/AMF/1660350
US-ORv	2011-2016	Gil Bohrer (2020), AmeriFlux BASE US-ORv Olentangy River Wetland Research Park, Ver. 3-5, AmeriFlux AMP, (Dataset). https://doi.org/10.17190/AMF/1246135
US-OWC	2015-2022	Gil Bohrer, Janice Kerns (2022), AmeriFlux BASE US-OWC Old Woman Creek, Ver. 3-5, AmeriFlux AMP, (Dataset). https://doi.org/10.17190/AMF/1418679
US-PAS	2021-2021	Maria Lucia Silveira, Rosvel Bracho (2022), AmeriFlux BASE US-PAS Florida, Paspalum notatum pasture, Ver. 1-5, AmeriFlux AMP, (Dataset). https://doi.org/10.17190/AMF/1870590
US-PFa	1995-2022	Ankur Desai (2023), AmeriFlux BASE US-PFa Park Falls/WLEF, Ver. 23-5, AmeriFlux AMP, (Dataset). https://doi.org/10.17190/AMF/1246090
US-PFb	2019-2019	Ankur Desai, Brian Butterworth, Steven Oncley (2020), AmeriFlux BASE US-PFb NW1 Pine-1 CHEESEHEAD 2019, Ver. 1-5, AmeriFlux AMP, (Dataset). https://doi.org/10.17190/AMF/1717850
US-PFc	2019-2019	Ankur Desai, Brian Butterworth, Steven Oncley (2020), AmeriFlux BASE US-PFc NW2 Aspen-1 CHEESEHEAD 2019, Ver. 1-5, AmeriFlux AMP, (Dataset). https://doi.org/10.17190/AMF/1717851
US-PFd	2019-2019	Ankur Desai, Brian Butterworth, Steven Oncley (2020), AmeriFlux BASE US-PFd NW3 Tussock-1 CHEESEHEAD 2019, Ver. 1-5, AmeriFlux AMP, (Dataset). https://doi.org/10.17190/AMF/1717852
US-PFe	2019-2019	Ankur Desai, Brian Butterworth, Steven Oncley (2020), AmeriFlux BASE US-PFe NW4 Lake-1 CHEESEHEAD 2019, Ver. 1-5, AmeriFlux AMP, (Dataset). https://doi.org/10.17190/AMF/1717853
US-PFg	2019-2019	Ankur Desai, Brian Butterworth, Steven Oncley (2020), AmeriFlux BASE US-PFg NE1 Pine-2 CHEESEHEAD 2019, Ver. 1-5, AmeriFlux AMP, (Dataset).

		https://doi.org/10.17190/AMF/1717854
US-PFh	2019-2019	Ankur Desai, Brian Butterworth, Steven Oncley (2020), AmeriFlux BASE US-PFh NE2 Pine-3 CHEESEHEAD 2019, Ver. 1-5, AmeriFlux AMP, (Dataset). https://doi.org/10.17190/AMF/1717855
US-PFj	2019-2019	Ankur Desai, Brian Butterworth, Steven Oncley (2020), AmeriFlux BASE US-PFj NE4 Maple-1 CHEESEHEAD 2019, Ver. 1-5, AmeriFlux AMP, (Dataset). https://doi.org/10.17190/AMF/1717857
US-PFk	2019-2019	Ankur Desai, Brian Butterworth, Steven Oncley (2020), AmeriFlux BASE US-PFk SW1 Aspen-2 CHEESEHEAD 2019, Ver. 1-5, AmeriFlux AMP, (Dataset). https://doi.org/10.17190/AMF/1717858
US-PFL	2019-2019	Ankur Desai, Brian Butterworth, Steven Oncley (2020), AmeriFlux BASE US-PFL SW2 Aspen-3 CHEESEHEAD 2019, Ver. 1-5, AmeriFlux AMP, (Dataset). https://doi.org/10.17190/AMF/1717859
US-PFm	2019-2019	Ankur Desai, Brian Butterworth, Steven Oncley (2020), AmeriFlux BASE US-PFm SW3 Hardwood-2 CHEESEHEAD 2019, Ver. 1-5, AmeriFlux AMP, (Dataset). https://doi.org/10.17190/AMF/1717860
US-PFn	2019-2019	Ankur Desai, Brian Butterworth, Steven Oncley (2020), AmeriFlux BASE US-PFn SW4 Hardwood-3 CHEESEHEAD 2019, Ver. 1-5, AmeriFlux AMP, (Dataset). https://doi.org/10.17190/AMF/1717861
US-PFo	2019-2019	Ankur Desai, Brian Butterworth, Jonathan Thom, Paul Stoy (2022), AmeriFlux BASE US-PFo SE1 Lake-2 CHEESEHEAD 2019, Ver. 1-5, AmeriFlux AMP, (Dataset). https://doi.org/10.17190/AMF/1880912
US-PFp	2019-2019	Ankur Desai, Brian Butterworth, Steven Oncley (2020), AmeriFlux BASE US-PFp SE2 Hardwood-4 CHEESEHEAD 2019, Ver. 1-5, AmeriFlux AMP, (Dataset). https://doi.org/10.17190/AMF/1717862
US-PFq	2019-2019	Ankur Desai, Brian Butterworth, Steven Oncley (2020), AmeriFlux BASE US-PFq SE3 Aspen-4 CHEESEHEAD 2019, Ver. 1-5, AmeriFlux AMP, (Dataset). https://doi.org/10.17190/AMF/1717863
US-PFr	2019-2019	Ankur Desai, Brian Butterworth, Steven Oncley (2020), AmeriFlux BASE US-PFr SE4 Tussock-2 CHEESEHEAD 2019, Ver. 1-5, AmeriFlux AMP, (Dataset). https://doi.org/10.17190/AMF/1717864
US-PFs	2019-2019	Ankur Desai, Brian Butterworth, Steven Oncley (2020), AmeriFlux BASE US-PFs SE5 Aspen-5 CHEESEHEAD 2019, Ver. 1-5, AmeriFlux AMP, (Dataset). https://doi.org/10.17190/AMF/1717865
US-PFt	2019-2019	Ankur Desai, Brian Butterworth, Steven Oncley (2020), AmeriFlux BASE US-PFt

		SE6 Pine-4 CHEESEHEAD 2019, Ver. 1-5, AmeriFlux AMP, (Dataset). https://doi.org/10.17190/AMF/1717866
US-PHM	2013-2020	Anne Giblin (2021), AmeriFlux BASE US-PHM Plum Island High Marsh, Ver. 3-5, AmeriFlux AMP, (Dataset). https://doi.org/10.17190/AMF/1543377
US-Pnp	2016-2022	Ankur Desai (2023), AmeriFlux BASE US-Pnp Lake Mendota, Picnic Point Site, Ver. 8-5, AmeriFlux AMP, (Dataset). https://doi.org/10.17190/AMF/1433376
US-Prr	2010-2016	Go Iwahana, Hideki Kobayashi, Hiroki Ikawa, Rikie Suzuki (2019), AmeriFlux BASE US-Prr Poker Flat Research Range Black Spruce Forest, Ver. 3-5, AmeriFlux AMP, (Dataset). https://doi.org/10.17190/AMF/1246153
US-PSH	2016-2017	Ray G. Anderson (2020), AmeriFlux BASE US-PSH USSL San Joaquin Valley Pistachio High, Ver. 1-5, AmeriFlux AMP, (Dataset). https://doi.org/10.17190/AMF/1617719
US-PSL	2016-2017	Ray G. Anderson (2020), AmeriFlux BASE US-PSL USSL San Joaquin Valley Pistachio Low, Ver. 1-5, AmeriFlux AMP, (Dataset). https://doi.org/10.17190/AMF/1617720
US-RGA	2021-2022	Michael R. Schuppenhauer, Sebastien C. Biraud (2023), AmeriFlux BASE US-RGA Arkansas Corn Farm, Ver. 2-5, AmeriFlux AMP, (Dataset). https://doi.org/10.17190/AMF/1880913
US-RGB	2021-2023	Michael Schuppenhauer, Sebastien C. Biraud (2023), AmeriFlux BASE US-RGB Butte County Rice Farm, Ver. 3-5, AmeriFlux AMP, (Dataset). https://doi.org/10.17190/AMF/1870591
US-RGo	2021-2023	Michael R. Schuppenhauer, Sebastien C. Biraud (2023), AmeriFlux BASE US-RGo Glenn County Organic Rice Farm, Ver. 2-5, AmeriFlux AMP, (Dataset). https://doi.org/10.17190/AMF/1880914
US-RGW	2022-2023	Michael R. Schuppenhauer, Sebastien C. Biraud (2023), AmeriFlux BASE US-RGW Desha County Rice Farm, Ver. 2-5, AmeriFlux AMP, (Dataset). https://doi.org/10.17190/AMF/1880915
US-Rls	2014-2020	Gerald Flerchinger (2021), AmeriFlux BASE US-Rls RCEW Low Sagebrush, Ver. 4-5, AmeriFlux AMP, (Dataset). https://doi.org/10.17190/AMF/1418682
US-Rms	2014-2020	Gerald Flerchinger (2021), AmeriFlux BASE US-Rms RCEW Mountain Big Sagebrush, Ver. 4-5, AmeriFlux AMP, (Dataset). https://doi.org/10.17190/AMF/1375202
US-Ro1	2004-2016	John Baker, Tim Griffis, Timothy Griffis (2018), AmeriFlux BASE US-Ro1 Rosemount- G21, Ver. 5-5, AmeriFlux AMP, (Dataset). https://doi.org/10.17190/AMF/1246092

US-Ro2	2008-2016	John Baker, Tim Griffis (2018), AmeriFlux BASE US-Ro2 Rosemount- C7, Ver. 1-5, AmeriFlux AMP, (Dataset). https://doi.org/10.17190/AMF/1418683
US-Ro3	2004-2007	John Baker, Tim Griffis (2019), AmeriFlux BASE US-Ro3 Rosemount- G19, Ver. 4-5, AmeriFlux AMP, (Dataset). https://doi.org/10.17190/AMF/1246093
US-Ro4	2014-2022	John Baker, Tim Griffis (2023), AmeriFlux BASE US-Ro4 Rosemount Prairie, Ver. 19-5, AmeriFlux AMP, (Dataset). https://doi.org/10.17190/AMF/1419507
US-Ro5	2017-2022	John Baker, Tim Griffis (2023), AmeriFlux BASE US-Ro5 Rosemount I18_South, Ver. 19-5, AmeriFlux AMP, (Dataset). https://doi.org/10.17190/AMF/1419508
US-Ro6	2017-2022	John Baker, Tim Griffis (2023), AmeriFlux BASE US-Ro6 Rosemount I18_North, Ver. 19-5, AmeriFlux AMP, (Dataset). https://doi.org/10.17190/AMF/1419509
US-Rpf	2008-2022	Masahito Ueyama, Hiroki Iwata, Yoshinobu Harazono (2023), AmeriFlux BASE US-Rpf Poker Flat Research Range: Succession from fire scar to deciduous forest, Ver. 8-5, AmeriFlux AMP, (Dataset). https://doi.org/10.17190/AMF/1579540
US-Rwe	2003-2007	Gerald Flerchinger, Michele L. Reba (2020), AmeriFlux BASE US-Rwe RCEW Reynolds Mountain East, Ver. 1-5, AmeriFlux AMP, (Dataset). https://doi.org/10.17190/AMF/1617721
US-Rwf	2014-2020	Gerald Flerchinger (2021), AmeriFlux BASE US-Rwf RCEW Upper Sheep Prescibed Fire, Ver. 2-5, AmeriFlux AMP, (Dataset). https://doi.org/10.17190/AMF/1617724
US-Rws	2014-2020	Gerald Flerchinger (2021), AmeriFlux BASE US-Rws Reynolds Creek Wyoming big sagebrush, Ver. 4-5, AmeriFlux AMP, (Dataset). https://doi.org/10.17190/AMF/1375201
US-SdH	2004-2009	Dave Billesbach, Tim J. Arkebauer (2016), AmeriFlux BASE US-SdH Nebraska SandHills Dry Valley, Ver. 1-1, AmeriFlux AMP, (Dataset). https://doi.org/10.17190/AMF/1246136
US-Seg	2007-2022	Marcy Litvak (2022), AmeriFlux BASE US-Seg Sevilleta grassland, Ver. 20-5, AmeriFlux AMP, (Dataset). https://doi.org/10.17190/AMF/1246124
US-Ses	2007-2022	Marcy Litvak (2022), AmeriFlux BASE US-Ses Sevilleta shrubland, Ver. 20-5, AmeriFlux AMP, (Dataset). https://doi.org/10.17190/AMF/1246125
US-Slt	2005-2014	Ken Clark (2016), AmeriFlux BASE US-Slt Silas Little- New Jersey, Ver. 5-1, AmeriFlux AMP, (Dataset). https://doi.org/10.17190/AMF/1246096
US-Snd	2007-2014	Matteo Detto, Cove Sturtevant, Patty Oikawa, Joseph Verfaillie, Dennis Baldocchi (2016), AmeriFlux BASE US-Snd Sherman Island, Ver. 2-1, AmeriFlux AMP, (Dataset). https://doi.org/10.17190/AMF/1246094

US-Sne	2016-2020	Robert Shortt, Kyle Hemes, Daphne Szutu, Joseph Verfaillie, Dennis Baldocchi (2021), AmeriFlux BASE US-Sne Sherman Island Restored Wetland, Ver. 7-5, AmeriFlux AMP, (Dataset). https://doi.org/10.17190/AMF/1418684
US-Snf	2018-2020	Kuno Kusak, Camilo Rey Sanchez, Daphne Szutu, Dennis Baldocchi (2020), AmeriFlux BASE US-Snf Sherman Barn, Ver. 3-5, AmeriFlux AMP, (Dataset). https://doi.org/10.17190/AMF/1579718
US-SP1	2000-2011	Tim Martin (2016), AmeriFlux BASE US-SP1 Slashpine-Austin Cary- 65yrs nat regen, Ver. 4-1, AmeriFlux AMP, (Dataset). https://doi.org/10.17190/AMF/1246100
US-SP2	1999-2008	Tim Martin (2016), AmeriFlux BASE US-SP2 Slashpine-Mize-clearcut-3yr,regen, Ver. 3-1, AmeriFlux AMP, (Dataset). https://doi.org/10.17190/AMF/1246101
US-SP3	1999-2010	Tim Martin (2016), AmeriFlux BASE US-SP3 Slashpine-Donaldson-mid-rot- 12yrs, Ver. 3-1, AmeriFlux AMP, (Dataset). https://doi.org/10.17190/AMF/1246102
US-SRC	2008-2014	Shirley Kurc (2019), AmeriFlux BASE US-SRC Santa Rita Creosote, Ver. 6-5, AmeriFlux AMP, (Dataset). https://doi.org/10.17190/AMF/1246127
US-SRG	2008-2022	Russell Scott (2023), AmeriFlux BASE US-SRG Santa Rita Grassland, Ver. 14-5, AmeriFlux AMP, (Dataset). https://doi.org/10.17190/AMF/1246154
US-SRM	2004-2022	Russell Scott (2023), AmeriFlux BASE US-SRM Santa Rita Mesquite, Ver. 25-5, AmeriFlux AMP, (Dataset). https://doi.org/10.17190/AMF/1246104
US-Srr	2014-2017	Brian Bergamaschi, Lisamarie Windham-Myers (2018), AmeriFlux BASE US-Srr Suisun marsh - Rush Ranch, Ver. 1-5, AmeriFlux AMP, (Dataset). https://doi.org/10.17190/AMF/1418685
US-SRS	2011-2018	Enrique R. Vivoni (2022), AmeriFlux BASE US-SRS Santa Rita Savanna, Ver. 3-5, AmeriFlux AMP, (Dataset). https://doi.org/10.17190/AMF/1660351
US-SSH	2016-2021	Kenneth J. Davis (2022), AmeriFlux BASE US-SSH Susquehanna Shale Hills Critical Zone Observatory, Ver. 1-5, AmeriFlux AMP, (Dataset). https://doi.org/10.17190/AMF/1880916
US-StJ	2014-2017	Rodrigo Vargas (2020), AmeriFlux BASE US-StJ St Jones Reserve, Ver. 2-5, AmeriFlux AMP, (Dataset). https://doi.org/10.17190/AMF/1480316
US-SuM	2013-2014	Dong Wang, Ray Anderson (2019), AmeriFlux BASE US-SuM Maui Sugarcane Middle, Ver. 2-5, AmeriFlux AMP, (Dataset). https://doi.org/10.17190/AMF/1246158
US-SuS	2011-2014	Dong Wang, Ray Anderson (2019), AmeriFlux BASE US-SuS Maui Sugarcane Lee/Sheltered, Ver. 2-5, AmeriFlux AMP, (Dataset). https://doi.org/10.17190/AMF/1246159

US-SuW	2011-2013	Dong Wang, Ray Anderson (2019), AmeriFlux BASE US-SuW Maui Sugarcane Windy, Ver. 2-5, AmeriFlux AMP, (Dataset). https://doi.org/10.17190/AMF/1246157
US-Syv	2001-2022	Ankur Desai (2023), AmeriFlux BASE US-Syv Sylvania Wilderness Area, Ver. 23-5, AmeriFlux AMP, (Dataset). https://doi.org/10.17190/AMF/1246106
US-Ton	2001-2022	Siyan Ma, Liukang Xu, Joseph Verfaillie, Dennis Baldocchi (2022), AmeriFlux BASE US-Ton Tonzi Ranch, Ver. 16-5, AmeriFlux AMP, (Dataset). https://doi.org/10.17190/AMF/1245971
US-TrB	2020-2020	Paul Stoy, Ankur Desai, Hilary Dugan, Paul Schramm (2021), AmeriFlux BASE US-TrB Trout Bog, Ver. 1-5, AmeriFlux AMP, (Dataset). https://doi.org/10.17190/AMF/1804493
US-Tw1	2011-2020	Alex Valach, Robert Shortt, Daphne Szutu, Elke Eichelmann, Sara Knox, Kyle Hemes, Joseph Verfaillie, Dennis Baldocchi (2021), AmeriFlux BASE US-Tw1 Twitchell Wetland West Pond, Ver. 9-5, AmeriFlux AMP, (Dataset). https://doi.org/10.17190/AMF/1246147
US-Tw2	2012-2013	Cove Sturtevant, Joseph Verfaillie, Dennis Baldocchi (2019), AmeriFlux BASE US-Tw2 Twitchell Corn, Ver. 2-5, AmeriFlux AMP, (Dataset). https://doi.org/10.17190/AMF/1246148
US-Tw3	2013-2018	Samuel D Chamberlain, Patty Oikawa, Cove Sturtevant, Daphne Szutu, Joseph Verfaillie, Dennis Baldocchi (2018), AmeriFlux BASE US-Tw3 Twitchell Alfalfa, Ver. 5-5, AmeriFlux AMP, (Dataset). https://doi.org/10.17190/AMF/1246149
US-Tw4	2013-2021	Elke Eichelmann, Robert Shortt, Sara Knox, Camilo Rey Sanchez, Alex Valach, Cove Sturtevant, Daphne Szutu, Joseph Verfaillie, Dennis Baldocchi (2021), AmeriFlux BASE US-Tw4 Twitchell East End Wetland, Ver. 12-5, AmeriFlux AMP, (Dataset). https://doi.org/10.17190/AMF/1246151
US-Tw5	2018-2020	Alex Valach, Kuno Kasak, Daphne Szutu, Joseph Verfaillie, Dennis Baldocchi (2020), AmeriFlux BASE US-Tw5 East Pond Wetland, Ver. 3-5, AmeriFlux AMP, (Dataset). https://doi.org/10.17190/AMF/1543380
US-Twt	2009-2017	Sara Knox, Jaclyn Hatala Matthes, Joseph Verfaillie, Dennis Baldocchi (2018), AmeriFlux BASE US-Twt Twitchell Island, Ver. 6-5, AmeriFlux AMP, (Dataset). https://doi.org/10.17190/AMF/1246140
US-Uaf	2003-2022	Masahito Ueyama, Hiroki Iwata, Yoshinobu Harazono (2023), AmeriFlux BASE US-Uaf University of Alaska, Fairbanks, Ver. 11-5, AmeriFlux AMP, (Dataset). https://doi.org/10.17190/AMF/1480322
US-UC1	2019-2022	Sarah Goslee (2023), AmeriFlux BASE US-UC1 LTAR UCB (Upper Chesapeake Bay) EC1, Ver. 2-5, AmeriFlux AMP, (Dataset). https://doi.org/10.17190/AMF/1865482

US-UC2	2019-2022	Sarah Goslee (2023), AmeriFlux BASE US-UC2 LTAR UCB (Upper Chesapeake Bay) EC2, Ver. 2-5, AmeriFlux AMP, (Dataset). https://doi.org/10.17190/AMF/1865483
US-UiA	2008-2016	Bethany Blakely, Caitlin Moore, Carl J Bernacchi, Taylor Pederson (2022), AmeriFlux BASE US-UiA University of Illinois Switchgrass, Ver. 2-5, AmeriFlux AMP, (Dataset). https://doi.org/10.17190/AMF/1617725
US-UiB	2008-2016	Bethany Blakely, Caitlin Moore, Carl J Bernacchi, Taylor Pederson (2022), AmeriFlux BASE US-UiB University of Illinois Miscanthus, Ver. 1-5, AmeriFlux AMP, (Dataset). https://doi.org/10.17190/AMF/1846664
US-UiC	2008-2016	Carl J Bernacchi, Bethany Blakely, Caitlin Moore, Taylor Pederson (2022), AmeriFlux BASE US-UiC University of Illinois Maize-Soy, Ver. 1-5, AmeriFlux AMP, (Dataset). https://doi.org/10.17190/AMF/1846665
US-UM3	2013-2014	Gil Bohrer (2018), AmeriFlux BASE US-UM3 Douglas Lake, Ver. 1-5, AmeriFlux AMP, (Dataset). https://doi.org/10.17190/AMF/1480315
US-UMB	2000-2021	Christopher Gough, Gil Bohrer, Peter Curtis (2022), AmeriFlux BASE US-UMB Univ. of Mich. Biological Station, Ver. 18-5, AmeriFlux AMP, (Dataset). https://doi.org/10.17190/AMF/1246107
US-UMd	2007-2021	Christopher Gough, Gil Bohrer, Peter Curtis (2022), AmeriFlux BASE US-UMd UMBS Disturbance, Ver. 12-5, AmeriFlux AMP, (Dataset). https://doi.org/10.17190/AMF/1246134
US-Var	2000-2022	Siyan Ma, Liukang Xu, Joseph Verfaillie, Dennis Baldocchi (2022), AmeriFlux BASE US-Var Vaira Ranch- Ione, Ver. 18-5, AmeriFlux AMP, (Dataset). https://doi.org/10.17190/AMF/1245984
US-Vcm	2007-2022	Marcy Litvak (2022), AmeriFlux BASE US-Vcm Valles Caldera Mixed Conifer, Ver. 22-5, AmeriFlux AMP, (Dataset). https://doi.org/10.17190/AMF/1246121
US-Vcp	2007-2022	Marcy Litvak (2022), AmeriFlux BASE US-Vcp Valles Caldera Ponderosa Pine, Ver. 19-5, AmeriFlux AMP, (Dataset). https://doi.org/10.17190/AMF/1246122
US-Vcs	2016-2022	Marcy Litvak (2022), AmeriFlux BASE US-Vcs Valles Caldera Sulphur Springs Mixed Conifer, Ver. 12-5, AmeriFlux AMP, (Dataset). https://doi.org/10.17190/AMF/1418681
US-WCr	1998-2022	Ankur Desai (2022), AmeriFlux BASE US-WCr Willow Creek, Ver. 24-5, AmeriFlux AMP, (Dataset). https://doi.org/10.17190/AMF/1246111
US-Whs	2007-2022	Russ Scott (2023), AmeriFlux BASE US-Whs Walnut Gulch Lucky Hills Shrub, Ver. 20-5, AmeriFlux AMP, (Dataset). https://doi.org/10.17190/AMF/1246113
US-Wi0	2002-2002	Jiquan Chen (2020), AmeriFlux BASE US-Wi0 Young red pine (YRP), Ver. 3-5, AmeriFlux AMP, (Dataset). https://doi.org/10.17190/AMF/1246016

US-Wi1	2003-2003	Jiquan Chen (2020), AmeriFlux BASE US-Wi1 Intermediate hardwood (IHW), Ver. 3-5, AmeriFlux AMP, (Dataset). https://doi.org/10.17190/AMF/1246015
US-Wi3	2002-2004	Jiquan Chen (2020), AmeriFlux BASE US-Wi3 Mature hardwood (MHW), Ver. 3-5, AmeriFlux AMP, (Dataset). https://doi.org/10.17190/AMF/1246018
US-Wi4	2002-2005	Jiquan Chen (2020), AmeriFlux BASE US-Wi4 Mature red pine (MRP), Ver. 3-5, AmeriFlux AMP, (Dataset). https://doi.org/10.17190/AMF/1246019
US-Wi5	2004-2004	Jiquan Chen (2020), AmeriFlux BASE US-Wi5 Mixed young jack pine (MYJP), Ver. 3-5, AmeriFlux AMP, (Dataset). https://doi.org/10.17190/AMF/1246020
US-Wi6	2002-2003	Jiquan Chen (2020), AmeriFlux BASE US-Wi6 Pine barrens #1 (PB1), Ver. 3-5, AmeriFlux AMP, (Dataset). https://doi.org/10.17190/AMF/1246021
US-Wi7	2005-2005	Jiquan Chen (2020), AmeriFlux BASE US-Wi7 Red pine clearcut (RPCC), Ver. 3-5, AmeriFlux AMP, (Dataset). https://doi.org/10.17190/AMF/1246022
US-Wi8	2002-2002	Jiquan Chen (2020), AmeriFlux BASE US-Wi8 Young hardwood clearcut (YHW), Ver. 3-5, AmeriFlux AMP, (Dataset). https://doi.org/10.17190/AMF/1246023
US-Wi9	2004-2005	Jiquan Chen (2020), AmeriFlux BASE US-Wi9 Young Jack pine (YJP), Ver. 3-5, AmeriFlux AMP, (Dataset). https://doi.org/10.17190/AMF/1246024
US-Wjs	2007-2022	Marcy Litvak (2022), AmeriFlux BASE US-Wjs Willard Juniper Savannah, Ver. 19-5, AmeriFlux AMP, (Dataset). https://doi.org/10.17190/AMF/1246120
US-Wkg	2004-2022	Russell Scott (2023), AmeriFlux BASE US-Wkg Walnut Gulch Kendall Grasslands, Ver. 20-5, AmeriFlux AMP, (Dataset). https://doi.org/10.17190/AMF/1246112
US-Wlr	2001-2004	David Cook, Richard L. Coulter (2018), AmeriFlux BASE US-Wlr Walnut River Watershed (Smileyburg), Ver. 4-5, AmeriFlux AMP, (Dataset). https://doi.org/10.17190/AMF/1246115
US-WPT	2011-2013	Jiquan Chen, Housen Chu (2019), AmeriFlux BASE US-WPT Winous Point North Marsh, Ver. 4-5, AmeriFlux AMP, (Dataset). https://doi.org/10.17190/AMF/1246155
US-Wrc	1998-2015	Sonia Wharton (2016), AmeriFlux BASE US-Wrc Wind River Crane Site, Ver. 8-1, AmeriFlux AMP, (Dataset). https://doi.org/10.17190/AMF/1246114
US-xAB	2017-2022	NEON (National Ecological Observatory Network) (2022), AmeriFlux BASE US-xAB NEON Abby Road (ABBY), Ver. 6-5, AmeriFlux AMP, (Dataset). https://doi.org/10.17190/AMF/1617726
US-xAE	2017-2022	NEON (National Ecological Observatory Network) (2022), AmeriFlux BASE US-xAE NEON Klemme Range Research Station (OAES), Ver. 5-5, AmeriFlux AMP, (Dataset). https://doi.org/10.17190/AMF/1671891

US-xBA	2017-2022	NEON (National Ecological Observatory Network) (2022), AmeriFlux BASE US-xBA NEON Barrow Environmental Observatory (BARR), Ver. 5-5, AmeriFlux AMP, (Dataset). https://doi.org/10.17190/AMF/1671892
US-xBL	2017-2022	NEON (National Ecological Observatory Network) (2022), AmeriFlux BASE US-xBL NEON Blandy Experimental Farm (BLAN), Ver. 5-5, AmeriFlux AMP, (Dataset). https://doi.org/10.17190/AMF/1671893
US-xBN	2017-2022	NEON (National Ecological Observatory Network) (2022), AmeriFlux BASE US-xBN NEON Caribou Creek - Poker Flats Watershed (BONA), Ver. 6-5, AmeriFlux AMP, (Dataset). https://doi.org/10.17190/AMF/1617727
US-xBR	2017-2022	NEON (National Ecological Observatory Network) (2022), AmeriFlux BASE US-xBR NEON Bartlett Experimental Forest (BART), Ver. 6-5, AmeriFlux AMP, (Dataset). https://doi.org/10.17190/AMF/1579542
US-xCL	2017-2022	NEON (National Ecological Observatory Network) (2022), AmeriFlux BASE US-xCL NEON LBJ National Grassland (CLBJ), Ver. 5-5, AmeriFlux AMP, (Dataset). https://doi.org/10.17190/AMF/1671894
US-xCP	2016-2022	NEON (National Ecological Observatory Network) (2022), AmeriFlux BASE US-xCP NEON Central Plains Experimental Range (CPER), Ver. 6-5, AmeriFlux AMP, (Dataset). https://doi.org/10.17190/AMF/1579720
US-xDC	2017-2022	NEON (National Ecological Observatory Network) (2022), AmeriFlux BASE US-xDC NEON Dakota Coteau Field School (DCFS), Ver. 6-5, AmeriFlux AMP, (Dataset). https://doi.org/10.17190/AMF/1617728
US-xDJ	2017-2022	NEON (National Ecological Observatory Network) (2022), AmeriFlux BASE US-xDJ NEON Delta Junction (DEJU), Ver. 6-5, AmeriFlux AMP, (Dataset). https://doi.org/10.17190/AMF/1634884
US-xDL	2017-2022	NEON (National Ecological Observatory Network) (2022), AmeriFlux BASE US-xDL NEON Dead Lake (DELA), Ver. 6-5, AmeriFlux AMP, (Dataset). https://doi.org/10.17190/AMF/1579721
US-xDS	2017-2022	NEON (National Ecological Observatory Network) (2022), AmeriFlux BASE US-xDS NEON Disney Wilderness Preserve (DSNY), Ver. 5-5, AmeriFlux AMP, (Dataset). https://doi.org/10.17190/AMF/1671895
US-xGR	2017-2022	NEON (National Ecological Observatory Network) (2022), AmeriFlux BASE US-xGR NEON Great Smoky Mountains National Park, Twin Creeks (GRSM), Ver. 6-5, AmeriFlux AMP, (Dataset). https://doi.org/10.17190/AMF/1634885
US-xHA	2017-2022	NEON (National Ecological Observatory Network) (2022), AmeriFlux BASE US-xHA NEON Harvard Forest (HARV), Ver. 7-5, AmeriFlux AMP, (Dataset). https://doi.org/10.17190/AMF/1562391

US-xHE	2017-2022	NEON (National Ecological Observatory Network) (2022), AmeriFlux BASE US-xHE NEON Healy (HEAL), Ver. 6-5, AmeriFlux AMP, (Dataset). https://doi.org/10.17190/AMF/1617729
US-xJE	2017-2022	NEON (National Ecological Observatory Network) (2022), AmeriFlux BASE US-xJE NEON Jones Ecological Research Center (JERC), Ver. 6-5, AmeriFlux AMP, (Dataset). https://doi.org/10.17190/AMF/1617730
US-xJR	2017-2022	NEON (National Ecological Observatory Network) (2022), AmeriFlux BASE US-xJR NEON Jornada LTER (JORN), Ver. 6-5, AmeriFlux AMP, (Dataset). https://doi.org/10.17190/AMF/1617731
US-xKA	2017-2022	NEON (National Ecological Observatory Network) (2022), AmeriFlux BASE US-xKA NEON Konza Prairie Biological Station - Relocatable (KONA), Ver. 6-5, AmeriFlux AMP, (Dataset). https://doi.org/10.17190/AMF/1579722
US-xKZ	2017-2022	NEON (National Ecological Observatory Network) (2022), AmeriFlux BASE US-xKZ NEON Konza Prairie Biological Station (KONZ), Ver. 7-5, AmeriFlux AMP, (Dataset). https://doi.org/10.17190/AMF/1562392
US-xLE	2017-2022	NEON (National Ecological Observatory Network) (2022), AmeriFlux BASE US-xLE NEON Lenoir Landing (LENO), Ver. 4-5, AmeriFlux AMP, (Dataset). https://doi.org/10.17190/AMF/1773398
US-xMB	2017-2022	NEON (National Ecological Observatory Network) (2022), AmeriFlux BASE US-xMB NEON Moab (MOAB), Ver. 5-5, AmeriFlux AMP, (Dataset). https://doi.org/10.17190/AMF/1671896
US-xML	2017-2022	NEON (National Ecological Observatory Network) (2022), AmeriFlux BASE US-xML NEON Mountain Lake Biological Station (MLBS), Ver. 5-5, AmeriFlux AMP, (Dataset). https://doi.org/10.17190/AMF/1671897
US-xNG	2017-2022	NEON (National Ecological Observatory Network) (2022), AmeriFlux BASE US-xNG NEON Northern Great Plains Research Laboratory (NOGP), Ver. 6-5, AmeriFlux AMP, (Dataset). https://doi.org/10.17190/AMF/1617732
US-xNQ	2017-2022	NEON (National Ecological Observatory Network) (2022), AmeriFlux BASE US-xNQ NEON Onaqui-Ault (ONAQ), Ver. 6-5, AmeriFlux AMP, (Dataset). https://doi.org/10.17190/AMF/1617733
US-xNW	2017-2022	NEON (National Ecological Observatory Network) (2022), AmeriFlux BASE US-xNW NEON Niwot Ridge Mountain Research Station (NIWO), Ver. 5-5, AmeriFlux AMP, (Dataset). https://doi.org/10.17190/AMF/1671898
US-xPU	2019-2022	NEON (National Ecological Observatory Network) (2022), AmeriFlux BASE US-xPU NEON Pu’u Maka’ala Natural Area Reserve (PUUM), Ver. 4-5, AmeriFlux AMP, (Dataset). https://doi.org/10.17190/AMF/1773399

US-xRM	2017-2022	NEON (National Ecological Observatory Network) (2022), AmeriFlux BASE US-xRM NEON Rocky Mountain National Park, CASTNET (RMNP), Ver. 6-5, AmeriFlux AMP, (Dataset). https://doi.org/10.17190/AMF/1579723
US-xRN	2017-2022	NEON (National Ecological Observatory Network) (2022), AmeriFlux BASE US-xRN NEON Oak Ridge National Lab (ORNL), Ver. 4-5, AmeriFlux AMP, (Dataset). https://doi.org/10.17190/AMF/1773400
US-xSB	2017-2022	NEON (National Ecological Observatory Network) (2022), AmeriFlux BASE US-xSB NEON Ordway-Swisher Biological Station (OSBS), Ver. 5-5, AmeriFlux AMP, (Dataset). https://doi.org/10.17190/AMF/1671899
US-xSC	2016-2022	NEON (National Ecological Observatory Network) (2022), AmeriFlux BASE US-xSC NEON Smithsonian Conservation Biology Institute (SCBI), Ver. 5-5, AmeriFlux AMP, (Dataset). https://doi.org/10.17190/AMF/1671900
US-xSE	2017-2022	NEON (National Ecological Observatory Network) (2022), AmeriFlux BASE US-xSE NEON Smithsonian Environmental Research Center (SERC), Ver. 6-5, AmeriFlux AMP, (Dataset). https://doi.org/10.17190/AMF/1617734
US-xSJ	2018-2022	NEON (National Ecological Observatory Network) (2022), AmeriFlux BASE US-xSJ NEON San Joaquin Experimental Range (SJER), Ver. 5-5, AmeriFlux AMP, (Dataset). https://doi.org/10.17190/AMF/1671901
US-xSL	2017-2022	NEON (National Ecological Observatory Network) (2022), AmeriFlux BASE US-xSL NEON North Sterling, CO (STER), Ver. 6-5, AmeriFlux AMP, (Dataset). https://doi.org/10.17190/AMF/1617735
US-xSP	2017-2022	NEON (National Ecological Observatory Network) (2022), AmeriFlux BASE US-xSP NEON Soaproot Saddle (SOAP), Ver. 6-5, AmeriFlux AMP, (Dataset). https://doi.org/10.17190/AMF/1617736
US-xSR	2017-2022	NEON (National Ecological Observatory Network) (2022), AmeriFlux BASE US-xSR NEON Santa Rita Experimental Range (SRER), Ver. 6-5, AmeriFlux AMP, (Dataset). https://doi.org/10.17190/AMF/1579543
US-xST	2017-2022	NEON (National Ecological Observatory Network) (2022), AmeriFlux BASE US-xST NEON Steigerwaldt Land Services (STEI), Ver. 6-5, AmeriFlux AMP, (Dataset). https://doi.org/10.17190/AMF/1617737
US-xTA	2017-2022	NEON (National Ecological Observatory Network) (2022), AmeriFlux BASE US-xTA NEON Talladega National Forest (TALL), Ver. 5-5, AmeriFlux AMP, (Dataset). https://doi.org/10.17190/AMF/1671902
US-xTE	2018-2022	NEON (National Ecological Observatory Network) (2022), AmeriFlux BASE US-xTE NEON Lower Teakettle (TEAK), Ver. 6-5, AmeriFlux AMP, (Dataset). https://doi.org/10.17190/AMF/1617738

US-xTL	2017-2022	NEON (National Ecological Observatory Network) (2022), AmeriFlux BASE US-xTL NEON Toolik (TOOL), Ver. 6-5, AmeriFlux AMP, (Dataset). https://doi.org/10.17190/AMF/1617739
US-xTR	2017-2022	NEON (National Ecological Observatory Network) (2022), AmeriFlux BASE US-xTR NEON Treehaven (TREE), Ver. 6-5, AmeriFlux AMP, (Dataset). https://doi.org/10.17190/AMF/1634886
US-xUK	2017-2022	NEON (National Ecological Observatory Network) (2022), AmeriFlux BASE US-xUK NEON The University of Kansas Field Station (UKFS), Ver. 6-5, AmeriFlux AMP, (Dataset). https://doi.org/10.17190/AMF/1617740
US-xUN	2017-2022	NEON (National Ecological Observatory Network) (2022), AmeriFlux BASE US-xUN NEON University of Notre Dame Environmental Research Center (UNDE), Ver. 6-5, AmeriFlux AMP, (Dataset). https://doi.org/10.17190/AMF/1617741
US-xWD	2017-2022	NEON (National Ecological Observatory Network) (2022), AmeriFlux BASE US-xWD NEON Woodworth (WOOD), Ver. 6-5, AmeriFlux AMP, (Dataset). https://doi.org/10.17190/AMF/1579724
US-xWR	2018-2022	NEON (National Ecological Observatory Network) (2022), AmeriFlux BASE US-xWR NEON Wind River Experimental Forest (WREF), Ver. 6-5, AmeriFlux AMP, (Dataset). https://doi.org/10.17190/AMF/1617742
US-xYE	2018-2022	NEON (National Ecological Observatory Network) (2022), AmeriFlux BASE US-xYE NEON Yellowstone Northern Range (Frog Rock) (YELL), Ver. 6-5, AmeriFlux AMP, (Dataset). https://doi.org/10.17190/AMF/1617743

Supplementary Figures

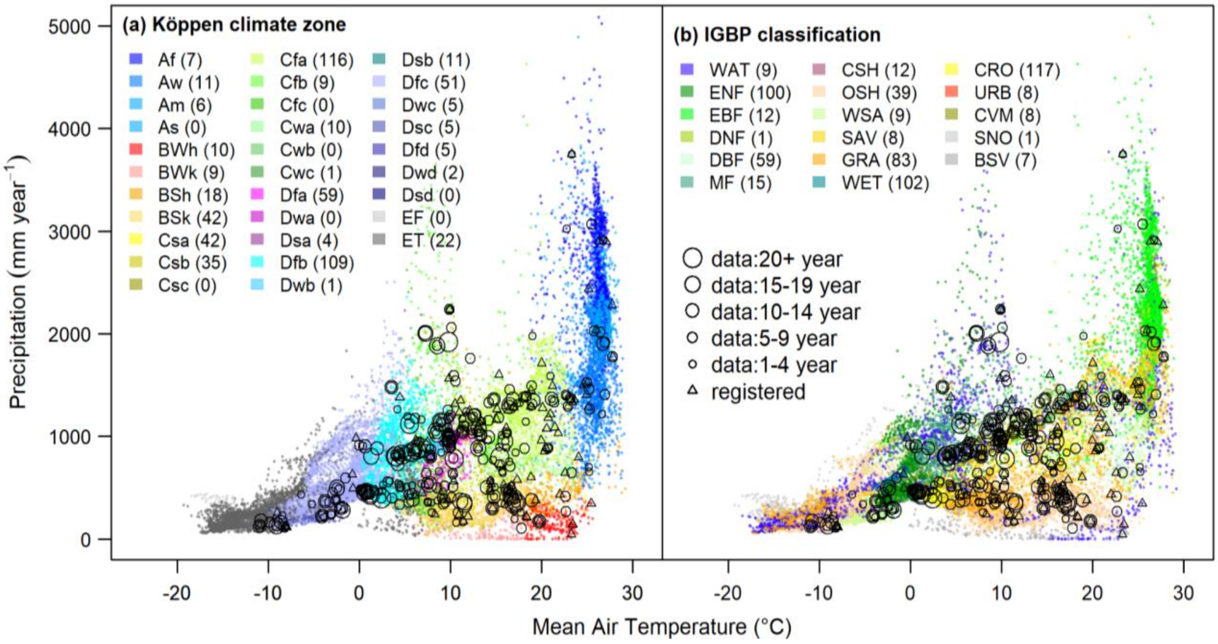

Supplementary Figure S1. Distribution of AmeriFlux sites by mean annual temperature and precipitation grouped by (a) Köppen climate zones and (b) International Geosphere-Biosphere Programme (IGBP) classifications. Tower locations are shown as triangles (registered sites) and circles (sites with data available on AmeriFlux). The circles' size indicates the length of the data record. Background dots represent annual mean temperature and total precipitation across the Americas using the Climatic Research Unit (CRU) time series (TS) 4.05 gridded dataset from 1981 to 2020 (0.5*0.5 degree)[8]. The colors indicate the Köppen climate zones and IGBP classifications. The numbers in parentheses show the number of sites in each group.

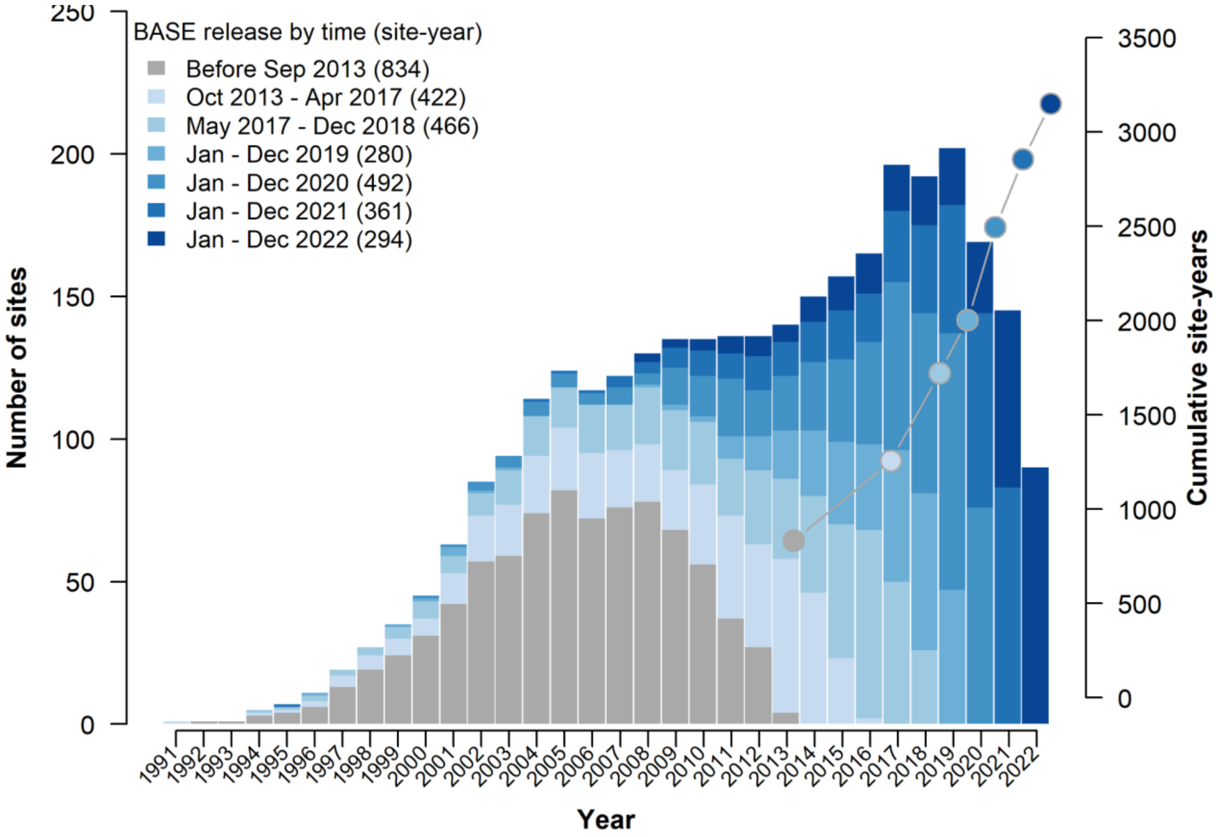

Supplementary Figure S2. AmeriFlux BASE data availability by the time periods published. Bars show the number of sites available for each year (left y-axis). Circles show the cumulative site-years available at the selected time (right y-axis). Color tones indicate the time periods during which the data are published. Key milestones include the onset of the AmeriFlux Management Project (October 2013) and the implementation of the BASE pipeline (May, 2017).

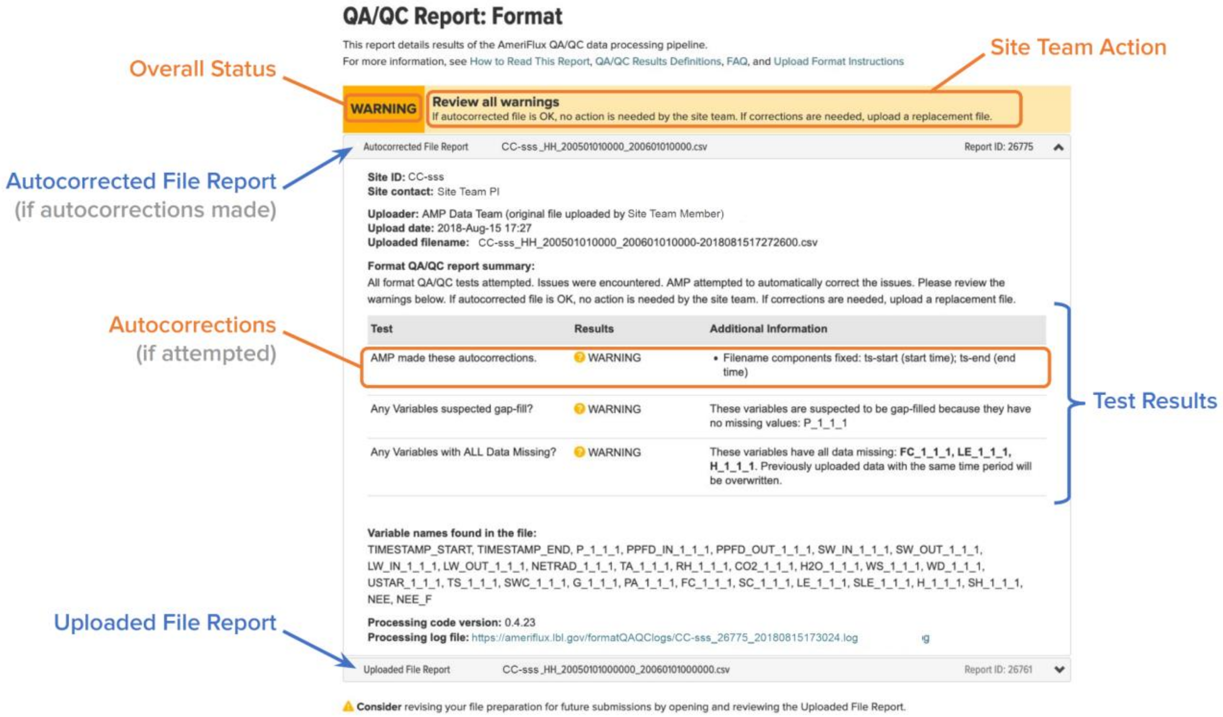

Supplementary Figure S3. An example of the Format QA/QC report. The overall status (i.e., PASS, WARNING, FAIL) is reported with detailed test results for uploaded and autocorrected (if generated) files.

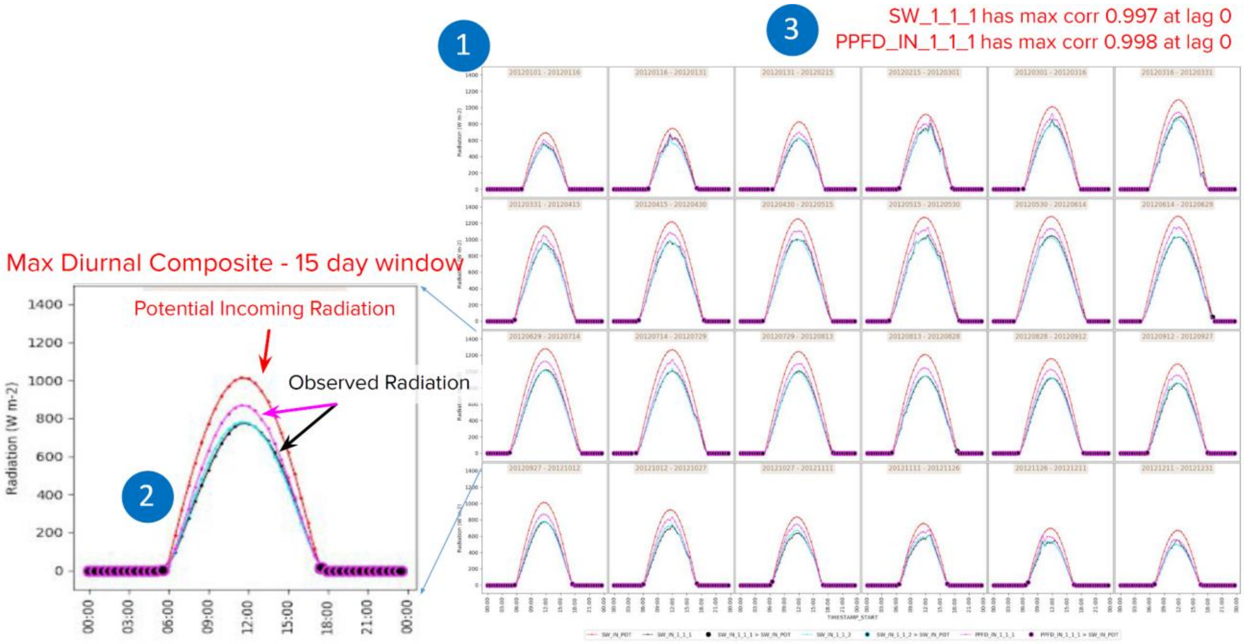

Supplementary Figure S4. Example figure with no issues identified by the timestamp alignment module. The "maximum diurnal composite" is calculated for each 15-day non-overlapping window within a year (1). The module expects that the diurnal patterns align in time between potential incoming radiation (SW_IN_POT) and observed shortwave incoming radiation (SW_IN) and photosynthetic photon flux density (PPFD_IN) as indicated by cross-correlation (3) and that SW_IN and PPFD_IN do not exceed SW_IN_POT in most periods (2).

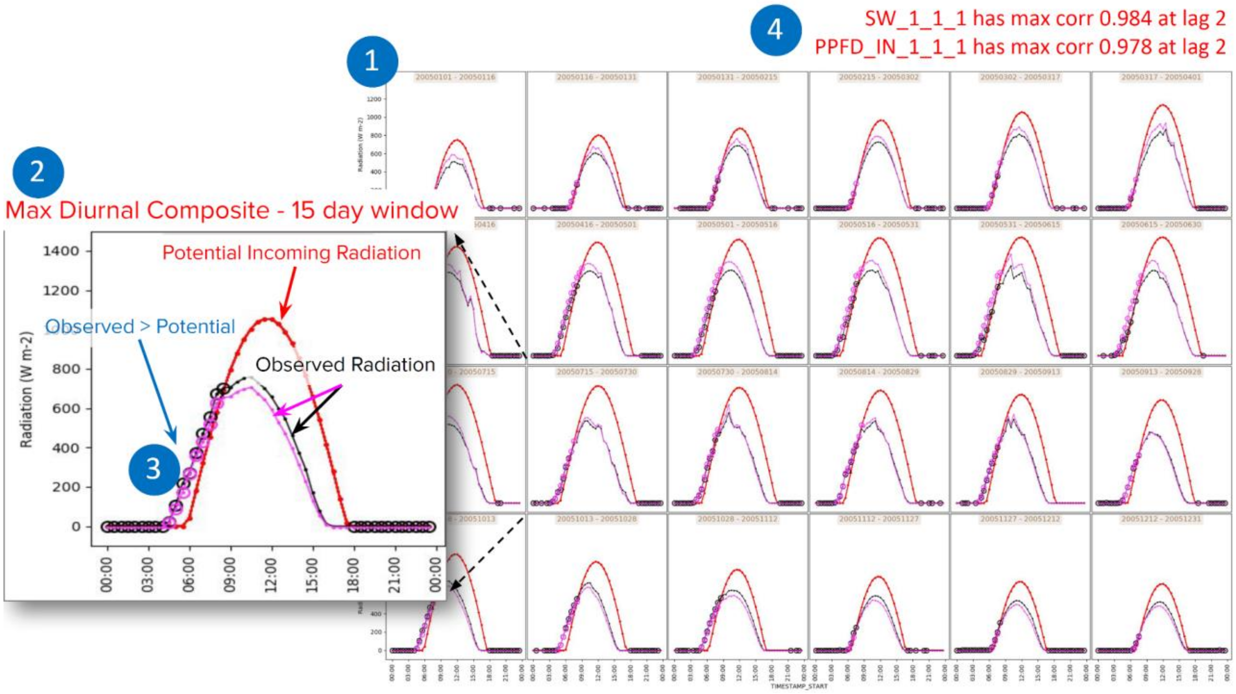

Supplementary Figure S5. Example figure illustrating the timestamp alignment module with detection of potential issues (1). This highlighted example (2-3) shows several occasions that observed shortwave incoming radiation (SW_IN) and photosynthetic photon flux density (PPFD_IN) exceeds the potential incoming radiation (SW_IN_POT), i.e., earlier sunrise/sunset than expected. And (4) the maximum cross-correlation between calculated and observed radiation occurs at a two-step lag, i.e., a one-hour shift for 30-minute data.

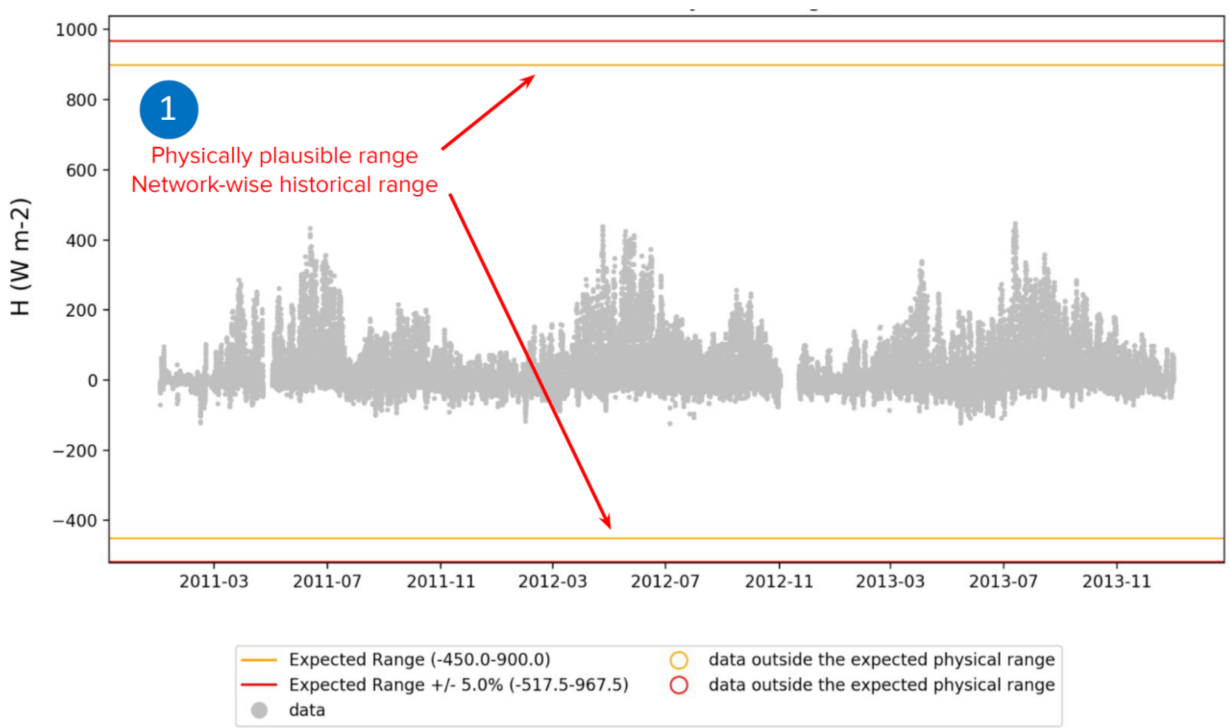

Supplementary Figure S6. Example figure of sensible heat flux (H) with no issues detected by the physical range module. (1) The yellow lines indicate the accepted range defined based on the physically plausible range and the network-wide historical range. The red lines indicate the accepted range plus a ±5% buffer range. In this case, no data points were beyond the plausible ranges.

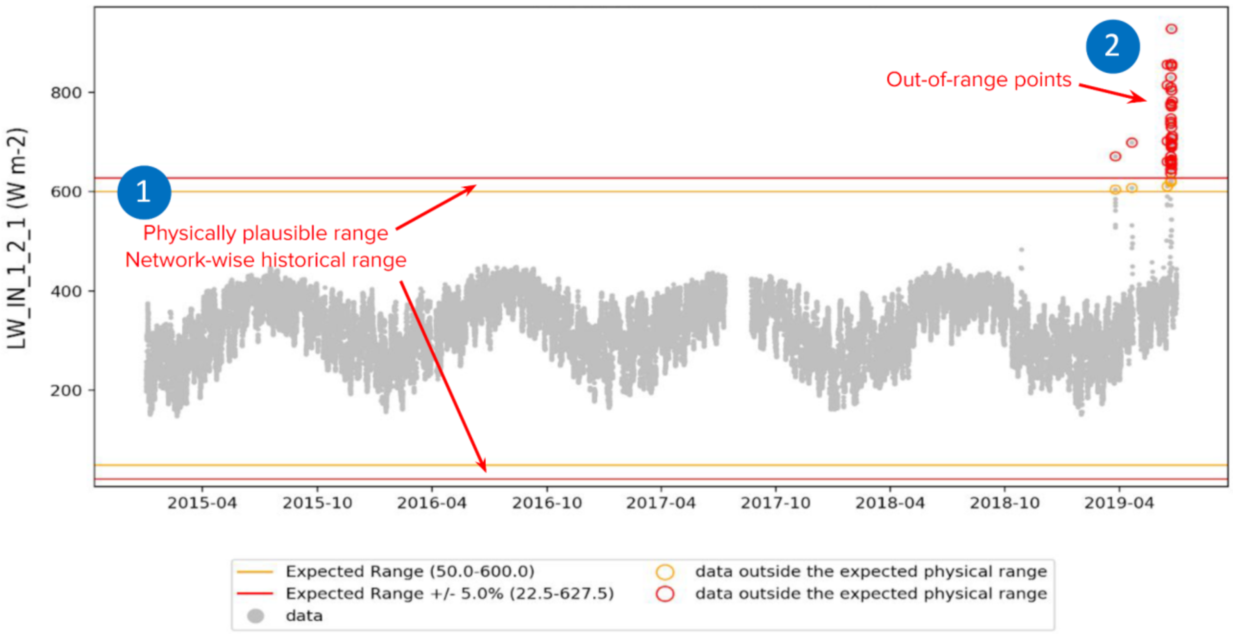

Supplementary Figure S7. Example figure of incoming longwave radiation (LW_IN) illustrating the physical range module. (1) The yellow lines indicate the accepted range defined based on the physically plausible range or the reported network-wide historical range. The red lines indicate the accepted range plus a 5% buffer range. (2) The out-of-range data points (highlighted by red or yellow circles) are detected based on the accepted range. This case has ~0.2% of data points beyond the accepted range plus a ±5% buffer.

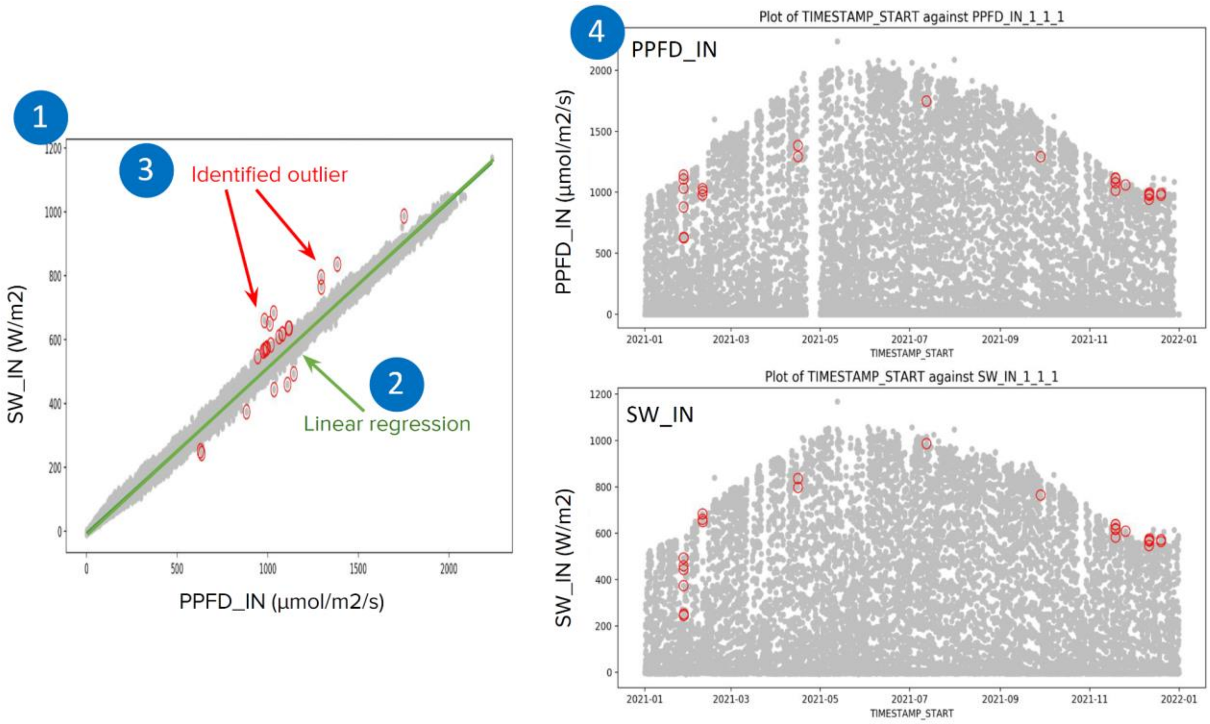

Supplementary Figure S8. Example figure with no issues identified by the multivariate comparison module. The right panel (4) shows a one-year time series of shortwave incoming radiation (SW_IN) and photosynthetic photon flux density (PPFD_IN). The left panel (1) shows the scatter plot of SW_IN and PPFD_IN from the same one-year period. The green line denotes the linear regression generated from all data (2), while the red, highlighted circles denote data points that are flagged as potential outliers based on the distance from the regression line (3). It is common that a few data points are flagged as potential outliers, considering the random measurement errors and stochastic nature.

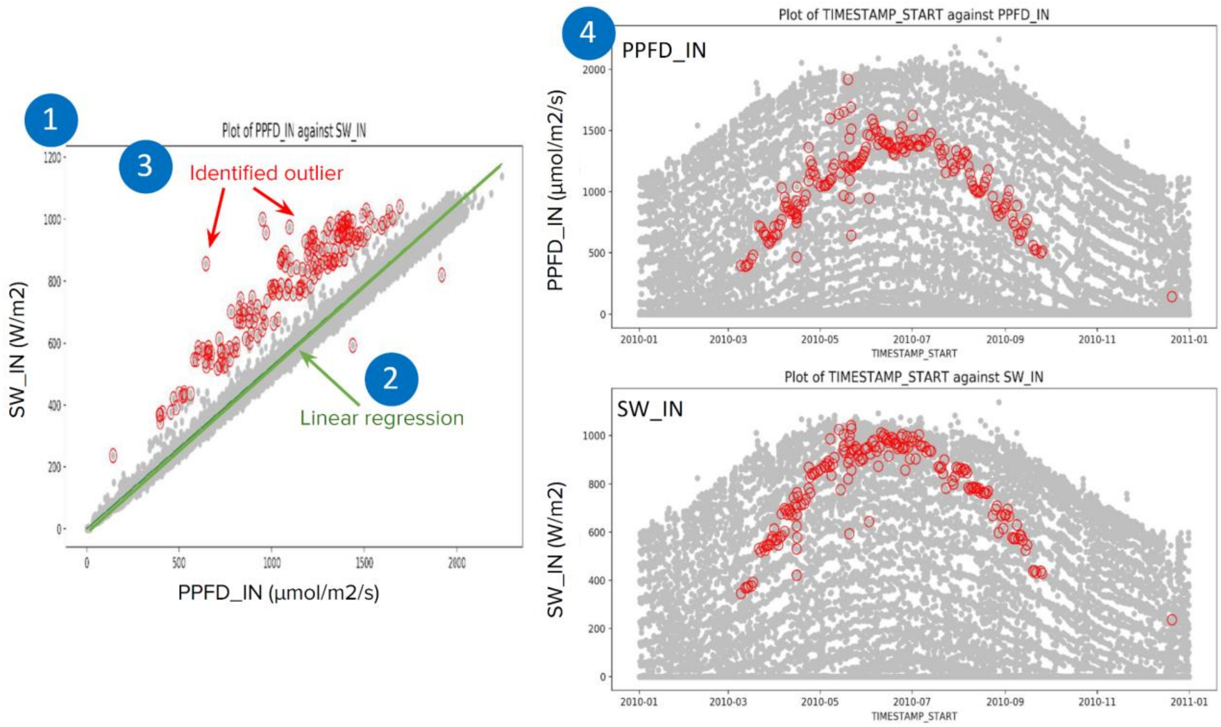

Supplementary Figure S9. Example figure illustrating the multivariate comparison in a year. The right panel (4) shows a one-year time series of shortwave incoming radiation (SW_IN) and photosynthetic photon flux density (PPFD_IN). The left panel (1) shows the scatter plot of SW_IN and PPFD_IN from the same one-year period. The green line denotes the linear regression generated from all data (2), while the red, highlighted circles denote data points flagged as outliers based on distance from the regression line (3). This case has a slightly higher percentage (> 1%) of flagged data points. And the periodic occurrence of flagged outliers suggests one of the radiation sensors, PPFD_IN in this case, is shaded periodically when the other sensor is not.

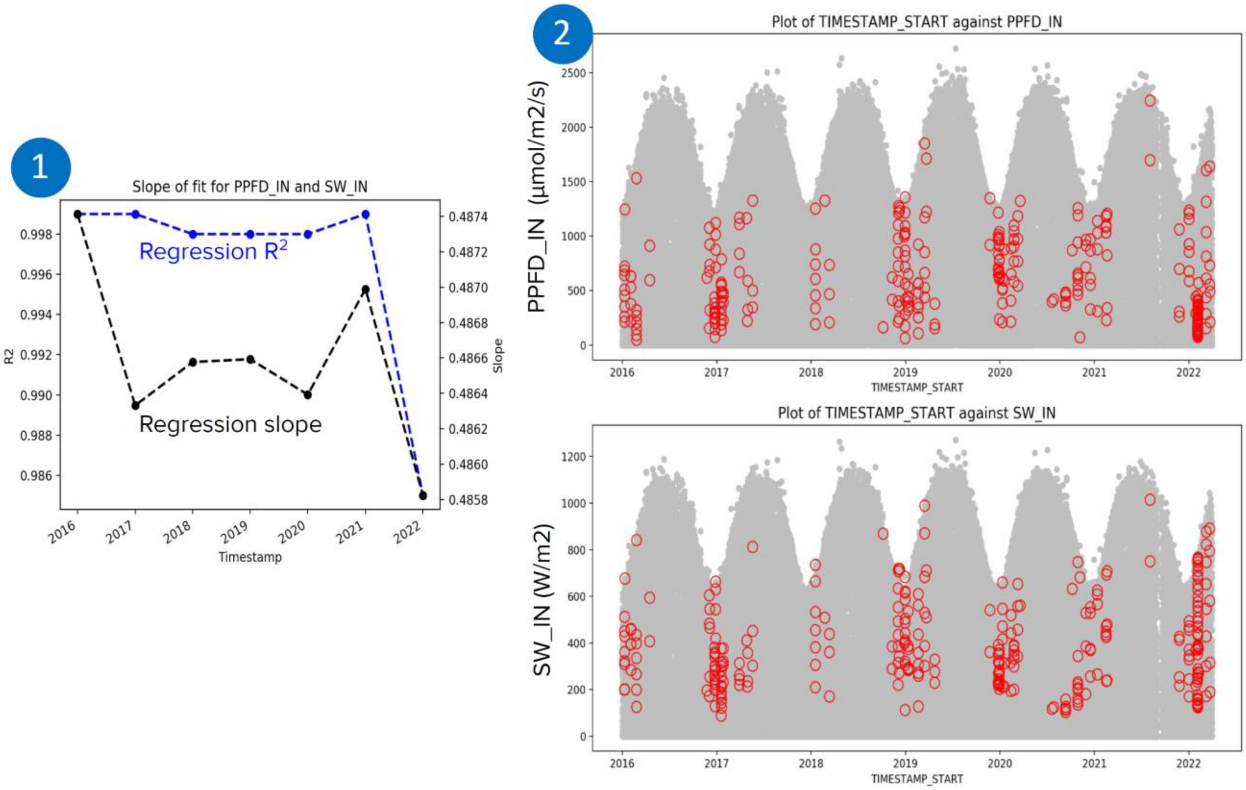

Supplementary Figure S10. Example figure with no issues detected by the multivariate comparison module over multiple years. The right panel (2) shows a 6-year time series of shortwave incoming radiation (SW_IN) and photosynthetic photon flux density (PPFD_IN). The left panel (1) shows the time series of regression slopes and R^2 calculated between PPFD_IN and SW_IN each year (as shown in Supplementary Figures S8-S9). The regression slopes were relatively stable (±0.01) over the years, suggesting no evident shift or trend in either sensor.

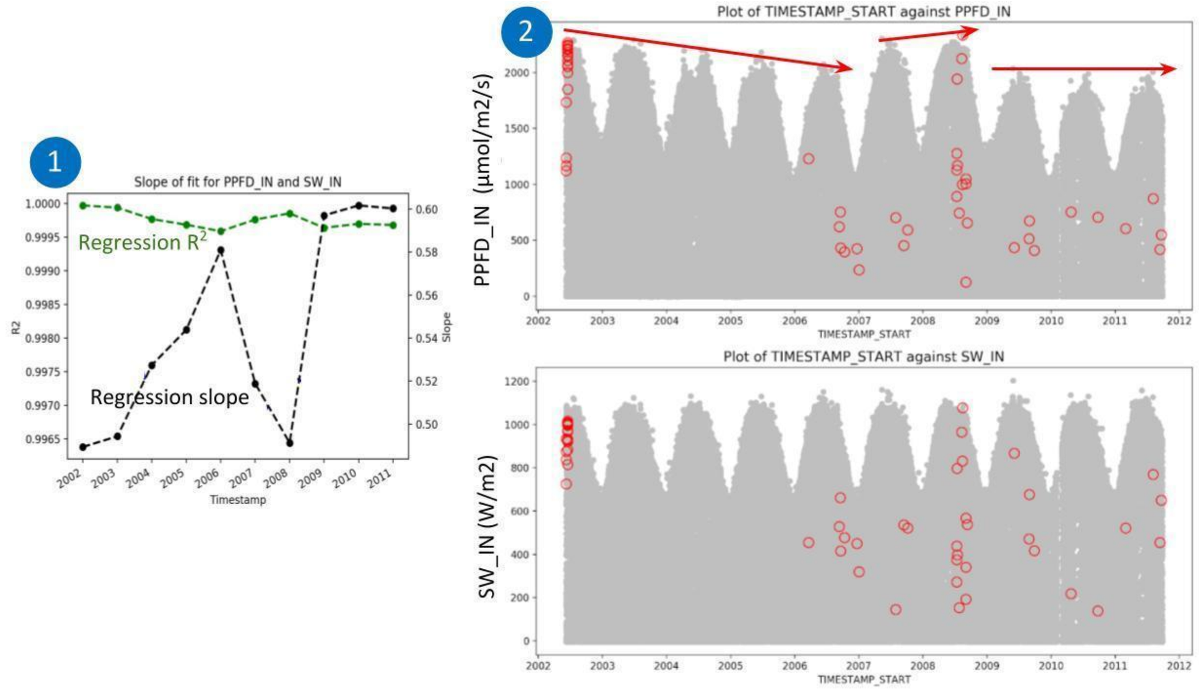

Supplementary Figure S11. Example figure illustrating the multivariate comparison over multiple years. The right panel (2) shows a 10-year time series of shortwave incoming radiation (SW_IN) and photosynthetic photon flux density (PPFD_IN). The left panel (1) shows the time series of regression slopes and R^2 calculated between PPFD_IN and SW_IN each year (as shown in the previous figure). The changes in regression slopes over the years suggest one of the radiation sensors, PPFD_IN in this case, has shifted over the years (red arrows) compared with the other sensor.

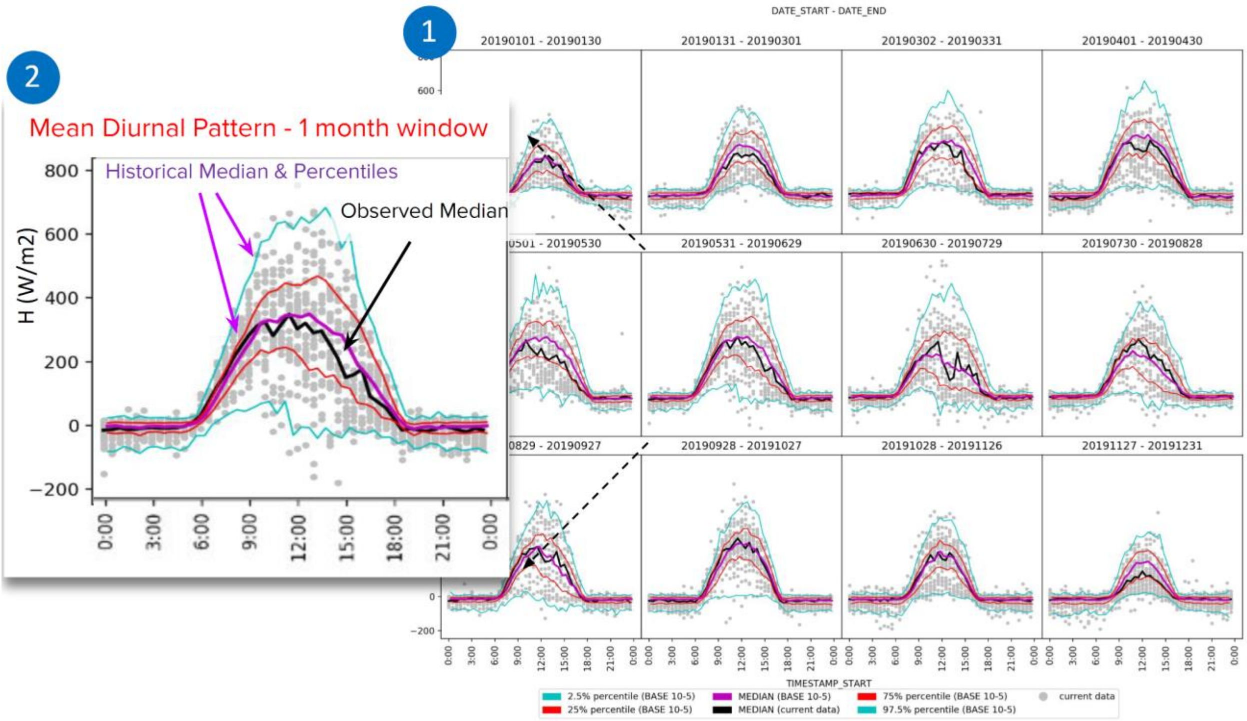

Supplementary Figure S12. Example figure of sensible heat flux (H) with no issues detected by the diurnal-seasonal pattern module. (1) The right panel shows the monthly diurnal plots for a one-year time series of sensible heat flux, including newly submitted data (gray data points and the black median line) and historical ranges (2.5th, 25th, 50th, 75th, 97.5th percentiles in colored lines). Panel (2) shows a monthly example. This case shows that most new data points are within the historical 2.5th-97.5th percentile range and have similar median diurnal patterns to the historical records.

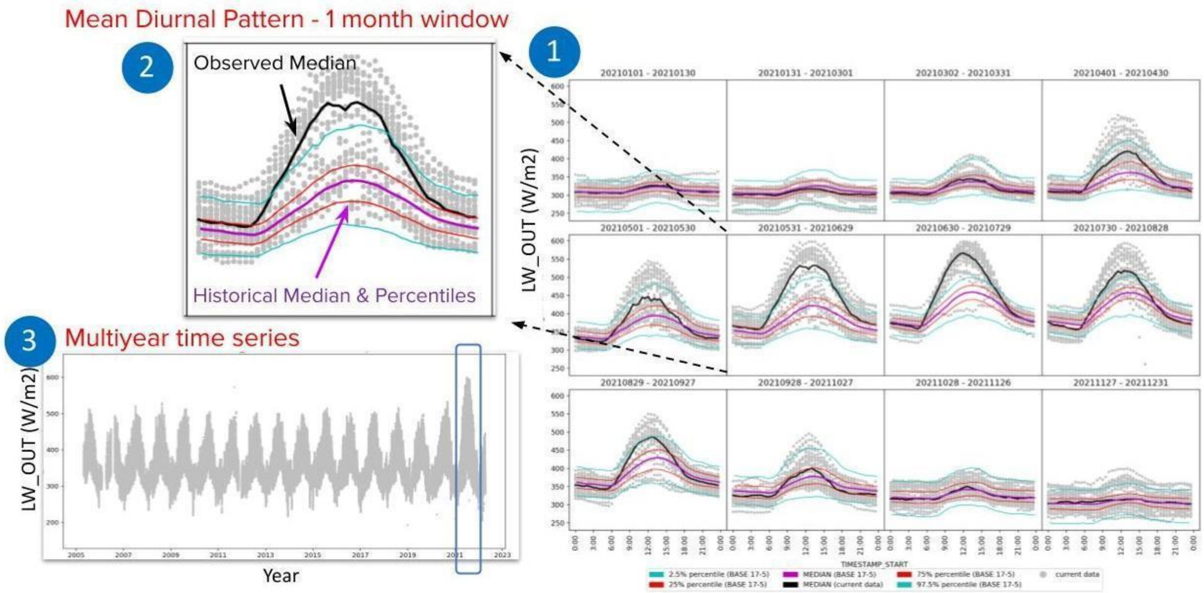

Supplementary Figure S13. Example figure illustrating the diurnal-seasonal pattern check. (1) The right panel shows the monthly diurnal plots for a one-year time series of outgoing longwave radiation (LW_OUT), including newly submitted data (gray data points and the black median line) and historical ranges (2.5th, 25th, 50th, 75th, 97.5th percentiles in colored lines). Panel (2) shows a monthly example. Panel (3) shows the multi-year time series, with the box highlighting the one year in the right panel. The example year has relatively higher LW_OUT readings than previous years (more gray points beyond the historical ranges in (1) and (2)).

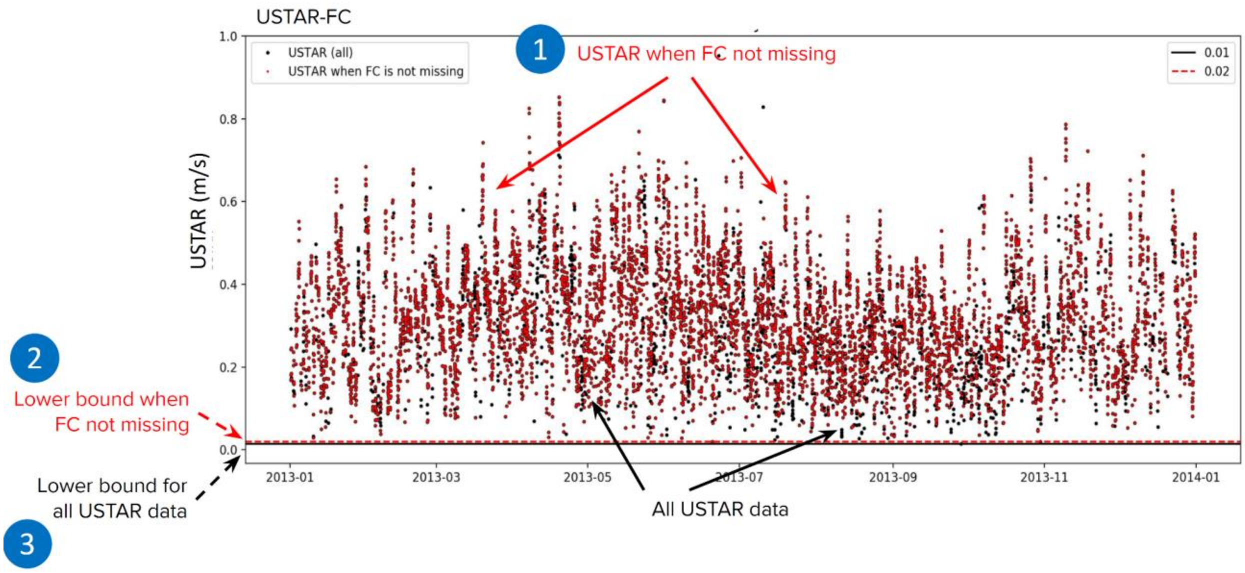

Supplementary Figure S14. Example figure with no issues detected by the USTAR filtering module. This example shows the lower bound of friction velocity (USTAR) when concurrent CO_2 flux (FC) is not missing (red dashed line) is equal or close to the lower bound from all USTAR data (solid black line).

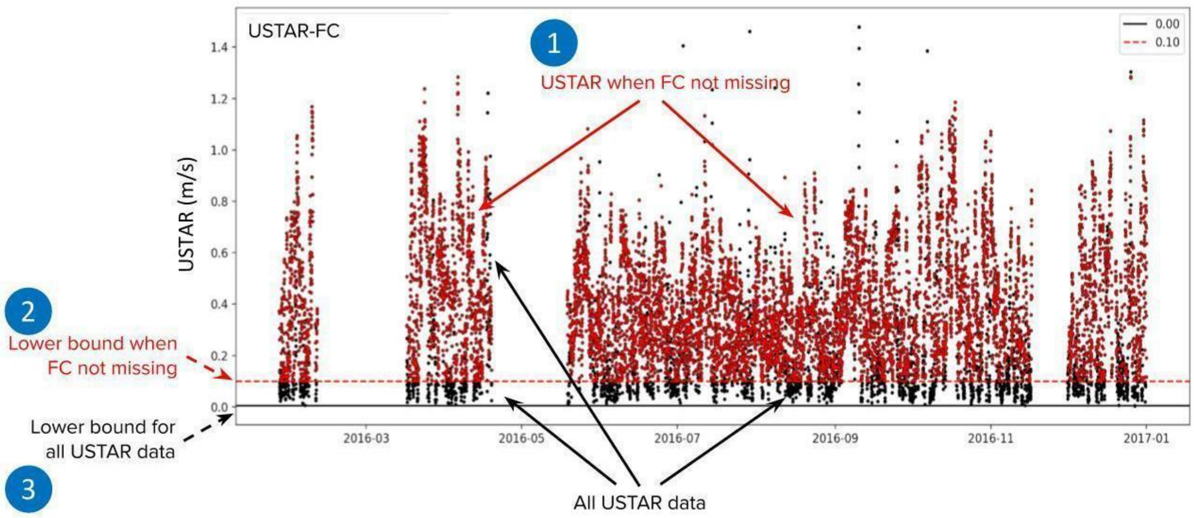

Supplementary Figure S15. Example figure illustrating the USTAR filtering module with detection of potential issues (1). This example shows (2) the lower bound of friction velocity (USTAR) when concurrent CO_2 flux (FC) is not missing (red dashed line) is higher than (3) the lower bound from all USTAR data (black solid line). The difference indicates that the FC data are filtered by using a USTAR threshold of 0.1 m s^{-1}.

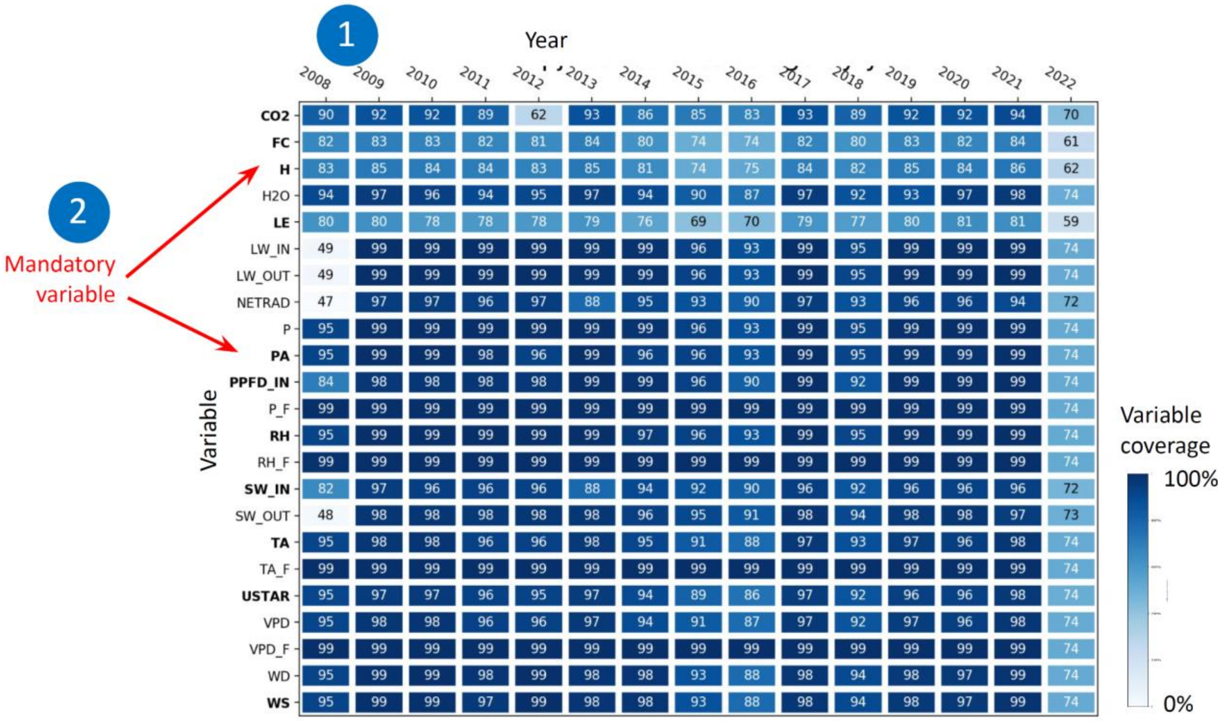

Supplementary Figure S16. Example figure with no issues detected by the variable coverage module. The figure shows the variable coverage (color gradient) by year. The example shows all measurements are present in all years (1). Bolded texts in variable names denote mandatory variables required for further ONEFlux processing to generate the FLUXNET data product.

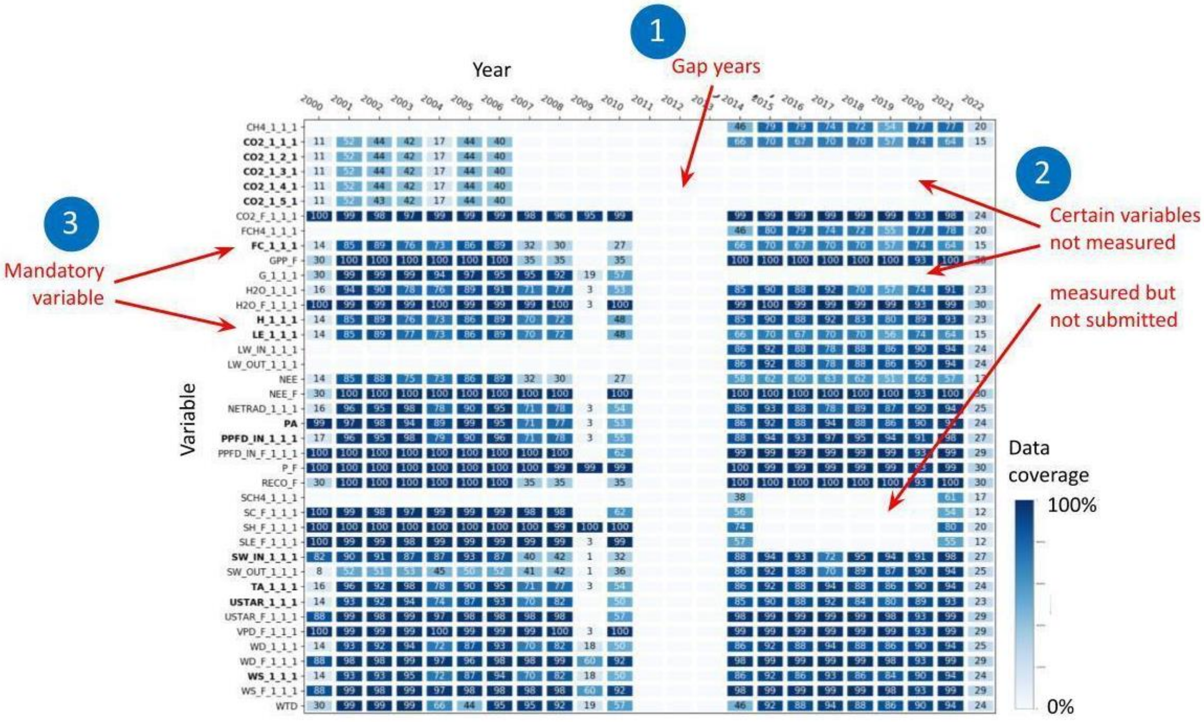

Supplementary Figure S17. Example figure illustrating the data coverage check. The figure shows the variable coverage (color gradient) by year. The example shows three inactive years when all variables are missing (1), and the periods when certain variables are not measured or submitted (2). The mandatory variables that are used for further ONEFlux processing are highlighted in bold (3).

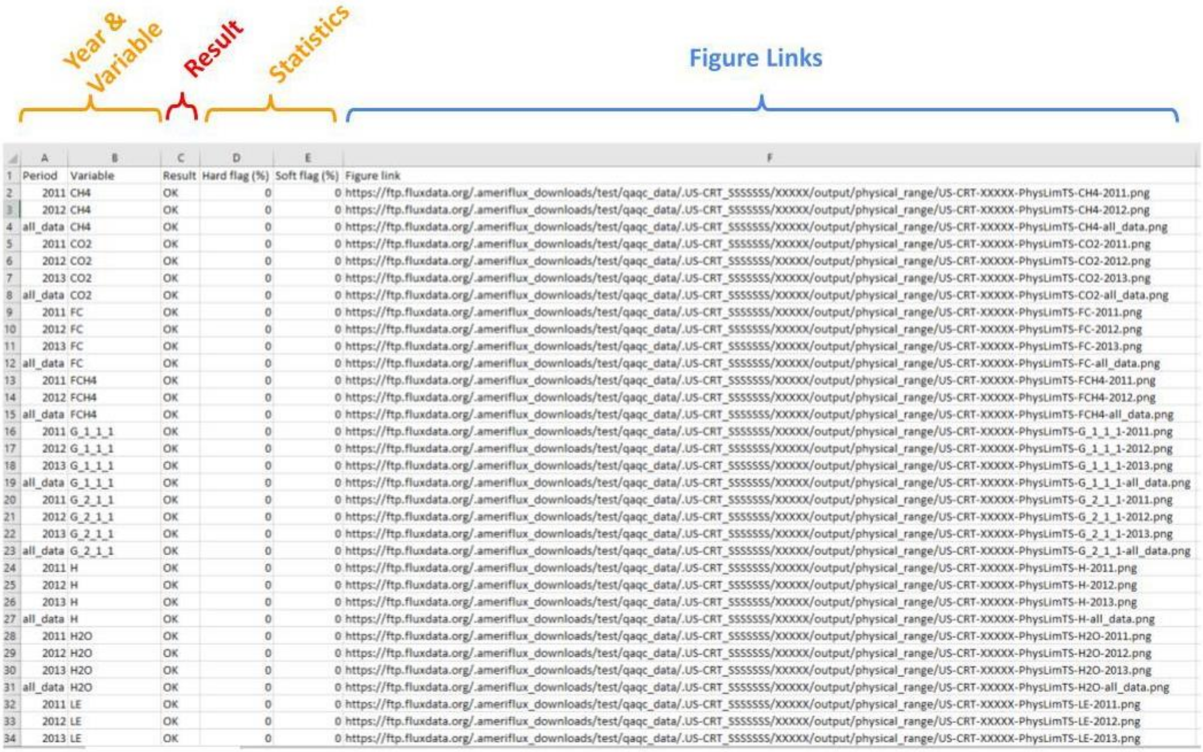

	A	B	C	D	E	F
1	Period	Variable	Result	Hard flag (%)	Soft flag (%)	Figure link
2	2011	CH4	OK	0	0	https://ftp.fluxdata.org/.ameriflux_downloads/test/qaqc_data/.US-CRT_SSSSSSS/XXXXX/output/physical_range/US-CRT-XXXXX-PhysLimTS-CH4-2011.png
3	2012	CH4	OK	0	0	https://ftp.fluxdata.org/.ameriflux_downloads/test/qaqc_data/.US-CRT_SSSSSSS/XXXXX/output/physical_range/US-CRT-XXXXX-PhysLimTS-CH4-2012.png
4	all_data	CH4	OK	0	0	https://ftp.fluxdata.org/.ameriflux_downloads/test/qaqc_data/.US-CRT_SSSSSSS/XXXXX/output/physical_range/US-CRT-XXXXX-PhysLimTS-CH4-all_data.png
5	2011	CO2	OK	0	0	https://ftp.fluxdata.org/.ameriflux_downloads/test/qaqc_data/.US-CRT_SSSSSSS/XXXXX/output/physical_range/US-CRT-XXXXX-PhysLimTS-CO2-2011.png
6	2012	CO2	OK	0	0	https://ftp.fluxdata.org/.ameriflux_downloads/test/qaqc_data/.US-CRT_SSSSSSS/XXXXX/output/physical_range/US-CRT-XXXXX-PhysLimTS-CO2-2012.png
7	2013	CO2	OK	0	0	https://ftp.fluxdata.org/.ameriflux_downloads/test/qaqc_data/.US-CRT_SSSSSSS/XXXXX/output/physical_range/US-CRT-XXXXX-PhysLimTS-CO2-2013.png
8	all_data	CO2	OK	0	0	https://ftp.fluxdata.org/.ameriflux_downloads/test/qaqc_data/.US-CRT_SSSSSSS/XXXXX/output/physical_range/US-CRT-XXXXX-PhysLimTS-CO2-all_data.png
9	2011	FC	OK	0	0	https://ftp.fluxdata.org/.ameriflux_downloads/test/qaqc_data/.US-CRT_SSSSSSS/XXXXX/output/physical_range/US-CRT-XXXXX-PhysLimTS-FC-2011.png
10	2012	FC	OK	0	0	https://ftp.fluxdata.org/.ameriflux_downloads/test/qaqc_data/.US-CRT_SSSSSSS/XXXXX/output/physical_range/US-CRT-XXXXX-PhysLimTS-FC-2012.png
11	2013	FC	OK	0	0	https://ftp.fluxdata.org/.ameriflux_downloads/test/qaqc_data/.US-CRT_SSSSSSS/XXXXX/output/physical_range/US-CRT-XXXXX-PhysLimTS-FC-2013.png
12	all_data	FC	OK	0	0	https://ftp.fluxdata.org/.ameriflux_downloads/test/qaqc_data/.US-CRT_SSSSSSS/XXXXX/output/physical_range/US-CRT-XXXXX-PhysLimTS-FC-all_data.png
13	2011	FCH4	OK	0	0	https://ftp.fluxdata.org/.ameriflux_downloads/test/qaqc_data/.US-CRT_SSSSSSS/XXXXX/output/physical_range/US-CRT-XXXXX-PhysLimTS-FCH4-2011.png
14	2012	FCH4	OK	0	0	https://ftp.fluxdata.org/.ameriflux_downloads/test/qaqc_data/.US-CRT_SSSSSSS/XXXXX/output/physical_range/US-CRT-XXXXX-PhysLimTS-FCH4-2012.png
15	all_data	FCH4	OK	0	0	https://ftp.fluxdata.org/.ameriflux_downloads/test/qaqc_data/.US-CRT_SSSSSSS/XXXXX/output/physical_range/US-CRT-XXXXX-PhysLimTS-FCH4-all_data.png
16	2011	G_1_1_1	OK	0	0	https://ftp.fluxdata.org/.ameriflux_downloads/test/qaqc_data/.US-CRT_SSSSSSS/XXXXX/output/physical_range/US-CRT-XXXXX-PhysLimTS-G_1_1_1-2011.png
17	2012	G_1_1_1	OK	0	0	https://ftp.fluxdata.org/.ameriflux_downloads/test/qaqc_data/.US-CRT_SSSSSSS/XXXXX/output/physical_range/US-CRT-XXXXX-PhysLimTS-G_1_1_1-2012.png
18	2013	G_1_1_1	OK	0	0	https://ftp.fluxdata.org/.ameriflux_downloads/test/qaqc_data/.US-CRT_SSSSSSS/XXXXX/output/physical_range/US-CRT-XXXXX-PhysLimTS-G_1_1_1-2013.png
19	all_data	G_1_1_1	OK	0	0	https://ftp.fluxdata.org/.ameriflux_downloads/test/qaqc_data/.US-CRT_SSSSSSS/XXXXX/output/physical_range/US-CRT-XXXXX-PhysLimTS-G_1_1_1-all_data.png
20	2011	G_2_1_1	OK	0	0	https://ftp.fluxdata.org/.ameriflux_downloads/test/qaqc_data/.US-CRT_SSSSSSS/XXXXX/output/physical_range/US-CRT-XXXXX-PhysLimTS-G_2_1_1-2011.png
21	2012	G_2_1_1	OK	0	0	https://ftp.fluxdata.org/.ameriflux_downloads/test/qaqc_data/.US-CRT_SSSSSSS/XXXXX/output/physical_range/US-CRT-XXXXX-PhysLimTS-G_2_1_1-2012.png
22	2013	G_2_1_1	OK	0	0	https://ftp.fluxdata.org/.ameriflux_downloads/test/qaqc_data/.US-CRT_SSSSSSS/XXXXX/output/physical_range/US-CRT-XXXXX-PhysLimTS-G_2_1_1-2013.png
23	all_data	G_2_1_1	OK	0	0	https://ftp.fluxdata.org/.ameriflux_downloads/test/qaqc_data/.US-CRT_SSSSSSS/XXXXX/output/physical_range/US-CRT-XXXXX-PhysLimTS-G_2_1_1-all_data.png
24	2011	H	OK	0	0	https://ftp.fluxdata.org/.ameriflux_downloads/test/qaqc_data/.US-CRT_SSSSSSS/XXXXX/output/physical_range/US-CRT-XXXXX-PhysLimTS-H-2011.png
25	2012	H	OK	0	0	https://ftp.fluxdata.org/.ameriflux_downloads/test/qaqc_data/.US-CRT_SSSSSSS/XXXXX/output/physical_range/US-CRT-XXXXX-PhysLimTS-H-2012.png
26	2013	H	OK	0	0	https://ftp.fluxdata.org/.ameriflux_downloads/test/qaqc_data/.US-CRT_SSSSSSS/XXXXX/output/physical_range/US-CRT-XXXXX-PhysLimTS-H-2013.png
27	all_data	H	OK	0	0	https://ftp.fluxdata.org/.ameriflux_downloads/test/qaqc_data/.US-CRT_SSSSSSS/XXXXX/output/physical_range/US-CRT-XXXXX-PhysLimTS-H-all_data.png
28	2011	H2O	OK	0	0	https://ftp.fluxdata.org/.ameriflux_downloads/test/qaqc_data/.US-CRT_SSSSSSS/XXXXX/output/physical_range/US-CRT-XXXXX-PhysLimTS-H2O-2011.png
29	2012	H2O	OK	0	0	https://ftp.fluxdata.org/.ameriflux_downloads/test/qaqc_data/.US-CRT_SSSSSSS/XXXXX/output/physical_range/US-CRT-XXXXX-PhysLimTS-H2O-2012.png
30	2013	H2O	OK	0	0	https://ftp.fluxdata.org/.ameriflux_downloads/test/qaqc_data/.US-CRT_SSSSSSS/XXXXX/output/physical_range/US-CRT-XXXXX-PhysLimTS-H2O-2013.png
31	all_data	H2O	OK	0	0	https://ftp.fluxdata.org/.ameriflux_downloads/test/qaqc_data/.US-CRT_SSSSSSS/XXXXX/output/physical_range/US-CRT-XXXXX-PhysLimTS-H2O-all_data.png
32	2011	LE	OK	0	0	https://ftp.fluxdata.org/.ameriflux_downloads/test/qaqc_data/.US-CRT_SSSSSSS/XXXXX/output/physical_range/US-CRT-XXXXX-PhysLimTS-LE-2011.png
33	2012	LE	OK	0	0	https://ftp.fluxdata.org/.ameriflux_downloads/test/qaqc_data/.US-CRT_SSSSSSS/XXXXX/output/physical_range/US-CRT-XXXXX-PhysLimTS-LE-2012.png
34	2013	LE	OK	0	0	https://ftp.fluxdata.org/.ameriflux_downloads/test/qaqc_data/.US-CRT_SSSSSSS/XXXXX/output/physical_range/US-CRT-XXXXX-PhysLimTS-LE-2013.png

Supplementary Figure S18. An example of the summary statistics table from the physical range module. The table contains variable names, periods, results, statistics, and associated figure links.

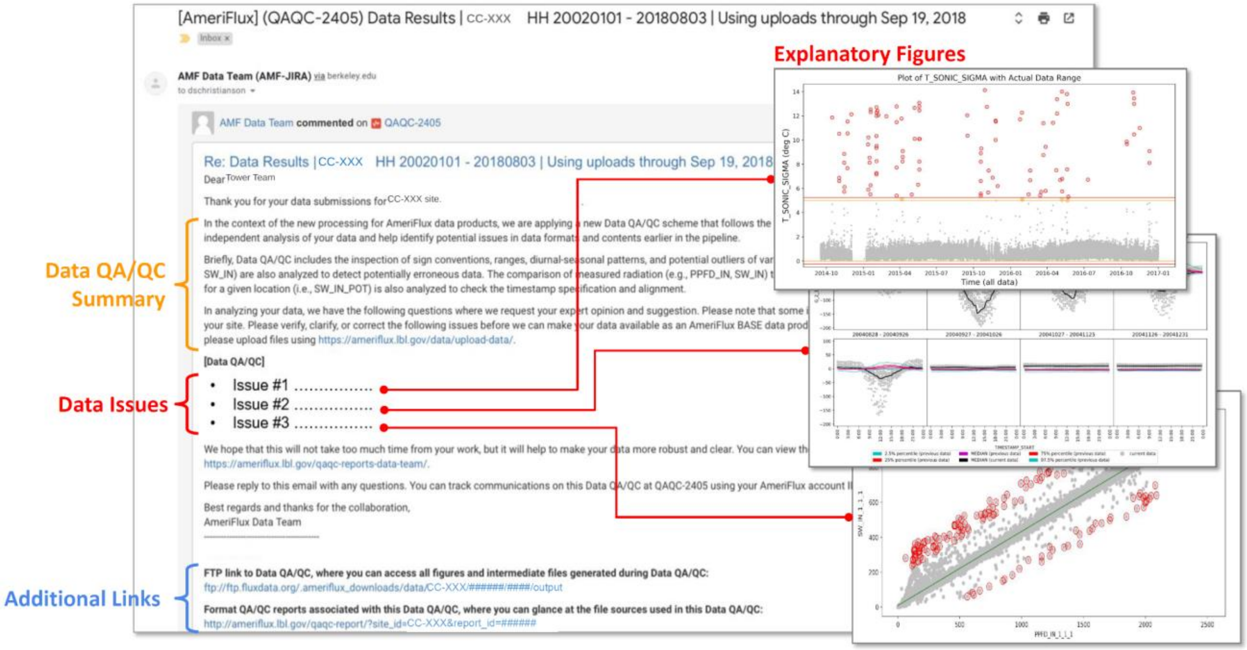

Supplementary Figure S19. An example of a typical Data QA/QC report email. The colored texts highlight the components of the email. The report explains the background of Data QA/QC and summarizes the identified issues with links to explanatory figures. The report also contains links to all statistics (see Supplementary Figure S18), figures (see Supplementary Figures S4-S17), and the Format QA/QC report (see Supplementary Figure S3) associated with the data. The AMP team emails the Data QA/QC report to the site team for clarification or correction.
